# Solvent-Induced Transient Self-Assembly of Peptide Gels: Gelator–Solvent Reactions and Material Properties Correlation

**DOI:** 10.1021/acs.chemmater.3c02327

**Published:** 2023-12-15

**Authors:** Romain Chevigny, Henna Rahkola, Efstratios D. Sitsanidis, Elsa Korhonen, Jennifer R. Hiscock, Mika Pettersson, Maija Nissinen

**Affiliations:** † Department of Chemistry, Nanoscience Center, University of Jyväskylä, P.O. Box 35, FI-40014 Jyväskylä, Finland; ‡ School of Physical Sciences, University of Kent, Canterbury, Kent CT2 7NH, U.K.

## Abstract

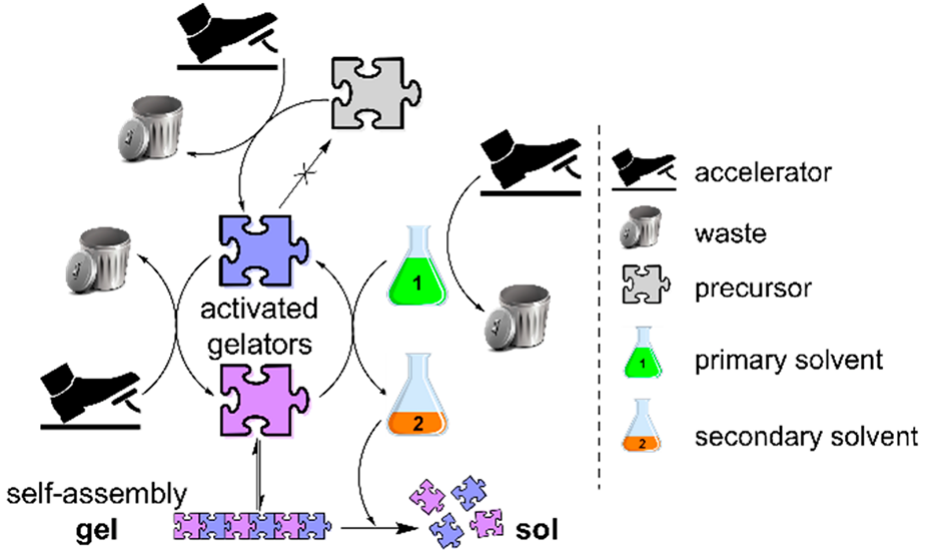

Herein, we introduce a new methodology for designing transient organogels that offers tunability of the mechanical properties simply by matching the protective groups of the precursor to that of the solvent. We developed solvent-induced transient materials in which the solvent chemically participates in a set of reactions and actively supports the assembly event. The activation of a single precursor by an acid (accelerator) yields the formation of two distinct gelators and induces gelation. The interconversion cycle is supplied by the secondary solvent (originating from hydrolysis of the primary solvent by the accelerator), which then progressively solubilizes the gel network. We show that this gelation method offers a direct correlation between the mechanical and transient properties by modifying the chemical structure of the precursors and the presence of an accelerator in the system. Such a method paves the way for the design of self-abolishing and mechanically tunable materials for targeted purposes. The biocompatibility and versatility of amino acid-based gelators can offer a wide range of biomaterials for applications requiring a controllable and definite lifetime such as drug delivery platforms exhibiting a burst release or self-abolishing cell culture substrates.

## Introduction

Biological supramolecular assemblies, which exist out of equilibrium, are under the constant exchange of energy and matter with their environment to sustain the transient state,^1−3^ in contrast to in-equilibrium biological assemblies. These systems often exhibit interesting properties, such as a triggerable response to external stimuli and self-healing.^4,5^ In contrast to biological assemblies, analogous artificial counterparts form under thermodynamic equilibrium, i.e., the intermolecular interactions, structure, and inner environment are kept stable when no external stimuli/disturbance is applied. In an effort to mimic naturally occurring systems and the unique properties of out-of-equilibrium systems, transient supramolecular materials have gained momentum in the past decades.^4,6^ Transient materials have been envisioned to be used as temporary delivery devices, such as drug delivery platforms^7−9^ and self-abolishing materials.^4^ Therefore, transient organogels can enrich the pool of materials with tunable properties such as finite and controllable release.

**Scheme 1:**
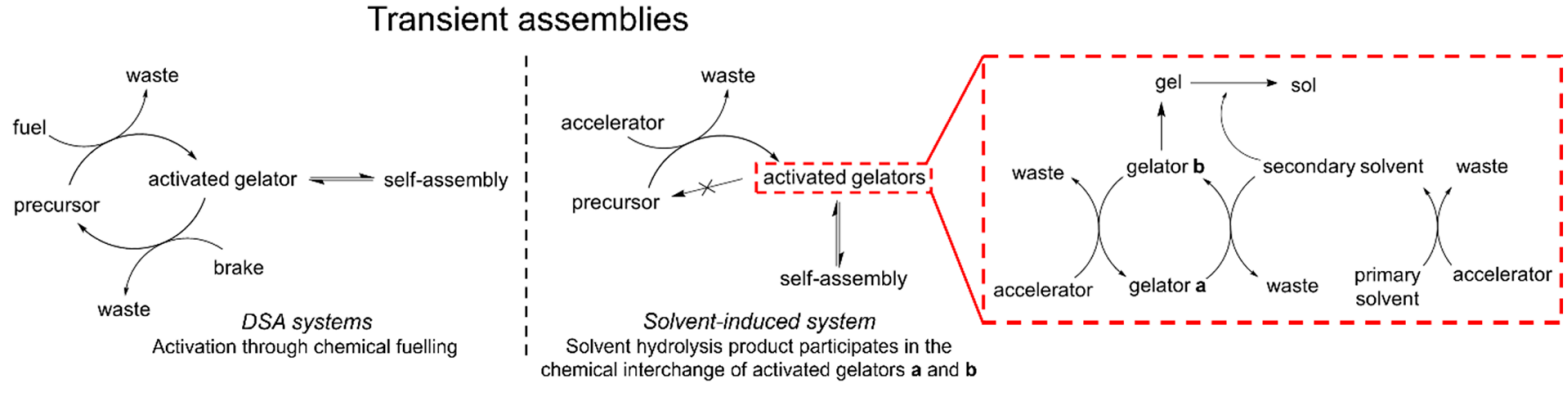
Schematic Description of DSA and Solvent-Induced Self-Assembly Systems The inset represents the set of reactions leading to self-assembly and gel-to-sol transition.

Dissipative (or dynamic) self-assembly (DSA) is an extensively studied representative of transient assembly mode, which relies on a reaction cycle,^10−12^ such as that exemplified in Scheme 1. Consumption of energy (i.e., fuel) by a gelator precursor forms the activated building blocks (activation reaction), which subsequently self-assemble.^13−15^ The transient assembled structure exhibits a limited lifetime governed by depletion of the fuel. Energy dissipation reverses the process, regenerating the non-assembling gelator precursor (deactivation reaction) and inducing the collapse of the network (disassembly). Thus, transient assemblies occur when the rate of energy consumption is higher than that of energy dissipation. Among DSA systems, several types of fuel have been previously reported, including chemical, light, and electrical fueling.^16−18^ Chemically fueled DSA systems intrinsically produce chemical waste during the DSA reaction cycle, that is, side products after activation and/or deactivation reactions, which can be problematic in some applications. Light-fueled DSA systems, which do not release chemical waste into the system, were developed to overcome this problem.^16^

Recently, our group introduced the solvent as a new type of chemical agent inducing a transient assembly^19^ inspired by DSA. In both dissipative and non-dissipative self-assembly, solvents are encapsulated in the gel network and do not affect the assembly event via chemical reactions. Our “solvent-induced” supramolecular gel (Scheme 1) is a rare case in which the primary solvent, *tert*-butyl acetate (*t*BuOAc), actively controls a set of reactions promoting self-assembly. In contrast to typical DSA, the deprotection of the gelator precursor by an acid accelerator (activation reaction) gives rise to two distinct gelators. The *N*-Boc protective group is irreversibly deprotected, while the *t*Bu ester group at the *C*-terminus is reversibly deprotected. Both gelators interconvert through a cycle of hydrolysis–esterification reactions supplied by the secondary solvent *tert*-butyl alcohol (*t*BuOH), a hydrolysis product of the primary solvent, reintroducing the *tert*-butyl group in the cycle. A key requirement for the proposed mechanism is that the primary solvent and the precursor gelator should have the same protective group, *t*Bu ester, in our case. Interestingly, one of the gelators is soluble in *t*BuOH. Therefore, progressive dissolution of the network is observed over time. This new class of solvent-induced self-assembly is not to be defined as DSA. The transient assembly is induced by two acid-accelerated processes: the primary solvent hydrolysis generating the secondary solvent and the gelators’ interconversion. The transient nature of the system relies on the dual role adopted by the solvent, most importantly, the competing kinetics of the secondary solvent formation and the formation/dissolution of the gelators, dictating the assembly/disassembly.

**Figure 1:**
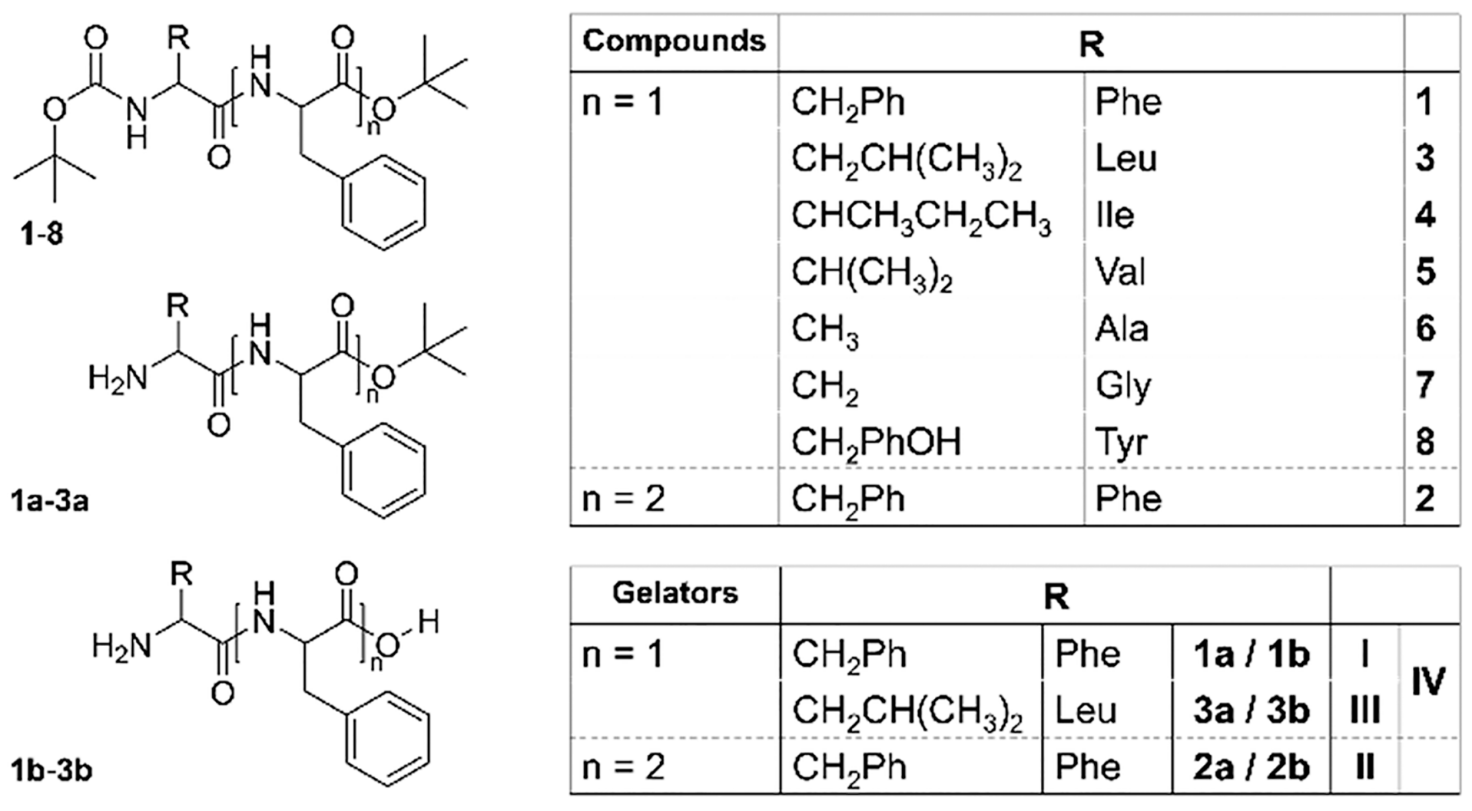
General structures of potential precursors **1**–**8** (upper table), monoprotected gelators **1a**–**3a**, and deprotected gelators **1b**–**3b** (lower table) as well as the corresponding gel systems **I**–**IV**.

Herein, we deepen and further optimize the solvent-induced transient assembly concept and prove its application to a set of phenylalanine-based peptide precursors bearing the same protective group (*t*Bu) as that of the solvent. We report the transient assembly mechanism and assess the effect of the gelators’ chemical structure on the corresponding materials’ properties from the molecular to macroscopic length scale by varying the number of aromatic units (Figure 1). In addition, we decipher the assembly mode and the transitivity character of a multicomponent gel compared to its respective single-component gels. We show that the materials’ intrinsic and transitivity properties are tunable, depending on the gelation conditions and gelator structure, thus paving the way toward controllable materials.

## Experimental Section

### Materials

All chemicals were used as supplied without any further purification, unless stated otherwise. (*S*)-Phenylalanine *tert*-butyl ester HCl (Phe-O*t*Bu) and *N*-(*tert*-butoxycarbonyl)-l-tyrosine (Boc-Tyr) were purchased from Carbosynth. *N*-(*tert*-Butoxycarbonyl)-l-phenylalanine (Boc-Phe), *N*-(*tert*-butoxycarbonyl)-l-valine (Boc-Val), and *N*-(*tert*-butoxycarbonyl)-alanine OH (Boc-Ala) were purchased from TCI. *N*-(*tert*-Butoxycarbonyl)-leucine OH (Boc-Leu), *N*-(*tert*-butoxycarbonyl)-isoleucine OH (Boc-Ile), and *N*-(*tert*-butoxycarbonyl)-glycine OH (Boc-Gly) were purchased from Sigma-Aldrich. 2-(1*H*-Benzotriazole-1-yl)-1,1,3,3-tetramethylammonium tetrafluoroborate (TBTU) and sodium hydrogen carbonate (NaHCO_3_) were purchased from Novabiochem and VWR Chemicals, respectively. *tert*-Butyl acetate (*t*BuOAc) was purchased from TCI and sulfuric acid from Fluka.

### Methods

#### NMR Spectroscopy

The spectra of the synthesized compounds and xerogels were recorded on Bruker Advance III HD 300 and 500 MHz spectrometers in *d*_6_-DSMO and CDCl_3_ solvents. Chemical shifts (δ) are given in parts per million, and the coupling constant (*J*) is given in Hz. The spectra were referenced to the solvent signal (2.5 and 7.26 ppm in ^1^H NMR and 39.52 and 77.16 ppm in ^13^C NMR for *d*_6_-DSMO and CDCl_3_, respectively). ^13^C NMR spectra were recorded with a broadband ^1^H decoupling. Xerogels were obtained from fresh organogels dried in open air at room temperature (overnight).

#### FTIR Spectroscopy

The spectra of the synthesized compounds and xerogels were recorded on a Bruker Tensor 27 FT-IR in attenuated total reflectance (ATR) mode. Spectral width: 400–4000 cm^–1^; number of scans: 124; resolution: 4 cm^–1^. All spectra were baseline corrected. Xerogels were prepared as mentioned above in the NMR Spectroscopy section.

#### UV–Vis Spectroscopy

The UV–vis spectra of synthesized compounds in ACN and gels were recorded on a PerkinElmer Lambda 850 UV–vis spectrometer (spectral range: 200–500 nm; step: 1 nm; integration time: 0.2 s; slit width: 2 nm). The samples were measured in a 1 mm path length quartz cuvette at room temperature. Gel samples were prepared *in situ* in the quartz cuvettes one day before the measurements.

#### Rheology

Rheology measurements were performed on an Anton Paar MCR 302 modular compact rheometer with an upper geometry cylinder (cylinder-relative ST10-4V-8.8/97.5). Gel samples (1.0 mL) were prepared in glass vials (Fisherbrand Type III soda lime glass, 14 mm inner diameter) and allowed to rest for one day before measurements for gelation to occur. Frequency sweep measurements were performed within the linear viscoelastic region (LVR) as obtained by amplitude sweep measurements. All measurements were taken in triplicate at room temperature.

#### SEM Imaging

Microscopy images were obtained on a Zeiss EVO-50XVP microscope. Diluted gels (×10), with the organic solvent used for gelation, were pipetted (1 μL) onto carbon films (400 mesh copper grids) obtained from Agar Scientific and freeze-dried for 1 h before imaging.

## Results and Discussion

### Transient Assembly at the Molecular Level

Phenylalanine-derived peptide precursors **1**–**8** (Figure 1) bearing a phenylalanine unit and varying the second unit by using hydrophobic side-chained amino acids were synthesized (see the Supporting Information, Section 1, for synthesis details).

**Figure 2:**
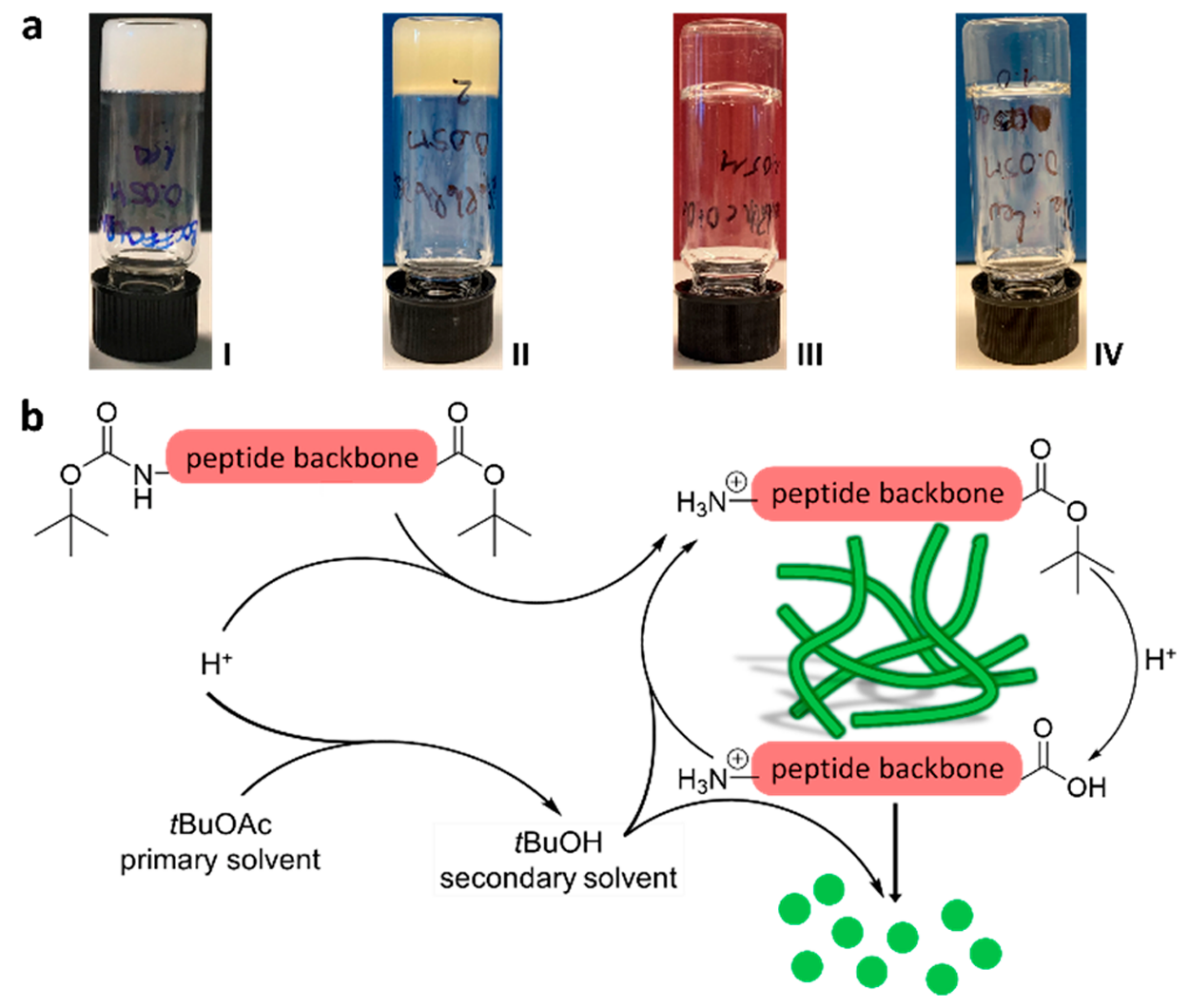
(a) Vial inversion test confirming the lack of flow and thus acting as supporting evidence of material formation for gel systems **I**, **II**, **III**, and **IV**. Gel **IV** is the multicomponent gel formed by precursors **1** and **3** with a 1:1 molar ratio. (b) Schematic depiction of the solvent-induced transient assembly.

Under identical gelation conditions (*t*BuOAc solvent, 50 mM, 1 equiv of sulfuric acid), four self-supporting gels (SSG) were obtained (Figure 2a). Interestingly, no SSG formed from the precursors bearing tyrosine or an aliphatic side chain shorter than leucine. Although the gelation ability of specific molecules under certain conditions is not fully understood, common trends and hypotheses can be drawn. The presence of phenylalanine (Phe), tyrosine (Tyr), and leucine (Leu) motifs has been reported to play an important role in the self-assembly event. Because of the hydrophobicity, the π–π stacking tendency of Phe and Tyr, and the additional hydrogen-bonding donor site of Tyr, these aromatic amino acids are often present in amino acid-based gelators. Similarly, Leu is widely reported as an efficient amino acid for gelation due to its mobile aliphatic side chain.^20−22^ In our case, the length of the side chain and the electric charge affect the gelation process by electrostatic repulsion and/or steric hindrance^23^ which is in line with the existing literature.

The only precursors yielding SSGs (Figure 2a) contain either only phenylalanine or phenylalanine and leucine motifs (Boc-Phe-Phe-O*t*Bu **1** (gel **I**), Boc-Phe-Phe-Phe-O*t*Bu **2** (gel **II**), and Boc-Leu-Phe-O*t*Bu **3** (gel **III**); Figure 1). In addition, the multicomponent gel (gel **IV**), consisting of precursors **1** and **3** with a 1:1 ratio, was prepared to study whether the mode of assembly is self-sorting (two distinct fibrous networks of each gelator individually) or co-assembly (a single fibrous network consisting of both gelators).^24^ The comparison of precursors **1**, **2**, and **3** also gives insight into the effect of the number of aromatic units on the gelation ability and gel properties.

For all systems (**I**, **II**, **III**, and **IV**), SSGs could be obtained at a concentration down to 25 mM (Tables S2–S5). The phase-transition temperatures (Table S1) show an increase proportional to the number of aromatic units, suggesting that the network stiffens by increasing the aromatic character of the precursors. Importantly, under similar gelation conditions (50 mM, 1.0 equiv of accelerator), each gel system exhibits a transient character with a different lifetime. Gel **I** is stable for 4–6 days and gel **III** for 20–22 days, while multicomponent gel **IV** collapses after 10 days, showing a lifetime approximately in between that of its individual components. For gel **II**, the gel-to-sol transition is observed after 8 days.

**Figure 3:**
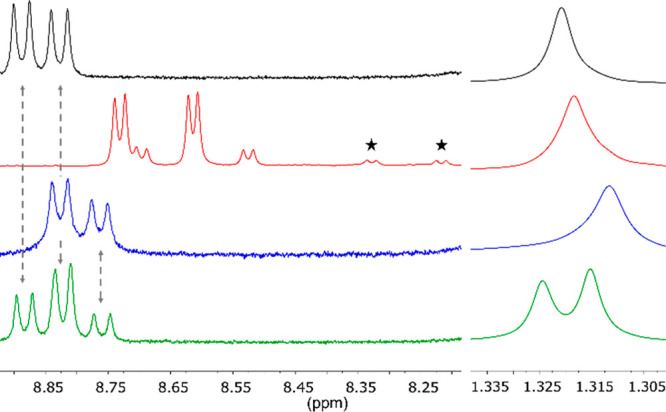
^1^H NMR (300 MHz, *d*_6_-DMSO) spectra of the dried gel systems **I** (black), **II** (red), **III** (blue), and **IV** (green) using 1.0 equiv of accelerator. Peaks belonging to the rotamer are marked by a star.

To assess whether precursors **2** and **3** follow the same transient assembly mechanism as for **1** (Figure 2b), nuclear magnetic resonance (NMR) studies were performed on the xerogels (dried gels) of each system a day after gelation (Figure 3). Gel **I** shows two amide signals at 8.83 and 8.89 ppm corresponding to gelators **1a** and **1b** and a singlet at 1.32 ppm attributed to the *t*Bu ester group of **1a** (Figure 3, black).^19^ Consequently, for gel **III** (Figure 3, blue), an identical trend is observed with the formation of **3a** and **3b**. As precursor **2** contains an additional amino acid unit compared to **1** and **3**, two amide signals are expected per gelator present in gel **II** (Figure 3, red). The peaks marked with an asterisk correspond to a rotamer of **2a** observed in *d*_6_-DMSO (Figure S5). Increasing temperature provides additional energy lifting the rotational restriction of the single bond. Therefore, ^1^H NMR spectra recorded at 30, 70, and 90 °C show the coalescence of the rotamer peaks (Figure S6). Additionally, the presence of the rotamer was confirmed by recording the spectrum in a different solvent, here, CDCl_3_, in which no additional signals were observed (Figure S3). HR-MS also confirmed the presence of a single molecule.

At the amide region, the spectrum of multicomponent gel **IV** (Figure 3, green) is the superimposition of its individual component’s gel systems (Figure 3, black and blue). As precursors **1** and **3** are present, four gelator molecules are observed in the gel state. Indeed, three amide signals (including the overlapping signal at 8.83 ppm corresponding to two amide groups) and two *t*Bu ester signals are seen. The results are supported by HR-MS measurements confirming four distinct gelators **1a**, **1b**, **3a**, and **3b**. In all gels’ NMR spectra, a broad peak at around 3–6 ppm corresponds to *t*BuOH, as supported by the control experiment of induced hydrolysis of *t*BuOAc (Figure S26). In addition, NMR spectra were measured on the xerogels of each system at half of their respective lifetimes. All systems follow an identical trend; that is, the expected gelators are present in the corresponding gels, although in a different ratio (Figures S35, S41, and S47). These results suggest that throughout the lifetime of the gels, the chemical interchange carries on until the materials collapse.

Surprisingly, all systems formed SSGs also when a lower amount of accelerator was used to trigger gelation (0.5 equiv instead of 1.0 equiv). Analysis of the ^1^H NMR spectra of these gels revealed that the same reactions occurred, as the spectra are quantitatively similar. However, since half the amount of triggering agent (acid accelerator) was used, the remains of the unreacted precursors were also present in the systems (Figures S29, S33, S39, and S45). Notably, because less accelerator was used to trigger the hydrolysis cycle (required water originates from the aqueous accelerator), a smaller amount of *t*BuOH formed during gelation. Therefore, gels made with 0.5 equiv of accelerator do not show a transient character, unlike those made with 1.0 equiv, and are stable to date. Respectively, the lifetime of the gels formed using 1.5 equiv of accelerator was shorter (Tables S2–S5). A higher concentration of accelerator produces more *t*BuOH in the medium, increasing the collapse rate of the materials from 4–10 days to 1–3 days.

Additionally, the effect of the precursor concentration on the dynamic properties (lifetime) of the materials was investigated (Tables S2–S5). It was found that gels with a high precursor concentration (100 mM) exhibited longer lifetimes than those with a lower concentration (50 mM). This difference is presumably due to the higher amount of gelator to be dissolved and stronger supramolecular interactions maintaining the assembly.

To verify the assumption that the gelation trigger and the subsequent gelation mechanism are related only to the identities of the *C*-terminus protective group and solvent, an attempt to induce gelation using the same trigger in dichloromethane (DCM) was made with precursors **1** and **3**. No gelation was observed; instead, the product precipitated, and the analysis of the ^1^H NMR spectrum revealed the complete deprotection of the precursors (Figure S49). In addition, gelation was attempted using Boc-deprotected molecules **1a**–**3a** as precursors using the same trigger. SSGs formed within the same time, and ^1^H NMR spectroscopy verified the presence of both monoprotected and fully deprotected gelators, along with *t*BuOH, in the medium. Therefore, both the solvent and the precursor’s *C-*terminus must bear the same functional group, in this case *t*Bu ester, for the reaction cycle and gelation to occur, regardless of the peptide backbone and/or the presence of a Boc protective group on the N-terminus.

### Higher-Order Assembly Determination

**Figure 4:**
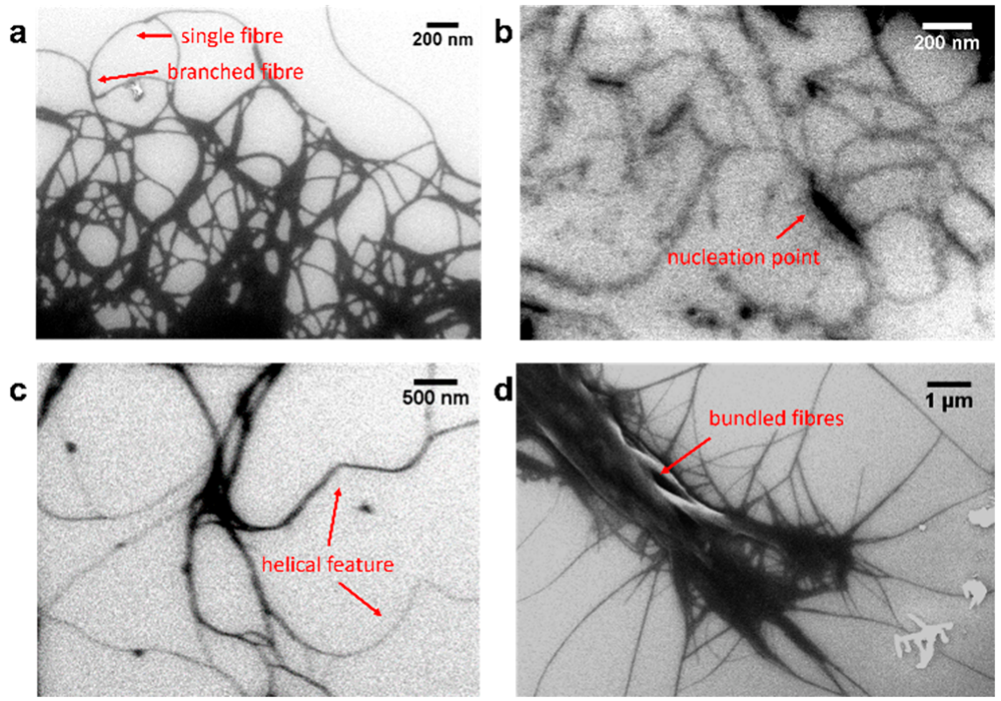
SEM images of (a) gel **I**, (b) gel **II**, (c) gel **III**, and (d) gel **IV**. The final concentration of all gels was 5 mM after dilution with *t*BuOAc. Samples were pipetted onto a silicon surface and freeze-dried for 1 h prior to imaging.

To investigate the effect of the gelators’ structure on the fibrous network, scanning electron microscopy (SEM) images shown in Figure 4 were recorded on diluted gels. All samples were freeze-dried for 1 h prior to imaging to minimize potential structural rearrangement of the network during drying at room temperature.^25^ Gel **I** (Figure 4a) contains densely packed, curved fibers, branching and entangling with one another, which support the high stiffness value measured by rheology (*vide infra*). In contrast, the fibers of gel **III** (Figure 4c), although similar in shape to gel **I**, appear less packed, verifying the lower stiffness than gel **I**. In addition, helical features are present, supporting the peak corresponding to the helical-type assemblies observed in IR measurements (*vide infra*). For multicomponent gel **IV** (Figure 4d), straightened fibers highly entangled into bigger bundles are observed. The resulting fibrous network exhibits a different shape than its individual components, suggesting that a new structure has formed and implying co-assembly of the gelators.^26^ In gel **II**, SEM image shows individual curved fibers branching around nucleation points (Figure 4b).

**Figure 5:**
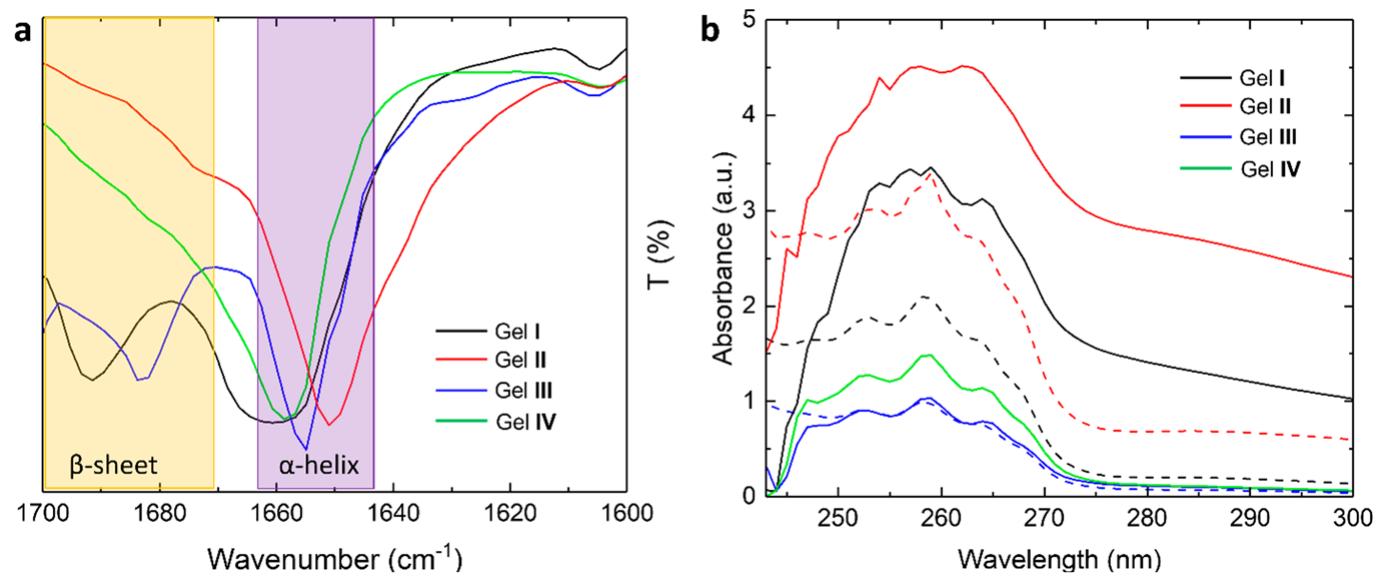
(a) IR spectra of gels **I**–**IV** showing the amide I region (1600–1700 cm^–1^). (b) UV–vis absorption spectra. Dashed curves correspond to the spectra of precursors **1** (black), **2** (red), and **3** (blue) in solution and solid curves to their corresponding gels **I**, **II**, **III**, and **IV** (green). All measurements were performed on gels one day after gelation. For all samples, in solution and gel state, the concentration was 50 mM. The optical path length was 1 mm.

Infrared (IR) spectroscopy was then employed to assess the differences in supramolecular interactions leading to gelation and the structural differences of the gelators. We specifically emphasized on the secondary structures within the amide I region (1700–1600 cm^–1^). The ATR-FTIR spectra of the xerogels were measured one day after gelation (Figure 5a).

Previously reported diphenylalanine (Phe-Phe)-based supramolecular gels mainly exhibit a β-sheet secondary structure.^27−31^ In our case, for gel **I** consisting of protected diphenylalanine gelator molecules **1a** and **1b**, a strong band corresponding to a helical-shaped assembly in the higher organization is observed at 1660 cm^–1^,^32^ in addition to the expected β-sheet band at 1691 cm^–1^ (Figure 5a, black).^33^ The discrepancy with the literature possibly arises from the presence of two different gelators instead of one. The IR spectrum of gel **III** (Figure 5a, blue) is qualitatively similar to that of gel **I** and exhibits both a helical assembly (1655 cm^–1^) and a β-sheet motif (1682 cm^–1^). IR spectra recorded on xerogels and non-dried “wet” gels present similar secondary structures with minor shifts (Figures S63b and S65b). Therefore, the drying conditions do not significantly affect the secondary structures of the gels. Surprisingly, the IR spectrum of gel **II** only shows a band corresponding to a helical assembly at 1651 cm^–1^ (Figure 5a, red). Although previous literature reports that tripeptide (Phe-Phe-Phe)-based amphiphiles form organogels exhibiting β-sheet structures,^34^ this is not observed in our case. Similarly to gel **I**, the discrepancy is possibly due to the presence of a second gelator in the system. Systematic experimental and computational studies report the role and importance of Leu and Phe amino acids in the formation of secondary structures. While Leu is considered a helix former due to the rotational freedom of the γ-branched side chain,^35^ Phe is reported to favor the formation of β-sheets. However, some reports also highlight the importance of aromatic interactions for helix stabilization^36^ and that Phe is not necessarily a helix breaker. Additionally, in our systems, the bulky *tert*-butyl group might also disturb the system as a whole and force it to yield divergent arrangements of the building blocks such as helices.

As gel **IV** is formed by both precursors **1** and **3** with a 1:1 ratio, the IR profile of its xerogel within the amide I region should provide insight into whether self-sorting or co-assembly occurred. If self-sorting occurred, then the mere superimposition of the individual components’ spectra would be observed. On the contrary, a different spectrum would be observed in the case of co-assembly. A peak at 1658 cm^–1^ corresponding to a helical-shaped assembly is observed (Figure 5a, green plot), and unlike the gels of its individual components, no β-sheet motif is clearly visible. This suggests that a new structure has formed by the co-assembly of the different gelators.^37^

In addition to the amide I region, the shifts of specific vibrational bands provide important information on the intermolecular interactions occurring during gelation, i.e., π–π stacking and hydrogen bonding (Table 1). The bands corresponding to the C=C stretching of the phenyl rings appear in the gelators (neat powder) at 1495 cm^–1^ (**1a**), 1496 cm^–1^ (**1b**), 1495 cm^–1^ (**2a**, **2b**), 1497 cm^–1^ (**3a**), and 1496 cm^–1^ (**3b**).^38^ In the gel phase, these bands shift toward shorter wavenumbers to 1493 cm^–1^ (gel **I**), 1494 cm^–1^ (gel **II**), and 1495 cm^–1^ (gel **III** and **IV**), which suggests the π–π stacking of the phenyl rings. Although the small shifts of the C=C bands are of the same magnitude as the resolution limit of the instrument, their presence still indicates the involvement of π–π stacking in intermolecular interactions. Additionally, the bands corresponding to the terminal amine are merged and downshifted (gel **I**: 3328 cm^–1^; gel **II**: 3349 cm^–1^; gel **III**: 3323 cm^–1^; gel **IV**: 3320 cm^–1^), suggesting hydrogen bonding.

**Table 1:** Phenyl Ring C=C Stretching and *N*-Terminal N–H Stretching Wavenumber Peaks (cm^–1^) of the Synthesized Gelators (Neat Powders) and Gel Samples

	C=C stretching phenyl rings			N–H stretching terminal amine		
gel system	gelator **a**	gelator **b**	gel	gelator **a**	gelator **b**	gel
gel I	1495	1496	1493	3392, 3346, 3288	3249, 3171	3328
gel II	1495	1495	1494	3370, 3266	3375, 3363	3349
gel III	1497	1496	1495	3364, 3319	3392, 3340	3323
gel IV	1495, 1497	1496, 1496	1495	3392, 3364, 3346, 3319, 3288	3392, 3340, 3249, 3171	3320

Complementarily to IR, UV–vis spectroscopy provides insights into the molecular packing of the gelators and has been extensively used to elucidate the interactions leading to self-assembly.^39^ More specifically, information about the π–π stacking of the phenylalanine moiety can be obtained, such as the type of aggregates (H or J), by comparison of the band shift between the spectrum of monomers in solution (interaction free) and the spectrum of the gel (aggregated monomers).^40^ The absorption spectra of the gelators for each system were obtained at the same concentration as those of the gels (50 mM) in acetonitrile to enhance solubility (Figure 5b, dashed curves). Solvatochromism (the shift of the absorption maxima depending on the solvent)^41^ and the concentration effect were verified by recording the UV spectra in *t*BuOAc at 50 and 5 mM concentrations.

However, no significant shifts were observed in either case (Figure S50). The center of the absorption band (λ_max_) at 258 nm for precursors **1**–**3** corresponds to the π → π* transition of the phenyl rings. As seen in Figure 5b, the λ_max_ is slightly red-shifted upon gelation to 259 nm for gels **I** and **III** and 260 nm for gel **II** (plain curves). Because of the weakly absorbing character of the phenylalanine residues, compared to, for example, naphthalene and anthracene, the observed shifts are minor (1–2 nm).^42^ However, these results still suggest the π system overlapping in J-aggregate. Additionally, the loss of fine vibronic structure in the spectra of gel **I** and **II** is attributed to charge transfer over molecular aggregation.^43−45^ In our case, in contrast to most of the gel aggregation studies reported,^43,46,47^ we observed an absorption intensity increase over gelation, even after normalization due to scattering. This behavior could be explained by the aggregation-induced enhanced emission also related to the absorption profile.^45,48−50^ The red-edge tails observed for gels **I** and **II** most probably arise from the scattered light from the aggregates (opaque gels). No broadening is observed for gels **III** and **IV** containing just one phenylalanine unit due to its weak π → π* transition. These results correlate with the rheological profile of the gels; the weaker the π–π interactions, the softer the gels. Interestingly, the intensity of the band of gel **IV** is observed between those of its individual component’s gels. Red-shifts of the weaker, longer wavelength n → π* transitions corresponding to the C=O carbonyl and N–H amide groups,^51^ respectively, are observed to be significant as the precursors’ aromaticity increases (**3** < **1** < **2**). This suggests hydrogen-bonding-type interactions from these groups, consistent with the literature.^28,52−54^

### Macroscopic Properties Assessment

**Figure 6:**
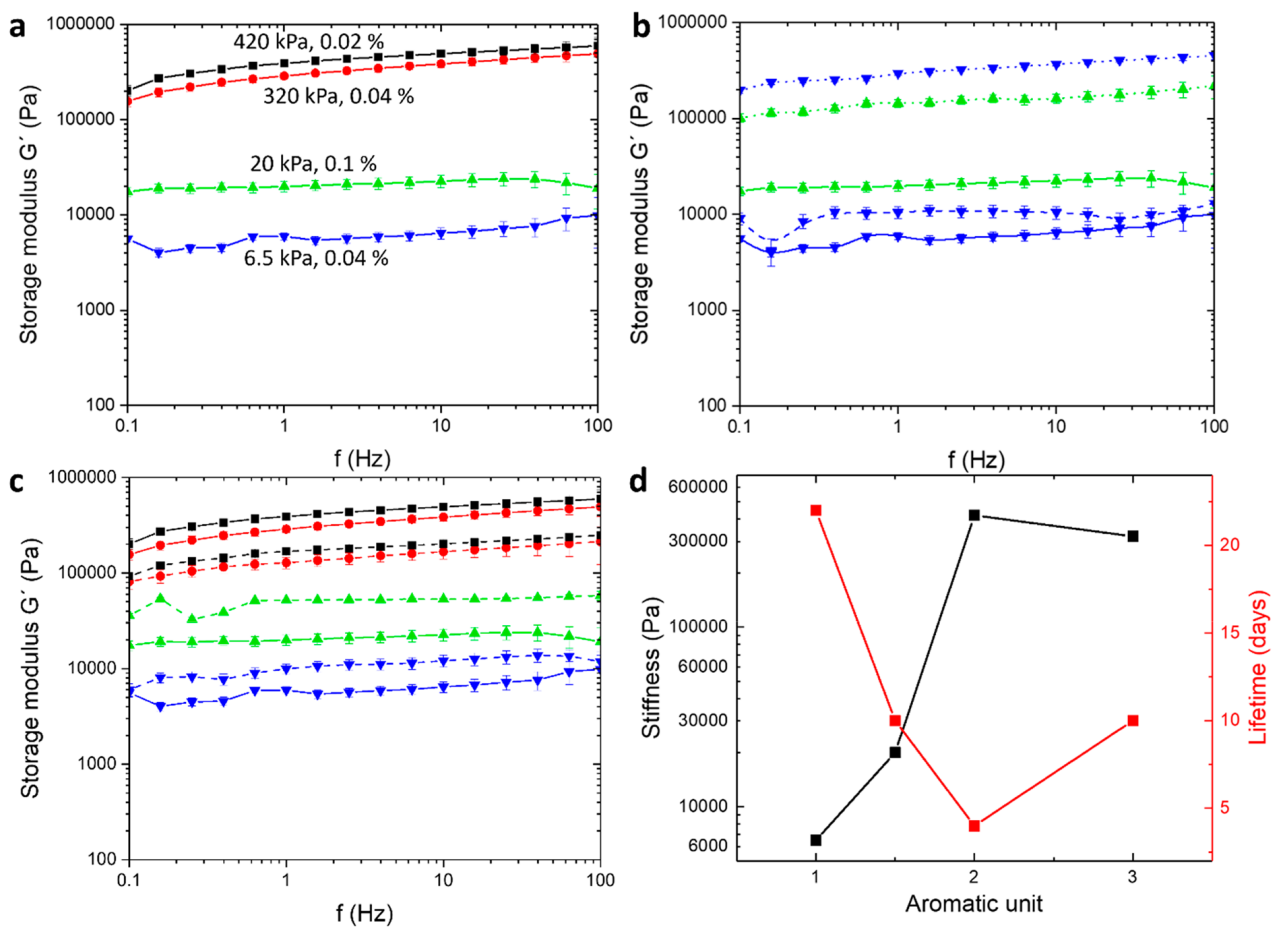
Rheological studies of the gels. (a) Frequency sweep measurements, under a constant shear strain γ (%) of gel **I** (black, γ = 0.02%), gel **II** (red, γ = 0.04%), gel **III** (blue, γ = 0.04%), and gel **IV** (green, γ = 0.1%). The concentration of the gel specimens was 50 mM. (b) Frequency sweep measurements of gel systems **III** (blue curves) and **IV** (green curves) at 25 mM (dashes), 50 mM (solid), and 100 mM (dots). Gelation was triggered with 1.0 equiv of H_2_SO_4_. (c) Frequency sweep measurements of gel **I** (black), **II** (red) **III** (blue), and **IV** (green) triggered by 1.0 equiv (solid) and 0.5 equiv (dashes) of H_2_SO_4_. All gels were diluted to a concentration of 50 mM. (d) Plot of the elastic moduli (black) against the lifetime (red) of the gels as a function of the number of aromatic units in the precursor. Data markers refer to numerical values from experiments. The point at 1.5 aromatic units corresponds to the average amount of aromatic rings in the system per mole of precursor in gel **IV**, where the ratio between **1** and **3** is 1:1. Error bars are calculated by standard deviation. Details of the experimental conditions and amplitude sweep measurements are given in the Experimental Section.

Rheology experiments were performed on the gel systems to assess the correlation between the gelators’ structures and the materials’ mechanical properties. A material exhibiting a greater storage modulus *G*′ than the loss modulus *G*″ upon shear strain is considered “solid”. At the point this inverses, the material is considered “liquid”.^55,56^ For all gel systems, the measured elastic modulus (or storage modulus), *G*′, is greater than the viscous modulus (or loss modulus), *G*″, verifying the viscoelastic nature of the gels. For comparison purposes, Figure 6a presents the oscillatory frequency sweep (FS) experiments performed on gel specimens within the linear viscoelastic region (LVR) one day after gelation at a constant concentration of 50 mM and 1.0 equiv of accelerator. The *G*′ of both gels **I** (black) and **II** (red) is 420 and 320 kPa (Table 2), respectively, which is in the range of the bladder and gut tissue stiffness.^57,58^ These quantitatively similar values suggest that an additional aromatic moiety (in this case, phenylalanine) does not significantly alter the stiffness of the material.

**Table 2:** Comparison of the Stiffness (*G*′) and Elasticity (*G*′/*G*″ Cross Point) Values of Gels **I**–**IV**

		*G*′ (kPa)		*G*′/*G*″ cross point (%)	
	concn (M)	0.5 equiv	1.0 equiv	0.5 equiv	1.0 equiv
gel **I**	0.05	180	420	4.2	1.5
gel **II**	0.05	147	320	no crossing points	
gel **III**	0.025	n.m.^a^	10	n.m.	6.5
	0.05	11	6.5	9	3
	0.1	n.m.	330	n.m.	2
gel **IV**	0.05	52	20	4.5	3.5
	0.1	n.m.	155	n.m.	1.2

a Not measured.

However, for gel **III** (blue) bearing an aliphatic leucine unit instead of an aromatic phenylalanine unit, the *G*′ value of 6.5 kPa indicates that lower aromaticity of the gelators yields a softer material (approximately 60-fold), which is in the range of the lung and liver tissue stiffness.^57,58^ Previous studies show that a difference in the number of hydrophobic aliphatic carbons in the side chains of the gelators also affects the stiffness of the corresponding materials. However, this effect is minor (2–10-fold increase of *G*′ depending on the side chain length) to the observed 60-fold increase when adding three carbons and the aromatic character between leucine and phenylalanine.^59,60^ Despite a lower stiffness, gel **III** exhibits 2-fold more elastic behavior than gel **I** (Table 2). The elasticity was assessed by comparison of the *G*′/*G*″ cross points of the amplitude sweep measurements. A higher elasticity is indicated by the materials’ resistance to shear strain, as the cross point is shifted toward higher shear strain (γ%) values. Interestingly, the amplitude sweep measurements of gel **II** exhibit no crossing points between *G*′ and *G*″, although the curves seem to merge at the maximum shear strain γ of 100%, indicating that a crossing point would be observed over this value, as reported earlier for highly elastic gels.^61^ The additional phenylalanine motif in precursor **2** provides a higher hydrophobicity and more available sites for π–π stacking than that in precursor **1** (two phenylalanine motifs), giving more stability to the molecular aggregation during gelation. Although more intense π–π stacking yields stiffer materials, the *G*′ values are quantitatively similar for gels **I** and **II**, while the elasticity is higher for gel **II** than for gel **I**. This suggests that the fiber arrangement (microenvironment) has more impact on the material properties than the molecular interactions. In addition, the *G*′ value of 20 kPa for the multicomponent gel **IV** (green), although quantitatively closer to gel **III** than gel **I**, suggests that a new structure has formed, implying co-assembly of the gelators.^24^

To further investigate the effect of gelation conditions on the stiffness of the materials, we measured the elastic modulus *G*′ at different concentrations of gel **III** (monocomponent) and gel **IV** (multicomponent, Figure 6b). An increase in the precursor concentration yields a stiffer material in both cases (approximately 10–40-fold higher *G*′ value), along with a decrease in the elastic behavior (Table 2).

Similar experiments were performed by changing the amount of accelerator (0.5 or 1.0 equiv) to determine whether the precursor concentration of the precursor or that of the accelerator has a more significant effect on the materials’ properties. For all four gel systems, although a quantitatively similar *G*′ value was observed (approximately 3-fold, Figure 6c), the elasticity was found to increase by 1.5–2-fold in gels formed with 0.5 equiv of accelerator. Therefore, we suggest that the mechanical properties of the gels are tunable within a wide range of stiffness by modifying the precursor structure, i.e., the number of aromatic rings, concentration, and the amount of accelerator used to trigger gelation.

The phase-transition temperature (*T*_gel–sol_) indicates the thermal stability of the different materials. Gel samples (viscoelastic gel behavior initially confirmed by rheological measurements) were gradually heated by 5 °C increments at 10 min intervals. The vial inversion method was used to verify the gel-to-sol (i.e., the complete transition from self-supporting gel to free-flowing solution) and gel-to-sol-to-gel transitions (Supporting Information, Section 2.4), assuming that after thermal breaking the viscoelastic nature of the gels remains identical with that of the gels initially assessed by oscillatory frequency sweep measurements. The observed *T*_gel–sol_ was found to be proportional to the number of aromatic units within the gelator structure (gel **III**: 40 °C; gel **I**: 45 °C; and gel **II**: 55 °C at 50 mM) and to the gel concentration (10 °C increase from 25 to 100 mM, Table S1 and Figure S51). These results suggest that the fibrous network and consequently the bulk material stiffen with the increase in the aromatic character of the gelators. However, the observed temperature for gel **IV** is lower than that of its individual components, in contrast to the rheological data. This behavior may arise from differences between the fibrous network (related to the *T*_gel–sol_) and the π–π interactions (related to the gel stiffness).^42^

Numerical values of the lifetime (Figure 6d, red *y*-axis) compared to the mechanical properties (Figure 6d, black *y*-axis) of the gels, when plotted as a function of the number of aromatic units in the precursor structure, highlight a common trend. The evolution of the stiffness is found to be inversely proportionally to that of the lifetime of the materials, that is, stiff materials have a short lifetime. To ensure the reproducibility of the measurements and the reliability of the results, lifetime monitoring and rheological measurements were performed in triplicate. Systematic studies will be beneficial in the future to corroborate these findings for an extended set of data (gelation conditions, precursors, and acid equivalent). This observation can be assessed based on two factors: the gelators’ solubility in the secondary solvent, *t*BuOH, and the material’s elasticity. Indeed, control experiments showed that gelators **1a**, **2a**, and **3a** are soluble in *t*BuOH, whereas their deprotected counterparts **1b**, **2b**, and **3b** are insoluble. Therefore, with time, the *in situ* formed *t*BuOH will progressively dissolve the monoprotected gelator **1a**, **2a**, and **3a** until the collapse of the material, as verified and described above. Considering this and the identical reaction cycle for all gel systems depicted in Figure 2b, the mechanical properties of the gels are potential reasons for the different lifetimes. As the fibrous network is progressively dissolved by *t*BuOH, a more elastic material remains self-supporting for a longer period of time before collapsing.

## Conclusion

We developed solvent-induced transient self-assembly for the formation of a transient gel materials. A diprotected precursor molecule forms *in situ* two gelator building blocks (*N*-deprotected/*C*-protected and *N*-/*C*-deprotected) through activation by an accelerator, while the primary solvent chemically participates in the interconversion cycle between the two activated gelators through the generation of the secondary solvent. Additionally, the secondary solvent progressively solubilizes the fibrous network, leading to the collapse of the material over time. The gelation trigger and subsequent mechanism are related only to the precursor’s *C*-terminus protective group and the solvent, therefore offering a wide range of potential gelator candidates for the gelation of peptide-based systems. UV–vis spectroscopic data correlate the strength of the intermolecular interactions to the mechanical properties of the materials. Gels exhibiting greater stiffness showed greater absorption intensity increase and loss of vibronic structure, and *vice versa*, which can further be attributed to the prevalence of the π–π stacking interactions. IR spectroscopy, rheology, thermal stability, and lifetime studies highlighted the co-assembly of the gelators in the multicomponent gel **IV**. Additionally, the lifetimes of the gels are found to be inversely proportional to their mechanical properties. This work provides important insights into the formation of transient peptide-based supramolecular gels. The biocompatibility and versatility of modified amino acid-based gelators can be exploited, along with gelation in water-based solutions, to serve targeted medical purposes. The hydrolysis/esterification cycles proposed in this work could be used for developing hydrogels with a wide range of mechanical and transient properties, using, for instance, external *tert*-butylated compounds as a supplier of the *t*Bu group to support the out-of-equilibrium self-assembly. The solvent-induced self-assembly can, therefore, provide interesting alternatives for responsive gelation and self-abolishing materials requiring a definite lifetime, such as delivery systems exhibiting a burst release. Alternatively, the self-abolishing character of these materials could be used for designing temporary ink devices. Thorough dynamic studies on the kinetics of gelator and secondary solvent formation, for instance, by high-pressure liquid chromatography^14^ would be beneficial to get insights into the in-depth acid-accelerated processes.

## Supporting Information Available

Materials, synthesis and characterization data of precursors and gels, gelation protocol, ^1^H and ^13^C NMR, IR, and UV–vis spectra, rheology amplitude sweep plots, *T*_gel–sol_, gelation concentration screening (PDF)
