# Supplementary material for: Solvent-Induced Transient Self-Assembly of Peptide Gels: Gelator–Solvent Reactions and Material Properties Correlation
Source: Chem Mater. 2023 Dec 15;36(1):407–16. doi: 10.1021/acs.chemmater.3c02327 (PMC10782441; doi:10.1021/acs.chemmater.3c02327)
Supplement: Supplementary file 1 — cm3c02327_si_001.pdf [file cm3c02327_si_001.pdf]

Supporting Information

Solvent-Induced Transient Self-Assembly of Peptide Gels: Gelator-Solvent Reactions and Material Properties Correlation

Romain Chevigny,[†] *Henna Rahkola,*[†] *Efstratios D. Sitsanidis,*[†] *Elsa Korhonen,*[†] *Jennifer R. Hiscock,*[‡] *Mika Pettersson,*[†] *and Maija Nissinen*[*†]

† Department of Chemistry, Nanoscience Center, University of Jyväskylä, P.O. Box 35, FI-40014 JYU Finland

‡ School of Physical Sciences, University of Kent, Canterbury, Kent, CT2 7NH, UK

Table of Contents

1. Synthesis and characterization 3

1.1. Synthesis of diprotected precursors **1-8** 3

1.1.1. Boc-Phe-Phe-O*t*Bu **1** 3

1.1.2. Boc-Phe-Phe-Phe-O*t*Bu **2** 5

1.1.3. Boc-Leu-Phe-O*t*Bu **3** 9

1.1.4. Boc-Ile-Phe-O*t*Bu **4** 10

1.1.5. Boc-Val-Phe-O*t*Bu **5** 12

1.1.6. Boc-Ala-Phe-O*t*Bu **6** 13

1.1.7. Boc-Gly-Phe-O*t*Bu **7** 15

1.1.8. Boc-Tyr-Phe-O*t*Bu **8** 16

1.2. Synthesis of monoprotected compounds **1a-3a** 18

1.2.1. Phe-Phe-O*t*Bu **1a** 18

1.2.2. Phe-Phe-Phe-O*t*Bu **2a** 20

1.2.3. Leu-Phe-O*t*Bu **3a** 21

2 Gelation and gel characterization 23

2.1 Gelation protocol 23

2.2 Gel characterization by NMR 24

2.2.1 Gel system **I**: Precursor Boc-Phe-Phe-O*t*Bu **1** 25

2.2.2 Gel system **II**: Precursor Boc-Phe-Phe-Phe-O*t*Bu **2** 28

2.2.3. Gel system **III**: Precursor Boc-Leu-Phe-O*t*Bu **3** 32

2.2.4. Gel system **IV**: Precursor Boc-Phe-Phe-O*t*Bu **1** + Boc-Leu-Phe-O*t*Bu **3**, 1:1 ratio...........37

2.2.5. Gelation attempt in DCM...43

2.3. Solvatochromism and concentration study by UV-vis...44

2.4. Phase-transition temperature...45

2.5. Gelation concentration screening...46

3. FTIR spectra of compounds **1-8**, **1a-3a** and xerogels **I-IV**...48

4. UV-vis spectra of compounds **1-1a**, **2-2a** and **3-3a**...58

5. Rheology measurement plots of gel systems **I**, **II**, **III** and **IV**...60

1. Synthesis and characterization

1.1. Synthesis of diprotected precursors 1-8

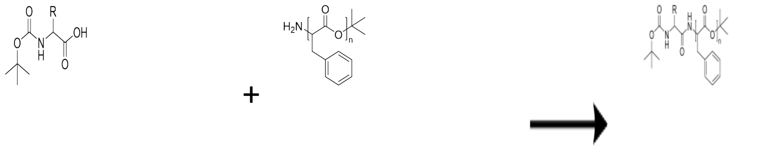

General protocol: Boc-protected amino acid (1.0 equivalent), TBTU (1.0 equivalent) and $NaHCO_3$ (1.0 equivalent) were suspended in anhydrous DMF (10 mL) under N_2 atmosphere. The solution

was left to stir at R.T. for 1 hour. *tert*-butyl protected amino acid (1.1 equivalent) and $NaHCO_3$ (1.1 equivalent) were suspended in anhydrous DMF (10 mL). The two solutions were mixed and allowed to stir under N_2 atmosphere at R.T. overnight. The solvent was then evaporated under vacuum (co-evaporation with toluene x3), and the residue was dissolved in DCM. The organic phase was extracted with water (x2), subsequently with an aqueous HCl solution (0.1 M), water (x2) and a saturated aqueous solution of $NaHCO_3$. The organic phase was then dried with $MgSO_4$ and evaporated under vacuum, yielding the desired compounds.

1.1.1. Boc-Phe-Phe-O*t*Bu 1

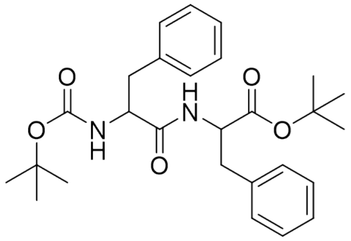

Yellow powder (70 % yield). **Melting point**: 129-133 ˚C.

^{1}H NMR (500 MHz, d_6-DMSO) δ 8.21 (d, *J* = 7.5 Hz, 1H), 7.33 – 7.16 (m, 10H), 6.84 (d, *J* = 8.8 Hz, 1H), 4.38 (q, *J* = 7.3 Hz, 1H), 4.19 (td, *J* = 9.8, 3.9 Hz, 1H), 2.96 (ddt, *J* = 31.5, 13.8, 5.7 Hz, 4H), 2.73 – 2.66 (m, 1H), 1.32 (s, 9H), 1.28 (s, 9H).

^{13}C NMR (126 MHz, d_6-DMSO) δ 171.8, 170.4, 155.2, 138.2, 137.1, 129.3, 129.2, 128.2, 128.0, 126.5, 126.2, 80.7, 78.0, 55.5, 54.1, 37.4, 36.9, 28.1, 27.5.

HR-MS: *m/z* for $C_{27}H_{36}N_2O_5$ $[M + H]^+$ calculated 469.2700, found 469.2675.

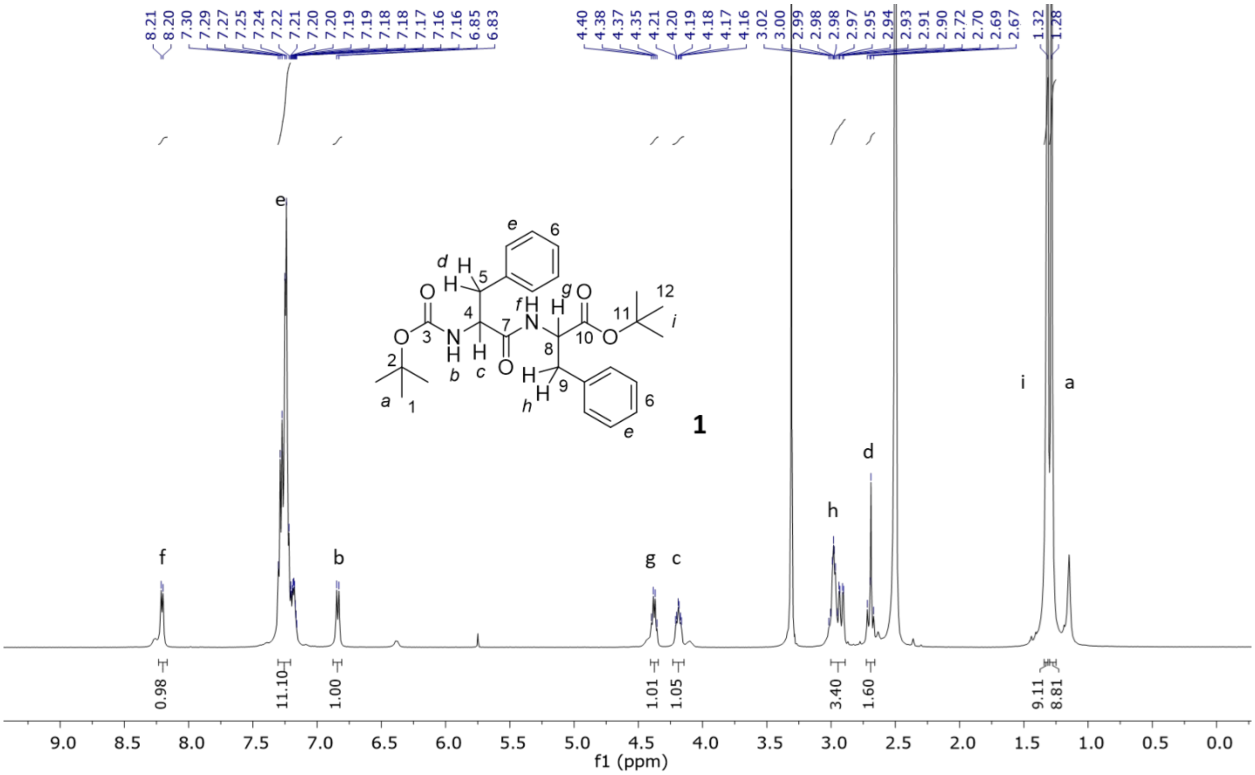

Figure S1. ^{1}H NMR (500 MHz, d_6-DMSO) spectrum of Boc-Phe-Phe-O*t*Bu **1**.

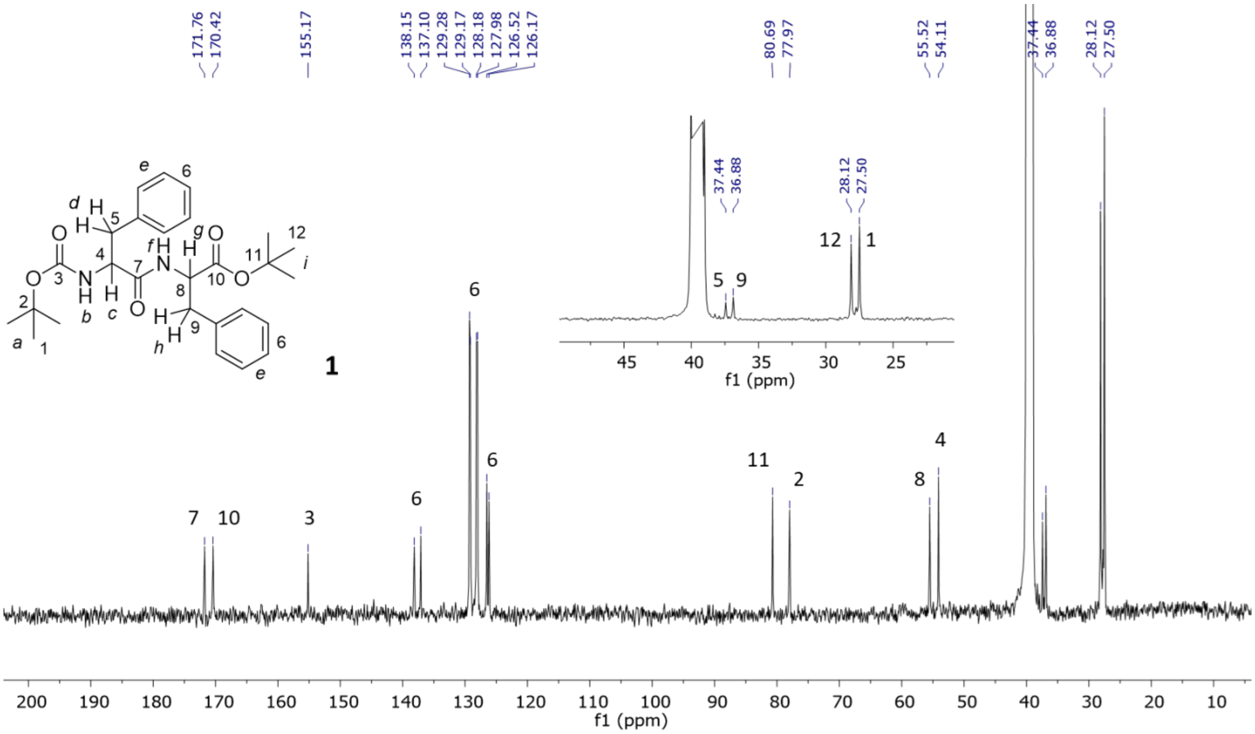

Figure S2. ^{13}C NMR (126 MHz, d_6-DMSO) spectrum of Boc-Phe-Phe-O*t*Bu **1**.

1.1.2. Boc-Phe-Phe-Phe-O*t*Bu 2

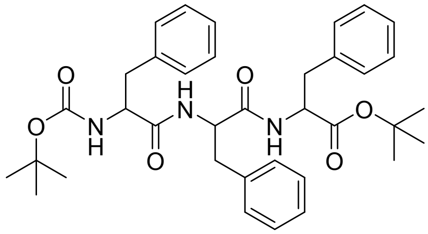

Yellow powder (74 % yield). **Melting point**: 78-83 ˚C.

^{1}H NMR (500 MHz, $CDCl_3$) δ 7.31 – 6.96 (m, 15H), 6.44 (d, *J* = 7.2 Hz, 1H), 6.27 – 6.16 (m, 1H), 4.85 (s, 1H), 4.56 (dq, *J* = 14.9, 7.4, 6.8 Hz, 2H), 4.35 – 4.25 (m, 1H), 3.06 – 2.89 (m, 6H), 1.36 (s, 9H), 1.34 (s, 9H).

^{13}C NMR (126 MHz, $CDCl_3$) δ 171.0, 169.8, 169.7, 155.3, 136.5, 136.2, 136.0, 129.5, 129.3, 128.7, 128.6, 128.4, 127.0, 127.0, 82.3, 80.3, 55.7, 54.4, 53.9, 38.1, 28.2, 27.9.

^{1}H NMR (500 MHz, d_6-DMSO) δ 8.46 (d, *J* = 7.5 Hz, 0.7H), 8.33 (d, *J* = 7.5 Hz, 0.3H), 8.03 (d, *J* = 8.6 Hz, 0.3H), 7.90 (d, *J* = 8.4 Hz, 0.7H), 7.34 – 7.12 (m, 15H), 6.84 (d, *J* = 8.9 Hz, 0.7H), 4.62 (td, *J* = 8.8, 4.4 Hz, 0.7H), 4.58 – 4.52 (m, 0.3H), 4.38 (q, *J* = 7.5 Hz, 1H), 4.10 (td, *J* = 9.7, 3.9 Hz, 0.7H), 4.02 (s, 0.3H), 3.07 – 2.94 (m, 3H), 2.85 – 2.76 (m, 2H), 2.65 – 2.57 (m, 1H), 1.31 (s, 9H), 1.27 (s, 9H). Note: presence of a rotamer (N-H peaks at 8.33 ppm and 8.03 ppm and C-H peak at 4.58 – 4.52 ppm). Peaks corresponding to the rotamer in DMSO vanishes in $CDCl_3$ (Figure S3) and higher temperature measurements (Figure S6).

^{13}C NMR (126 MHz, d_6-DMSO) δ 171.7, 171.4, 170.8, 155.5, 138.6, 137.9, 137.5, 129.8, 129.6, 128.7, 128.4, 127.0, 126.7, 126.6, 81.2, 78.6, 56.3, 54.7, 53.7, 38.4, 38.1, 37.4, 28.6, 28.0.

HR-MS: *m/z* for $C_{36}H_{45}N_3O_6$ $[M + Na]^+$ calculated 638.3100, found 638.3192.

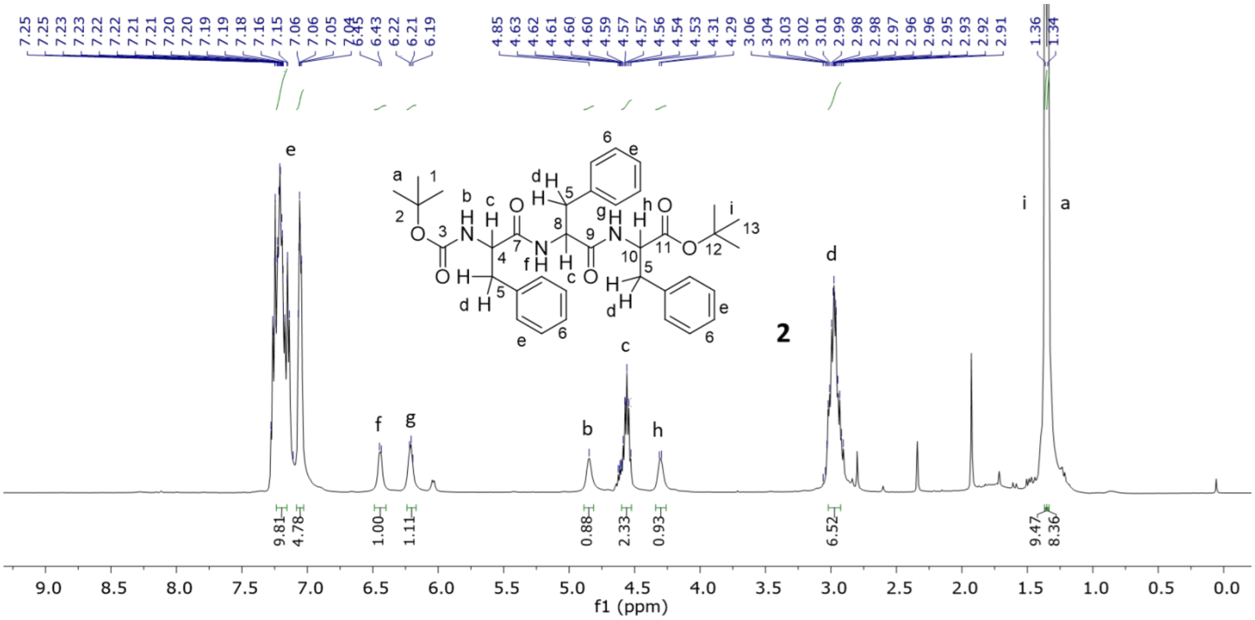

Figure S3. ^{1}H NMR (500 MHz, $CDCl_3$) spectrum of Boc-Phe-Phe-Phe-O*t*Bu **2**.

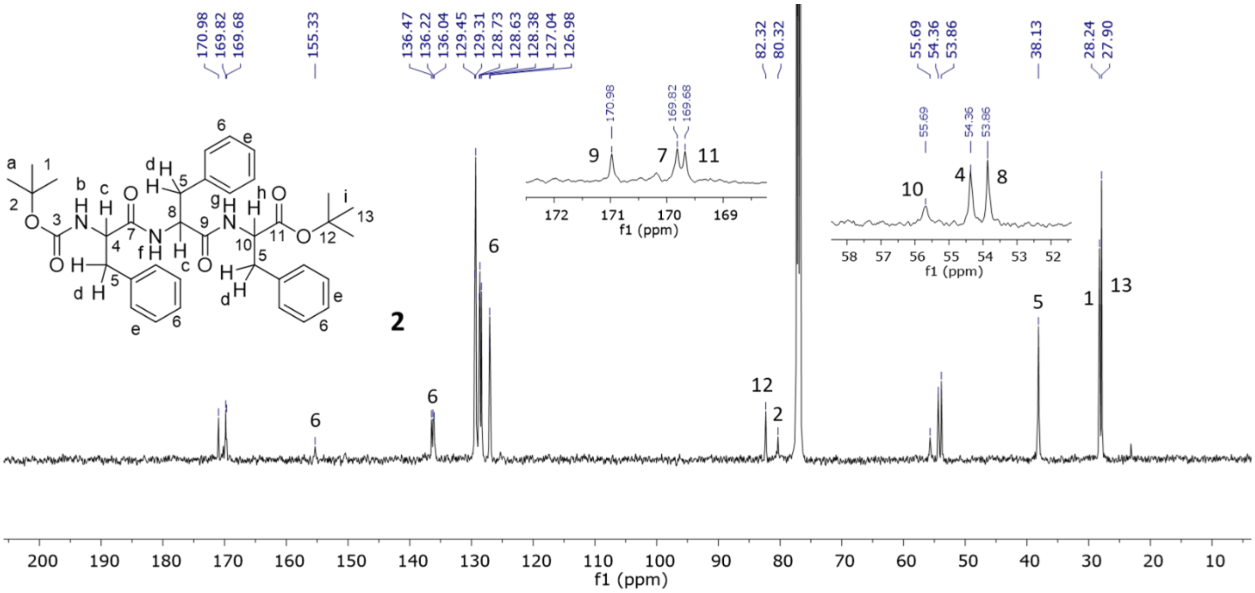

Figure S4. ^{13}C NMR (126 MHz, $CDCl_3$) spectrum of Boc-Phe-Phe-Phe-O*t*Bu **2**.

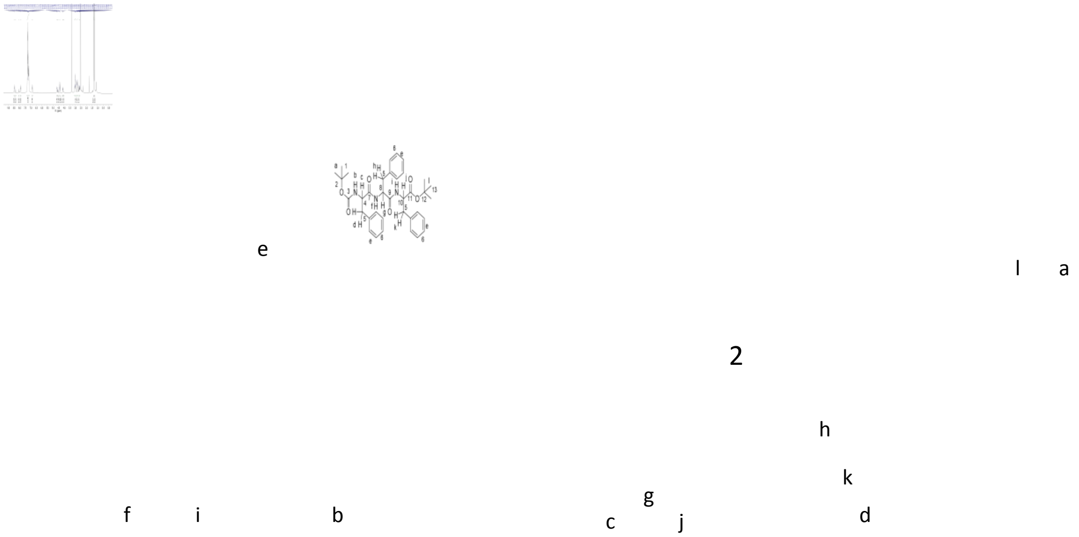

Figure S5. ^{1}H NMR (500 MHz, d_6-DMSO) spectrum of Boc-Phe-Phe-Phe-O*t*Bu **2**. Presence of a rotamer.

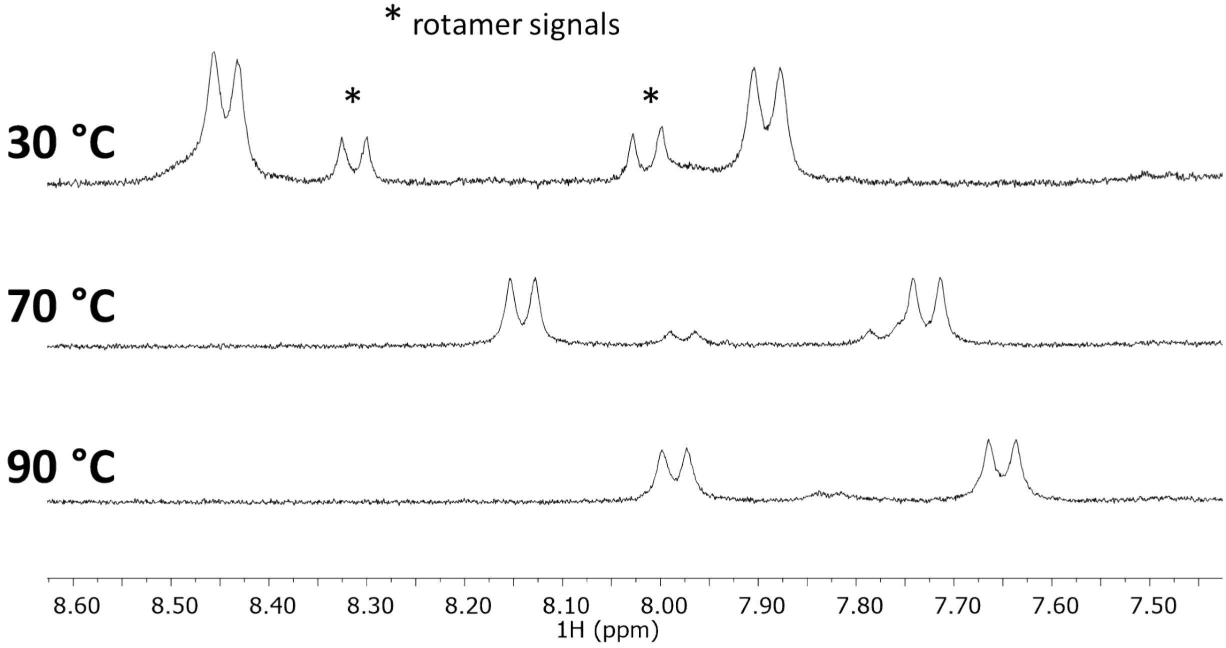

Figure S6. ^{1}H NMR (500 MHz, d_6-DMSO) spectrum of Boc-Phe-Phe-Phe-O*t*Bu **2** magnified in the NH amide region showing the rotamer signal at R.T. (30 ˚C, top row). Temperature increase to 70 ˚C (middle row) and 90 ˚C (bottom row) showing the disappearance of the rotamer signals.

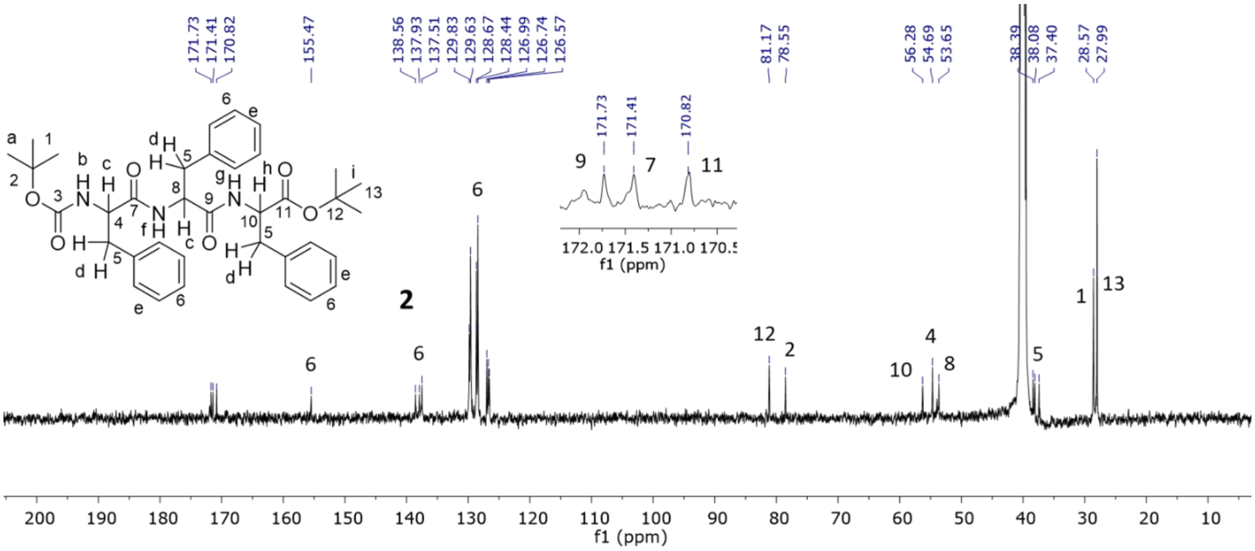

Figure S7. ^{13}C NMR (126 MHz, d_6-DMSO) spectrum of Boc-Phe-Phe-Phe-O*t*Bu **2**.

1.1.3. Boc-Leu-Phe-O*t*Bu 3

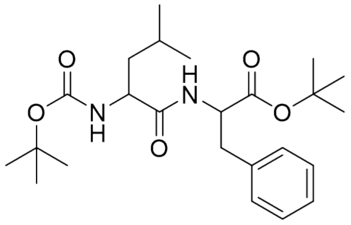

Yellow powder (63 % yield). **Melting point**: 123-126 ˚C.

^{1}H NMR (500 MHz, d_6-DMSO) δ 7.99 (d, *J* = 7.6 Hz, 1H), 7.23 (dd, *J* = 22.5, 7.1 Hz, 5H), 6.80 (d, *J* = 8.7 Hz, 1H), 4.35 (q, *J* = 7.5 Hz, 1H), 3.97 (dt, *J* = 14.8, 7.0 Hz, 1H), 2.94 (qd, *J* = 13.6, 6.9 Hz, 2H), 1.59 – 1.51 (m, 1H), 1.37 (s, 9H), 1.34 (m, 2H), 1.31 (s, 9H), 0.84 (dd, *J* = 14.2, 6.6 Hz, 6H). Note: 1.34 ppm peak is hidden within 1.37 ppm and 1.31 ppm peaks.

^{13}C NMR (126 MHz, d_6-DMSO) δ 172.4, 170.3, 155.1, 137.1, 129.2, 128.1, 126.4, 80.6, 77.9, 53.8, 52.6, 40.8, 36.8, 28.1, 27.5, 24.1, 22.9, 21.5.

HR-MS: *m/z* for $C_{24}H_{38}N_2O_5$ [M + Na]$^+$ calculated 457.2600, found 457.2650.

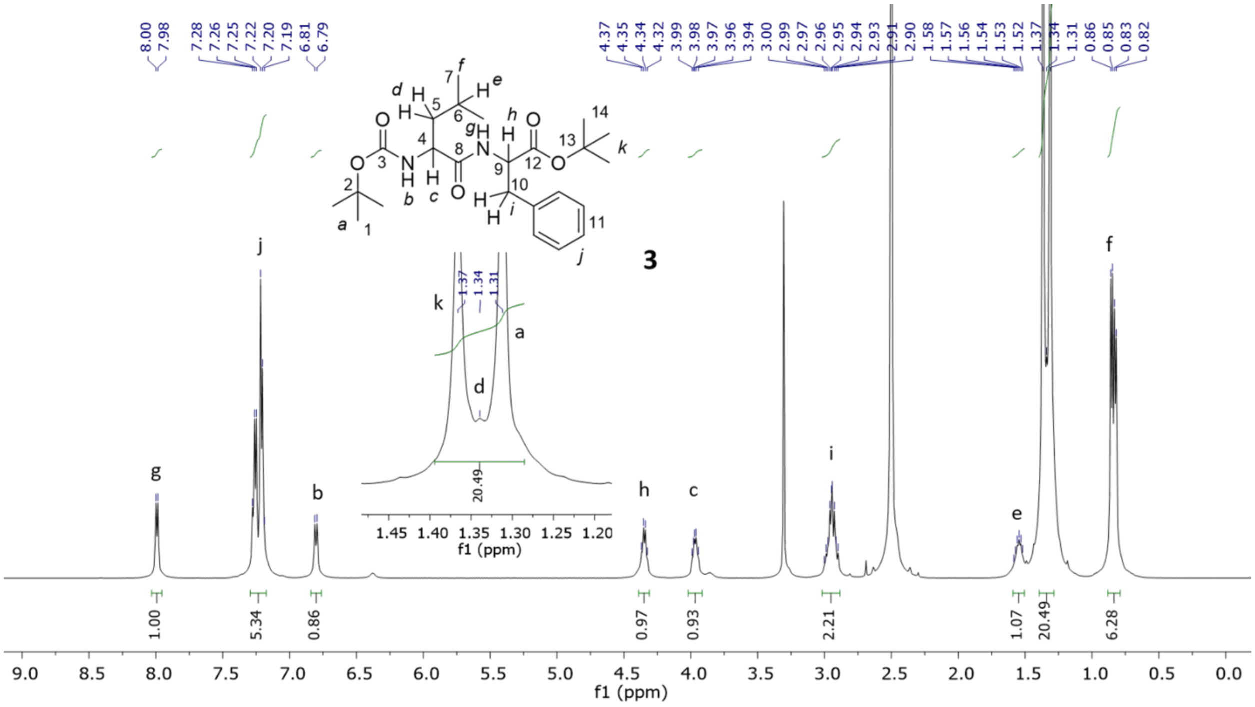

Figure S8. ^{1}H NMR (500 MHz, d_6-DMSO) spectrum of Boc-Leu-Phe-O*t*Bu **3**.

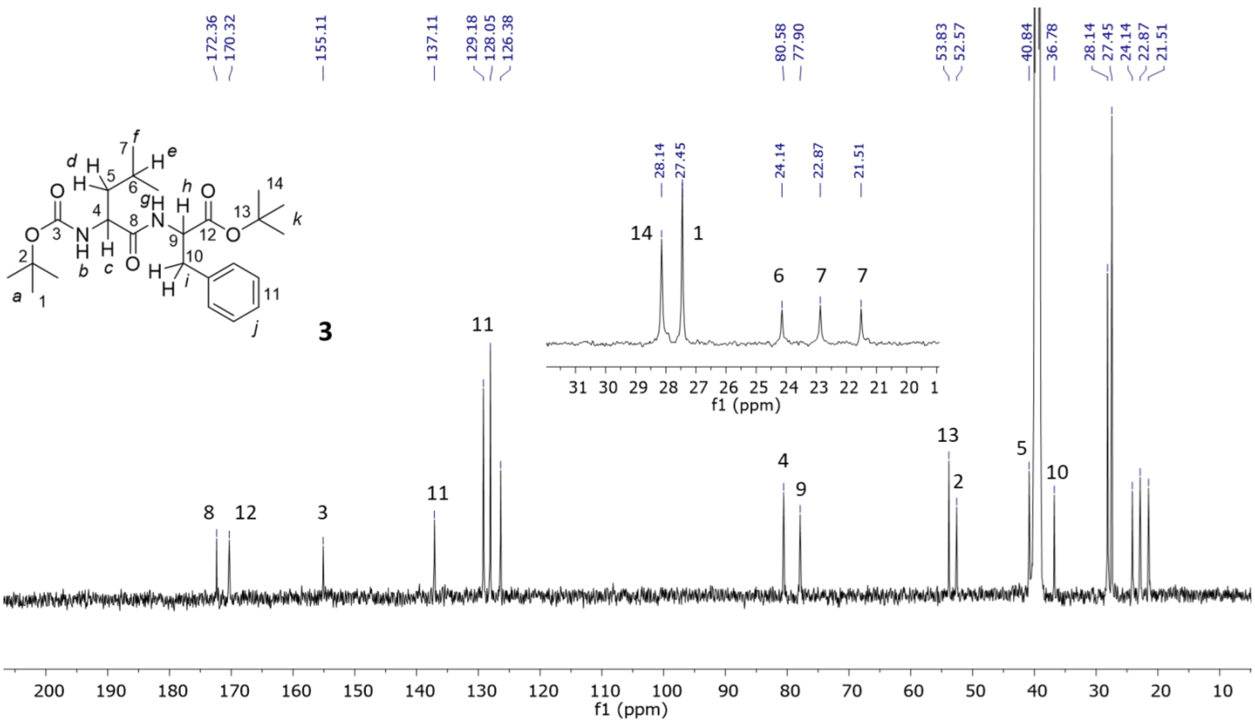

Figure S9. ^{13}C NMR (126 MHz, d_6-DMSO) spectrum of Boc-Leu-Phe-O*t*Bu **3**.

1.1.4. Boc-Ile-Phe-O*t*Bu 4

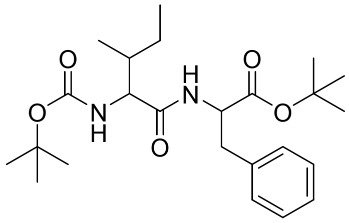

Yellow powder (54 % yield). **Melting point**: 106-110 ˚C.

^{1}H NMR (500 MHz, *d*$_6$-DMSO) δ 8.11 (d, *J* = 7.7 Hz, 1H), 7.22 (dq, *J* = 14.9, 7.6 Hz, 5H), 6.56 (d, *J* = 9.3 Hz, 1H), 4.37 (d, *J* = 7.4 Hz, 1H), 3.82 (t, *J* = 8.4 Hz, 1H), 2.91 (qd, *J* = 13.8, 7.4 Hz, 2H), 1.64 – 1.56 (m, 1H), 1.36 (s, 9H), 1.32 (m, 1H), 1.29 (s, 9H), 1.02 (dt, *J* = 14.6, 7.7 Hz, 1H), 0.80 – 0.69 (m, 6H). Note: 1.32 ppm peak is hidden within 1.36 ppm and 1.29 ppm peaks.

^{13}C NMR (126 MHz, *d*$_6$-DMSO) δ 171.2, 170.4, 155.1, 137.1, 129.1, 128.1, 126.4, 80.5, 77.9, 58.5, 53.9, 36.9, 36.7, 28.1, 27.5, 24.2, 15.2, 10.9.

HR-MS: *m/z* for $C_{24}H_{38}N_2O_5$ $[M + Na]^+$ calculated 457.2600, found 457.2682.

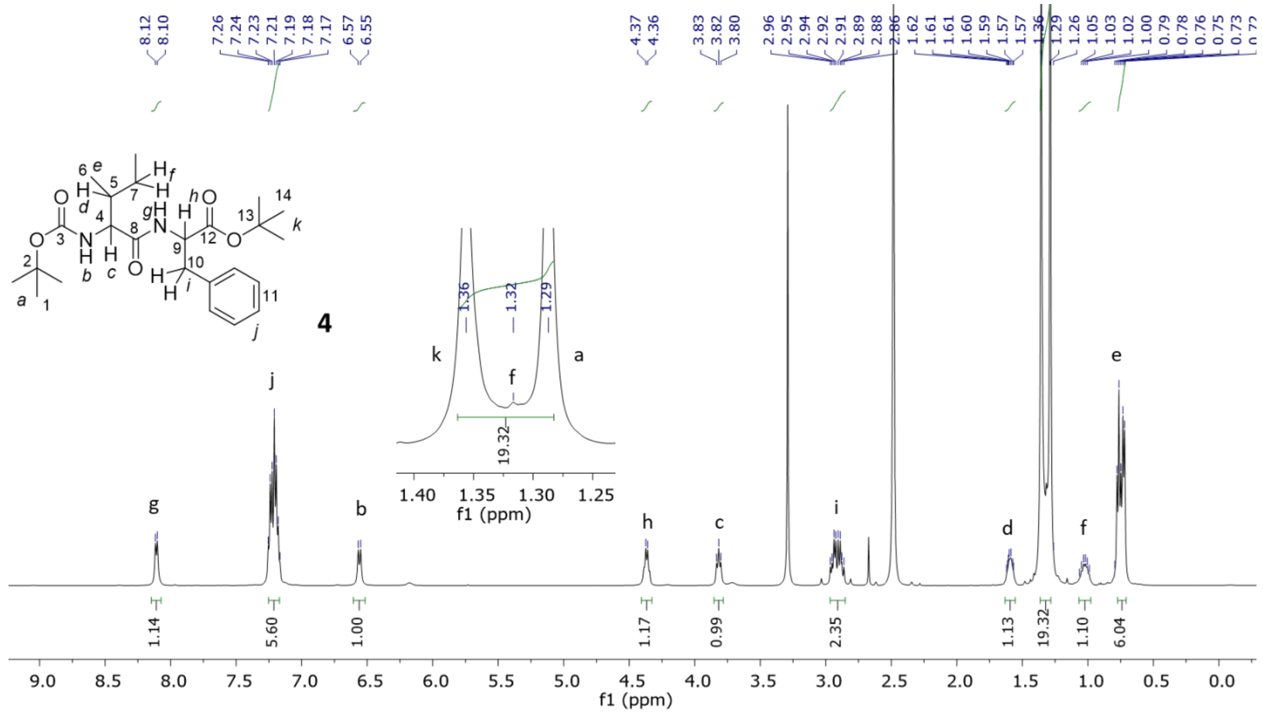

Figure S10. ^{1}H NMR (500 MHz, d_6-DMSO) spectrum of Boc-Ile-Phe-O*t*Bu **4**.

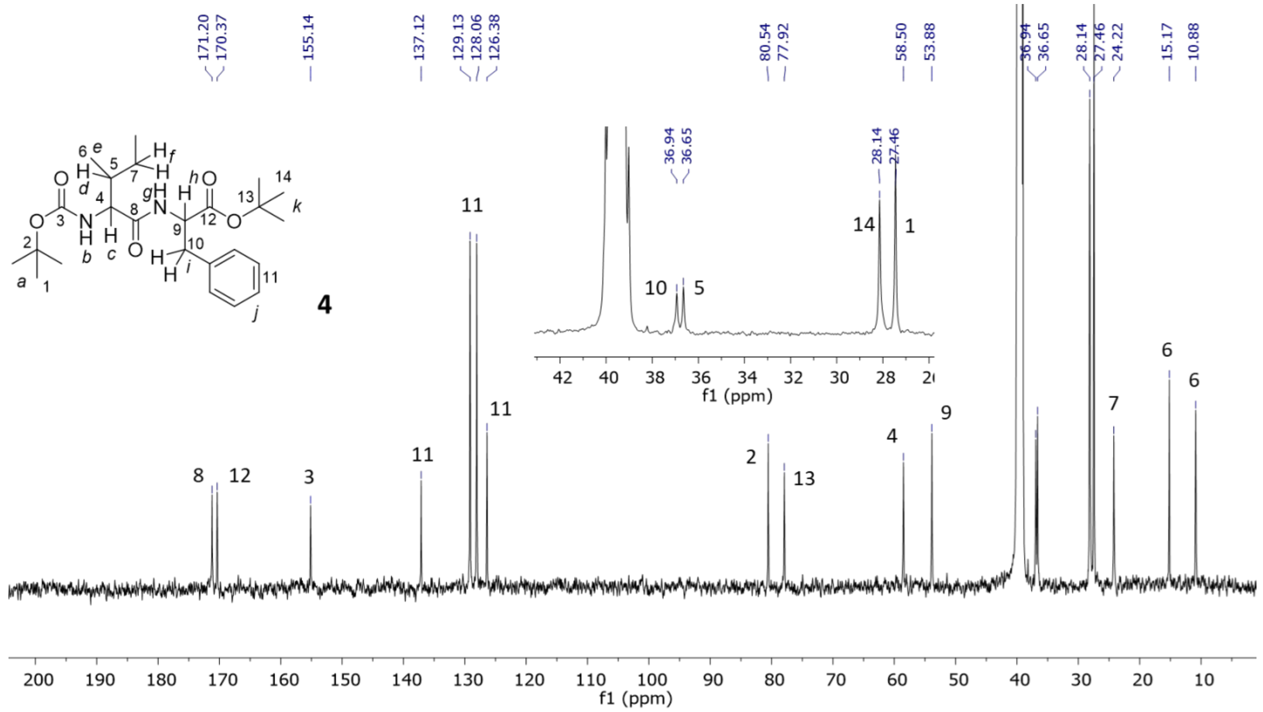

Figure S11. ^{13}C NMR (126 MHz, d_6-DMSO) spectrum of Boc-Ile-Phe-O*t*Bu **4**.

1.1.5. Boc-Val-Phe-O*t*Bu 5

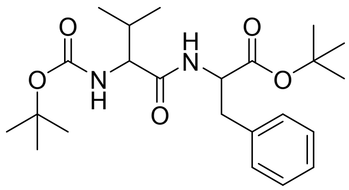

Yellow powder (60 % yield). **Melting point**: 110-113 ˚C.

^{1}H NMR (500 MHz, d_6-DMSO) δ 8.13 (d, J = 7.7 Hz, 1H), 7.23 (dp, J = 21.7, 7.4 Hz, 5H), 6.54 (d, J = 9.2 Hz, 1H), 4.38 (q, J = 7.5 Hz, 1H), 3.84 – 3.78 (m, 1H), 2.93 (qd, J = 13.8, 7.4 Hz, 2H), 1.87 (h, J = 6.9 Hz, 1H), 1.30 (s, 9H), 1.37 (s, 9H), 0.78 (dd, J = 6.8, 3.2 Hz, 6H).

^{13}C NMR (126 MHz, d_6-DMSO) δ 171.2, 170.4, 155.3, 137.1, 129.1, 128.1, 126.4, 80.6, 77.9, 59.4, 53.9, 36.9, 30.5, 28.1, 27.4, 19.1, 18.0.

HR-MS: m/z for $C_{23}H_{36}N_2O_5$ $[M + Na]^+$ calculated 443.2400, found 443.2528.

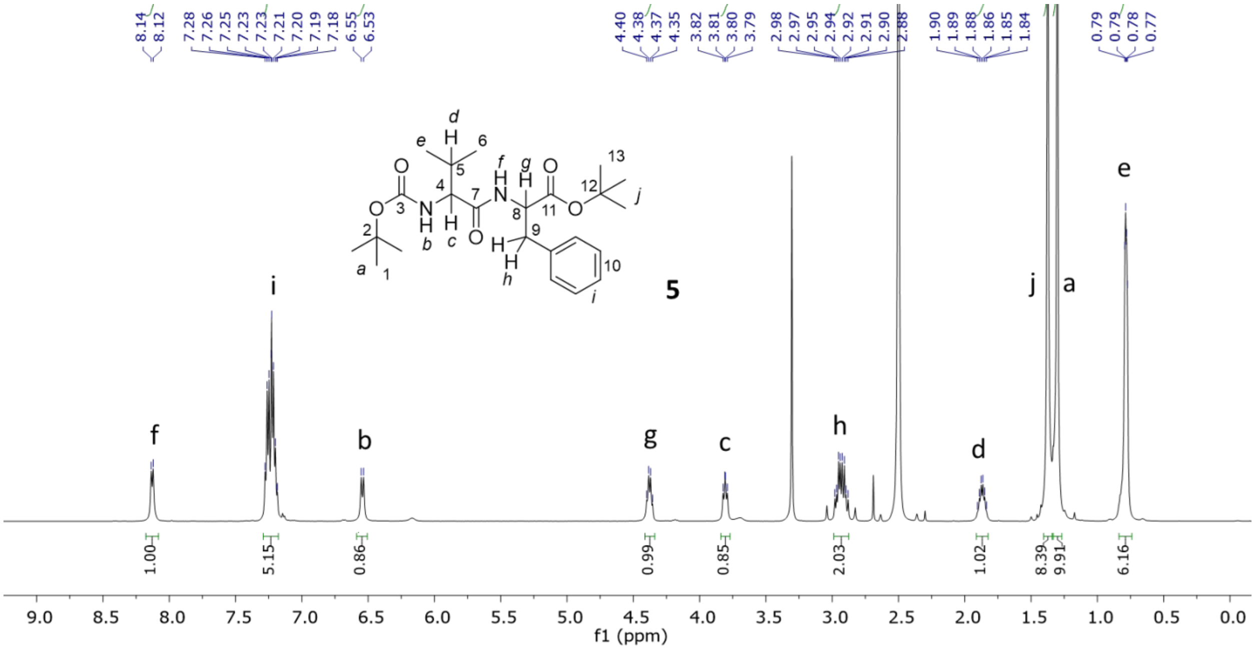

Figure S12. ^{1}H NMR (500 MHz, d_6-DMSO) spectrum of Boc-Val-Phe-O*t*Bu **5**.

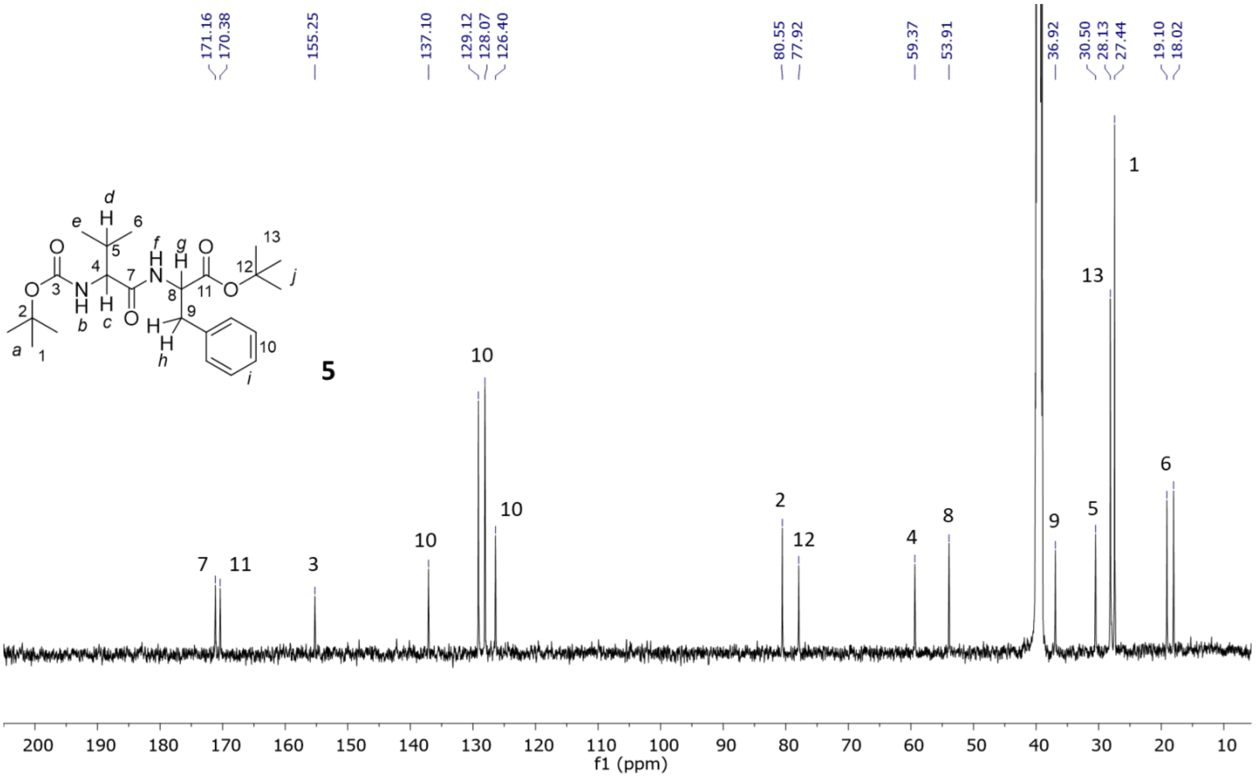

Figure S13. ^{13}C NMR (126 MHz, d_6-DMSO) spectrum of Boc-Val-Phe-O*t*Bu **5**.

1.1.6. Boc-Ala-Phe-O*t*Bu 6

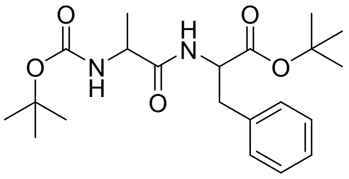

Yellow powder (54 % yield). **Melting point**: 44-47 ˚C.

^{1}H NMR (500 MHz, d_6-DMSO) δ 7.96 (d, J = 7.6 Hz, 1H), 7.22 (dd, J = 24.0, 7.3 Hz, 5H), 6.83 (d, J = 7.9 Hz, 1H), 4.31 (q, J = 7.3 Hz, 1H), 3.96 (p, J = 7.3 Hz, 1H), 2.92 (h, J = 7.6 Hz, 2H), 1.35 (s, 9H), 1.29 (s, 9H), 1.12 (d, J = 7.2 Hz, 3H).

^{13}C NMR (126 MHz, d_6-DMSO) δ 172.6, 170.3, 154.9, 137.1, 129.2, 128.1, 126.4, 80.6, 78.0, 53.9, 49.4, 36.8, 28.2, 27.5, 18.2.

HR-MS: m/z for $C_{21}H_{32}N_2O_5$ $[M + Na]^+$ calculated 415.2100, found 415.2215.

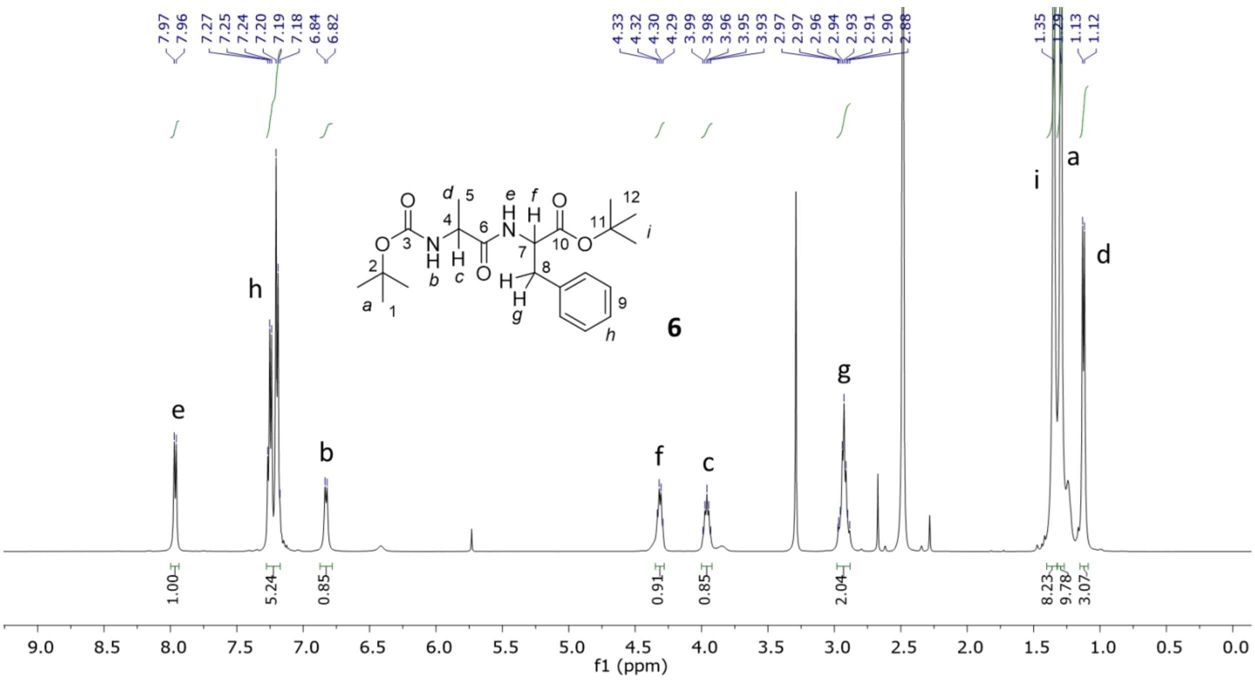

Figure S14. ^{1}H NMR (500 MHz, d_6-DMSO) spectrum of Boc-Ala-Phe-O*t*Bu **6**.

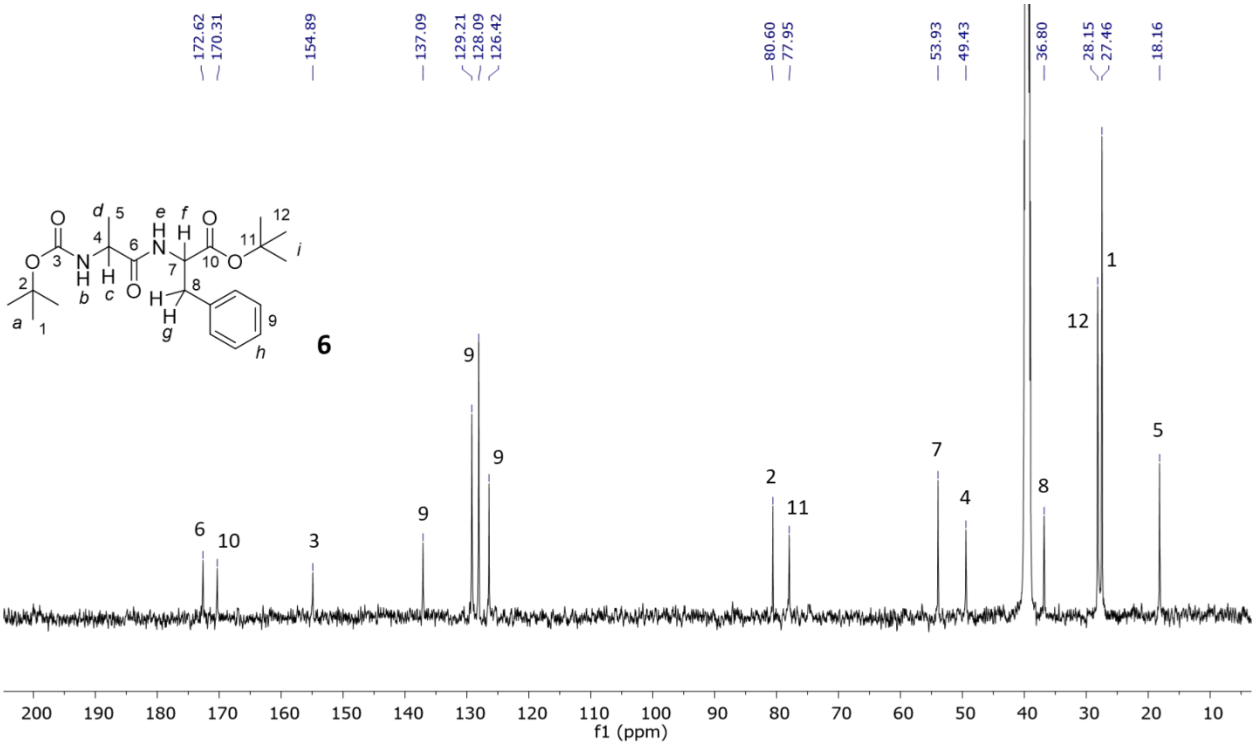

Figure S15. ^{13}C NMR (126 MHz, d_6-DMSO) spectrum of Boc-Ala-Phe-O*t*Bu **6**.

1.1.7. Boc-Gly-Phe-O*t*Bu 7

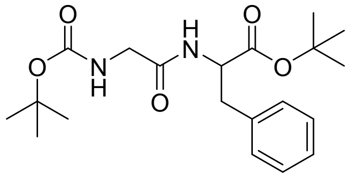

Yellow oil (66 % yield).

^{1}**H NMR** (500 MHz, d_6-DMSO) δ 8.03 (d, *J* = 7.8 Hz, 1H), 7.24 (dt, *J* = 31.3, 7.4 Hz, 5H), 6.91 (t, *J* = 6.2 Hz, 1H), 4.37 (q, *J* = 7.4 Hz, 1H), 3.57 – 3.51 (m, 1H), 2.98 – 2.88 (m, 2H), 1.37 (s, 9H), 1.31 (s, 9H).

^{13}**C NMR** (126 MHz, d_6-DMSO) δ 170.4, 169.3, 155.7, 137.0, 129.2, 128.1, 126.5, 80.7, 78.0, 53.9, 42.9, 37.0, 28.1, 27.5.

HR-MS: *m/z* for $C_{20}H_{30}N_2O_5$ $[M + Na]^+$ calculated 401.2000, found 401.2068.

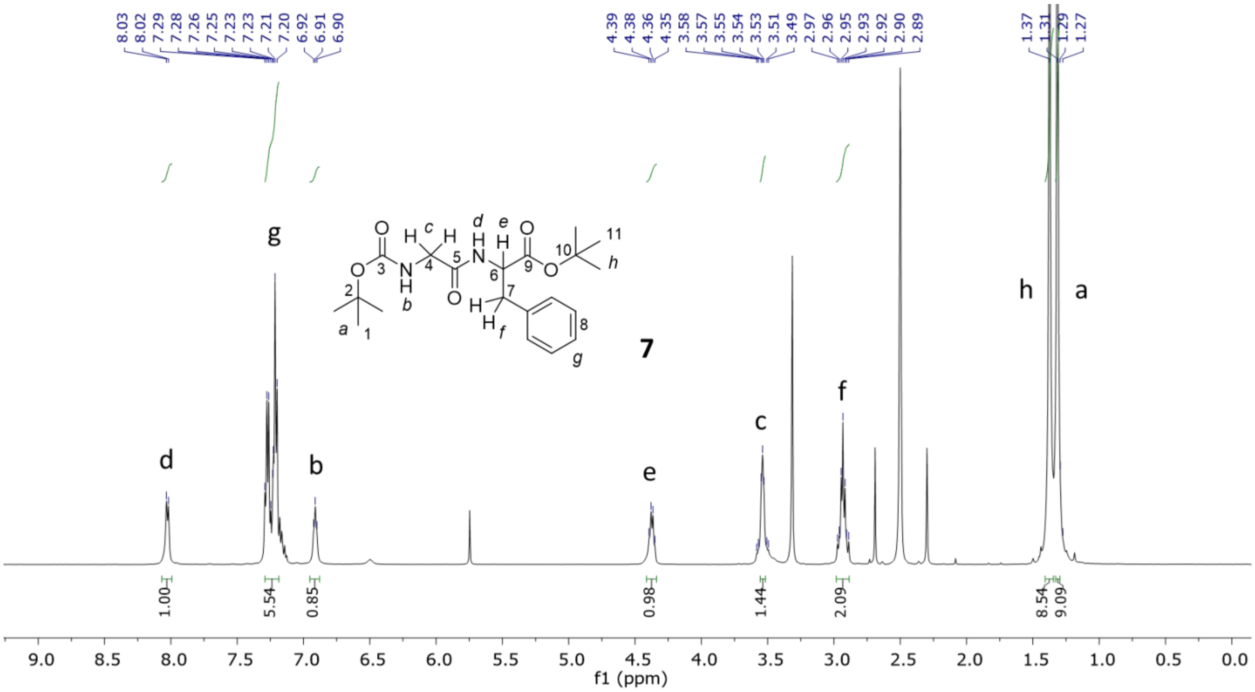

Figure S16. ^{1}H NMR (500 MHz, d_6-DMSO) spectrum of Boc-Gly-Phe-O*t*Bu **7**.

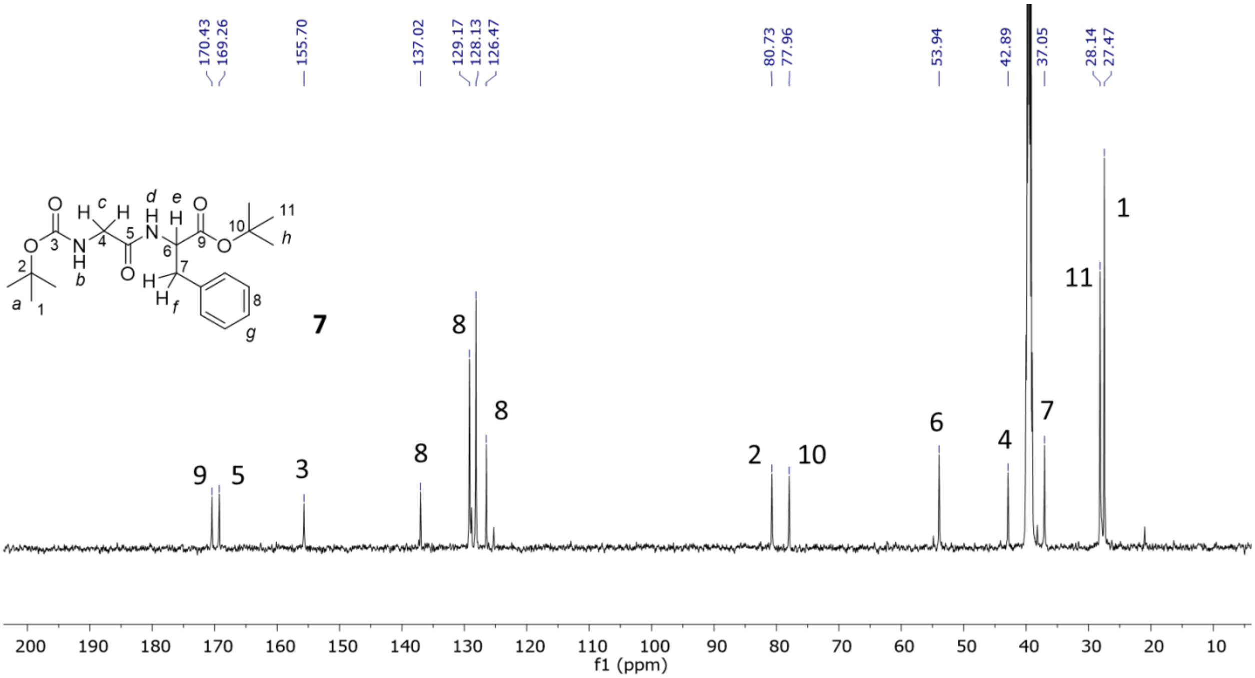

Figure S17. ^{13}C NMR (126 MHz, d_6-DMSO) spectrum of Boc-Gly-Phe-O*t*Bu **7**.

1.1.8. Boc-Tyr-Phe-O*t*Bu 8

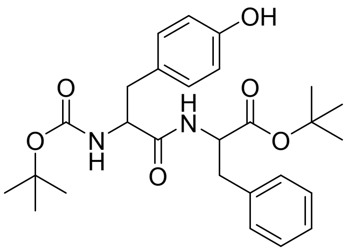

Yellow powder (63 % yield). **Melting point**: 77-84 ˚C.

^{1}H NMR (500 MHz, d_6-DMSO) δ 9.12 (s, 1H), 8.15 (d, *J* = 7.5 Hz, 1H), 7.29 – 7.21 (m, 6H), 7.01 (d, *J* = 8.1 Hz, 2H), 6.73 (d, *J* = 8.7 Hz, 1H), 6.63 (d, *J* = 8.0 Hz, 2H), 4.36 (q, *J* = 7.3 Hz, 1H), 4.09 (td, *J* = 9.5, 4.0 Hz, 1H), 3.01 – 2.92 (m, 2H), 2.80 (dd, *J* = 14.0, 4.1 Hz, 1H), 2.58 (dd, *J* = 13.9, 10.3 Hz, 1H), 1.31 (s, 9H), 1.30 (s, 9H).

^{13}C NMR (126 MHz, d_6-DMSO) δ 172.3, 170.9, 156.2, 155.6, 137.6, 130.5, 129.7, 129.4, 128.6, 127.0, 125.8, 115.3, 81.2, 78.4, 56.3, 54.5, 37.4, 37.1, 28.6, 28.0.

HR-MS: *m/z* for $C_{27}H_{36}N_2O_6$ $[M + Na]^+$ calculated 507.2400, found 507.2461.

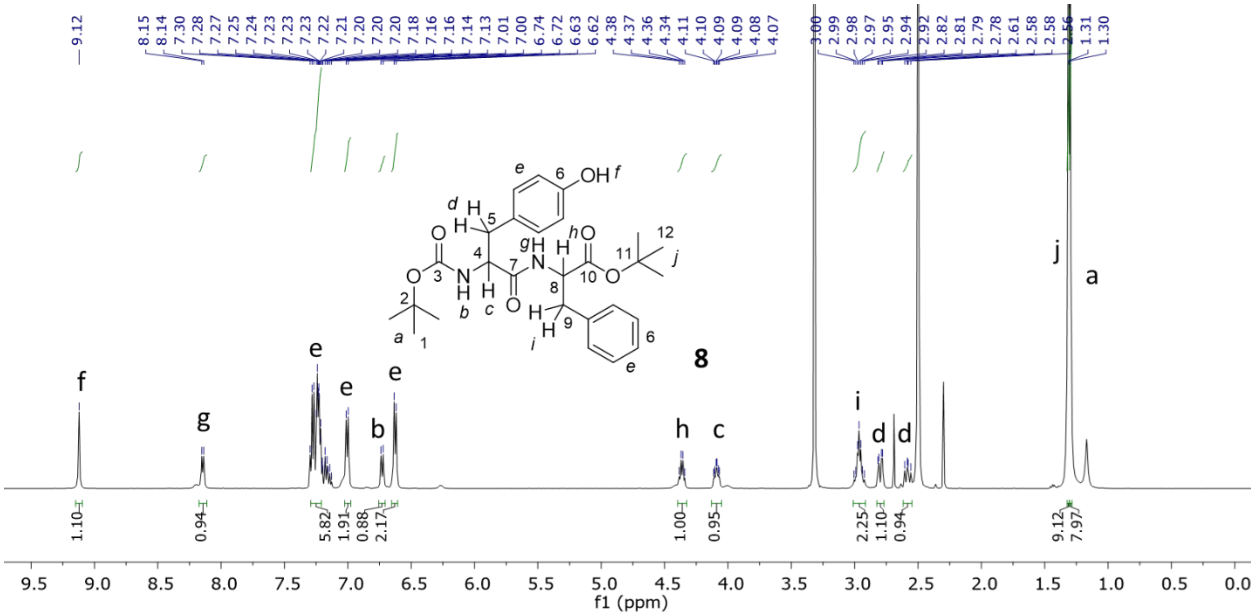

Figure S18. ^{1}H NMR (500 MHz, d_6-DMSO) spectrum of Boc-Tyr-Phe-O*t*Bu **8**.

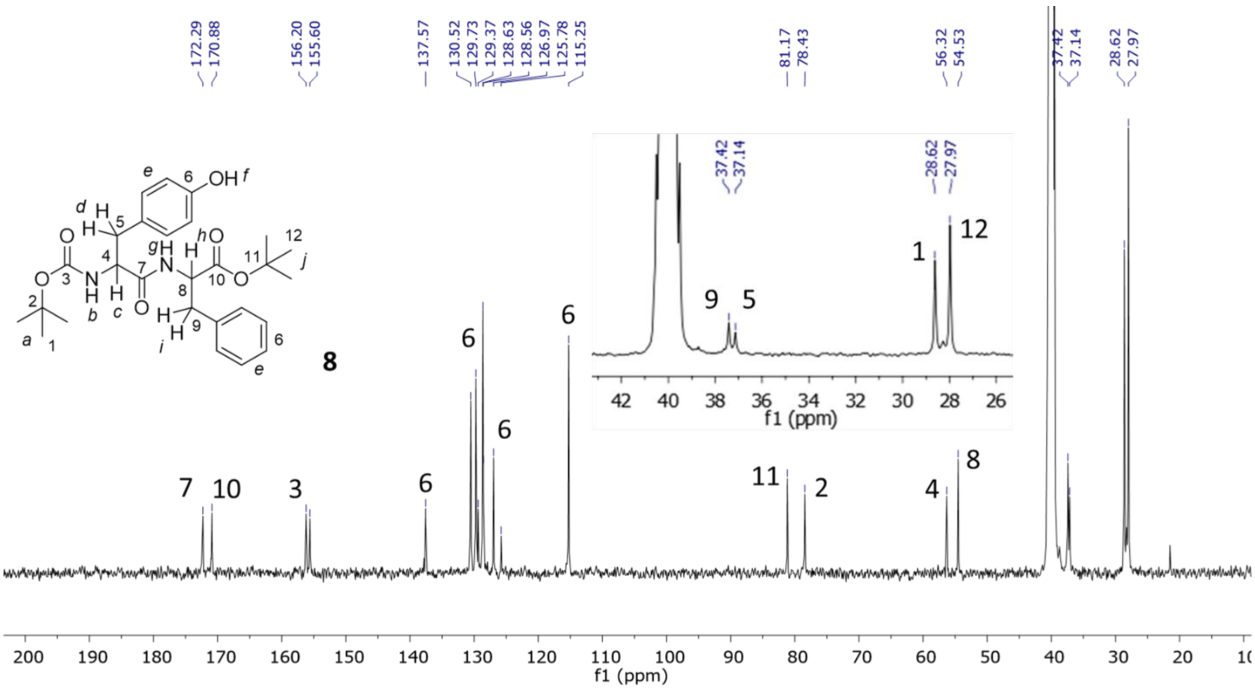

Figure S19. ^{13}C NMR (126 MHz, d_6-DMSO) spectrum of Boc-Tyr-Phe-O*t*Bu **8**.

1.2. Synthesis of monoprotected compounds 1a-3a

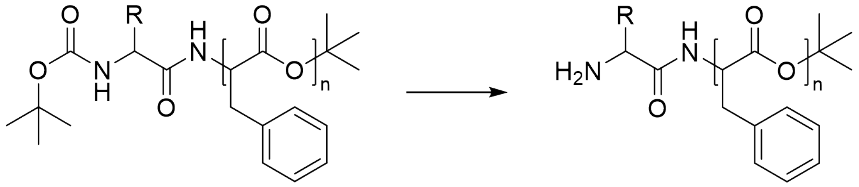

General protocol: Boc-protected precursor (1.0 equivalent) was suspended in *t*BuOAc at a concentration of 0.2 M. Concentrated H_2SO_4 (5.0 equivalent) was then added dropwise at R.T. After 1 hour, the reaction mixture was neutralised with a saturated solution of $NaHCO_3$ and extracted with ethyl acetate. The organic phase was then dried with $MgSO_4$ and evaporated under vacuum, yielding the desired compounds.

1.2.1. Phe-Phe-O*t*Bu 1a

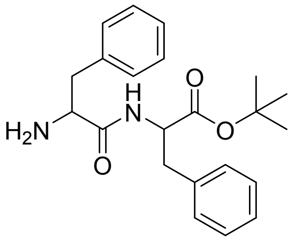

Yellow powder (95 % yield). **Melting point**: 63-67 °C.

^{1}H NMR (500 MHz, *d*$_6$-DMSO) δ 8.16 (d, *J* = 8.0 Hz, 1H), 7.30 – 7.12 (m, 10H), 4.43 (q, *J* = 7.2 Hz, 1H), 3.40 (dd, *J* = 8.4, 4.6 Hz, 1H), 2.92 (dd, *J* = 22.4, 5.8 Hz, 3H), 2.59 – 2.55 (m, 1H), 1.65 (s, 2H), 1.33 (s, 9H).

^{13}C NMR (126 MHz, *d*$_6$-DMSO) δ 174.2, 170.6, 138.7, 137.1, 129.5, 129.4, 128.3, 128.2, 126.7, 126.3, 81.0, 55.9, 53.6, 40.9, 37.4, 27.7.

HR-MS: *m/z* for $C_{22}H_{28}N_2O_3$ $[M + H]^+$ calculated 369.2100, found 369.2161.

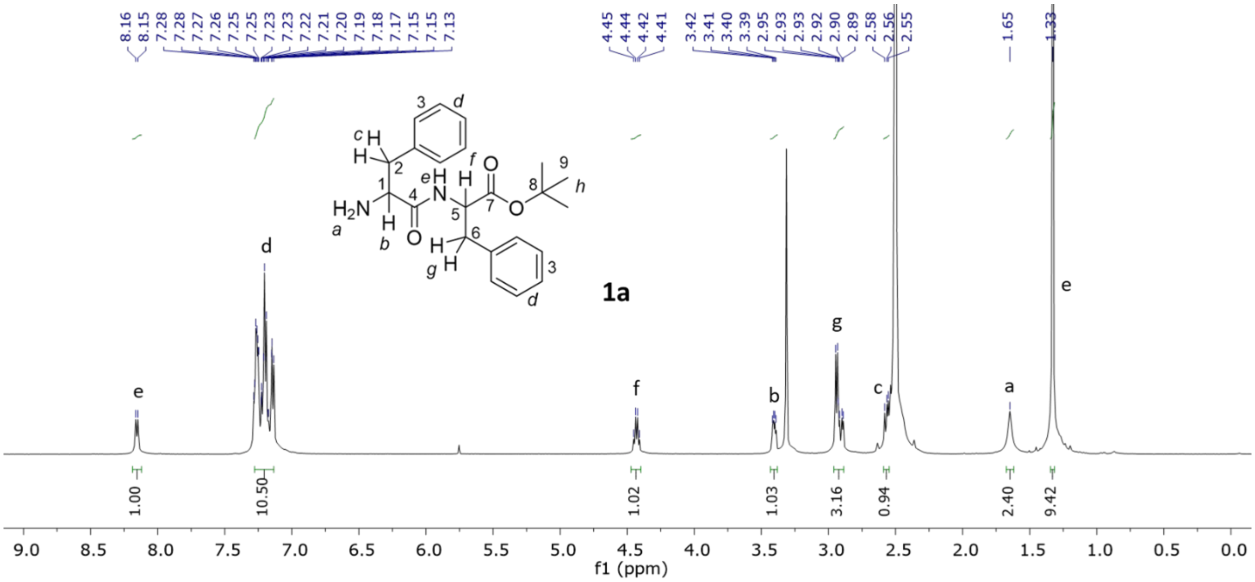

Figure S20. ^{1}H NMR (500 MHz, d_6-DMSO) spectrum of Phe-Phe-O*t*Bu **1a**.

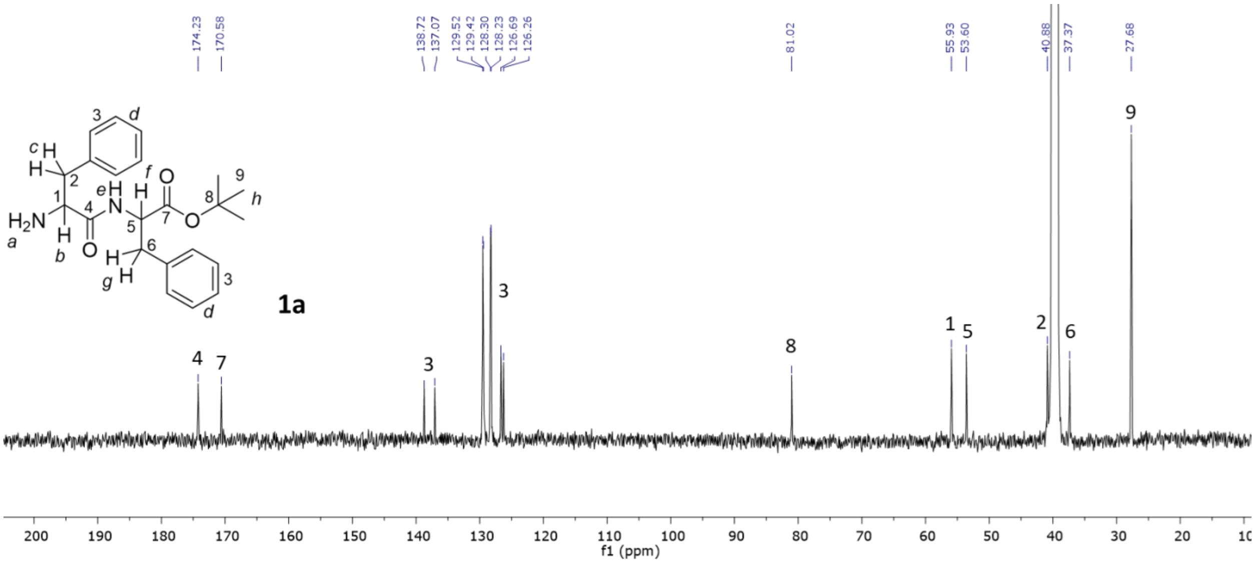

Figure S21. ^{13}C NMR (126 MHz, d_6-DMSO) spectrum of Phe-Phe-O*t*Bu **1a**.

1.2.2. Phe-Phe-Phe-O*t*Bu 2a

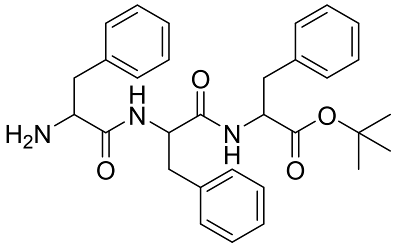

Colourless powder (95 % yield). **Melting point**: 184-187 ˚C.

[1]**H NMR** (500 MHz, d_6-DMSO) δ 8.55 (d, *J* = 7.5 Hz, 1H), 8.40 (s, 1H), 7.31 – 7.16 (m, 15H), 4.64 (td, *J* = 8.6, 4.5 Hz, 1H), 4.40 (q, *J* = 7.5 Hz, 1H), 3.66 (s, 1H), 3.05 – 2.90 (m, 4H), 2.81 (dd, *J* = 13.9, 8.9 Hz, 1H), 2.70 – 2.62 (m, 1H), 1.32 (s, 9H).

[13]**C NMR** (126 MHz, d_6-DMSO) δ 171.2, 170.9, 137.8, 137.5, 129.9, 129.8, 129.7, 128.8, 128.7, 128.5, 127.1, 127.0, 126.8, 81.2, 55.0, 54.7, 53.7, 38.4, 37.4, 28.0.

HR-MS: *m/z* for $C_{31}H_{37}N_3O_4$ $[M + H]^+$ calculated 516.2800, found 516.2876.

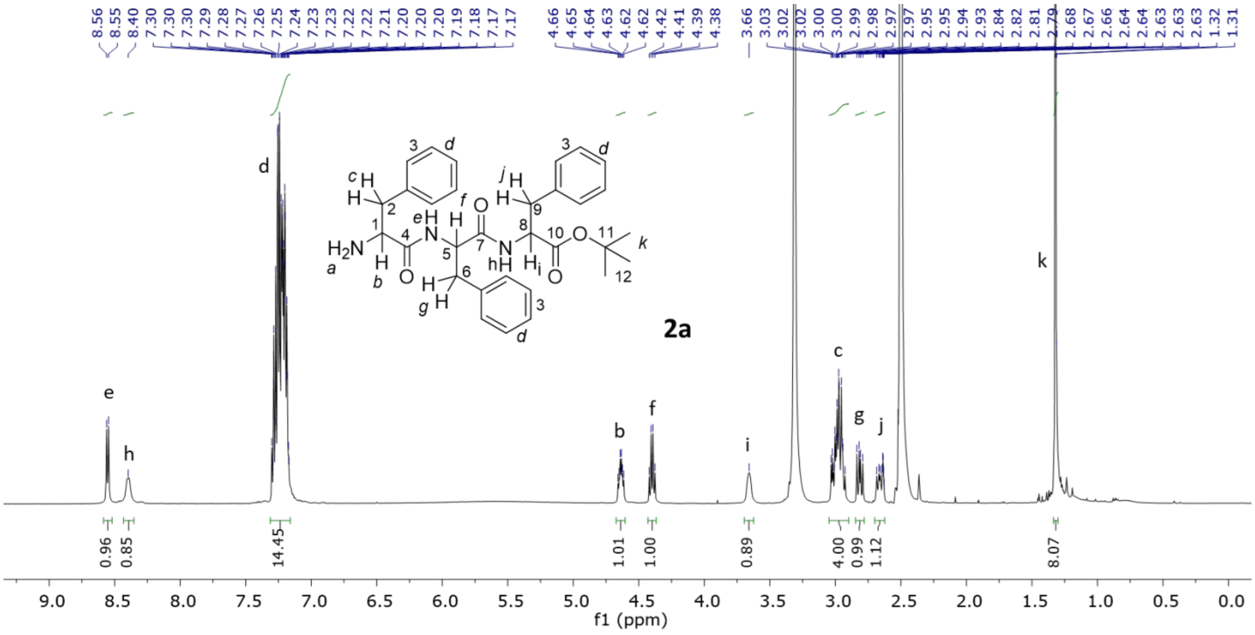

Figure S22. ^{1}H NMR (500 MHz, d_6-DMSO) spectrum of Phe-Phe-Phe-O*t*Bu **2a**.

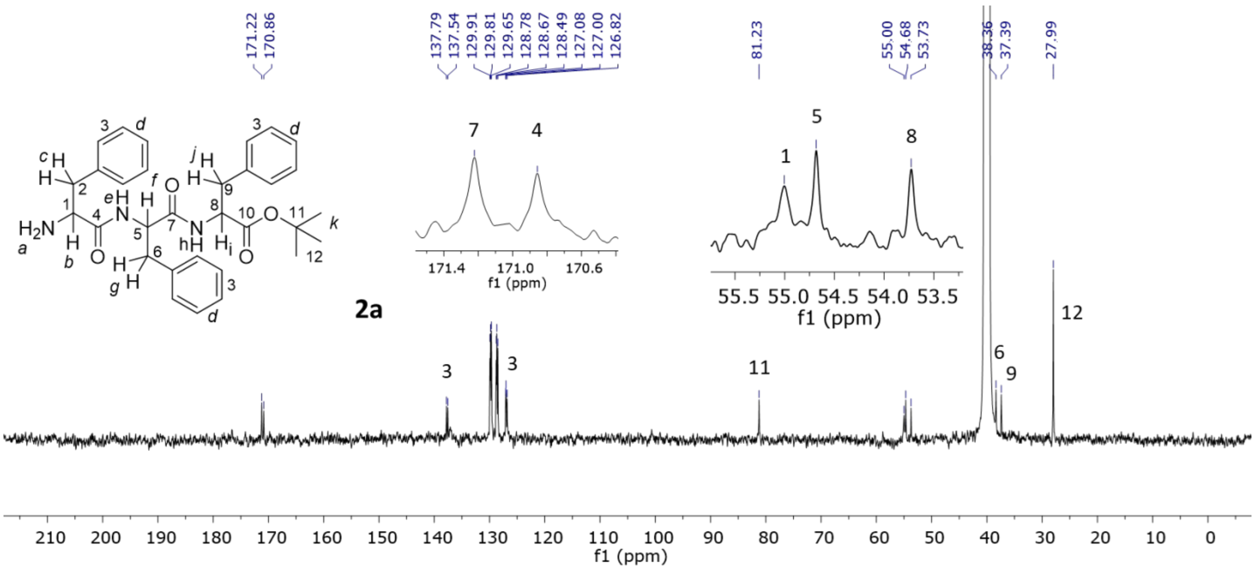

Figure S23. ^{13}C NMR (126 MHz, d_6-DMSO) spectrum of Phe-Phe-Phe-O*t*Bu **2a**.

1.2.3. Leu-Phe-O*t*Bu 3a

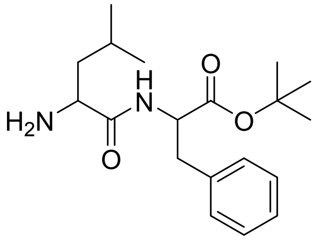

Yellow powder (78 % yield). **Melting point**: 88-93 ˚C.

^{1}H NMR (500 MHz, d_6-DMSO) δ 8.13 (d, J = 8.0 Hz, 1H), 7.30 – 7.17 (m, 5H), 4.41 (q, J = 7.3 Hz, 1H), 3.15 (dd, J = 9.0, 5.3 Hz, 1H), 2.96 (qd, J = 13.7, 7.1 Hz, 2H), 1.82 (d, J = 25.1 Hz, 2H), 1.66 (dq, J = 13.3, 6.8 Hz, 1H), 1.33 (s, 9H), 1.14 (ddd, J = 14.0, 8.9, 5.9 Hz, 1H), 0.84 (dd, J = 19.2, 6.6 Hz, 6H).

^{13}C NMR (126 MHz, d_6-DMSO) δ 175.3, 170.5, 137.0, 129.2, 128.1, 126.5, 80.7, 53.4, 52.8, 44.1, 37.1, 27.5, 24.0, 23.2, 21.8.

HR-MS: *m/z* for $C_{19}H_{30}N_2O_3$ $[M + H]^+$ calculated 335.2300, found 335.2327.

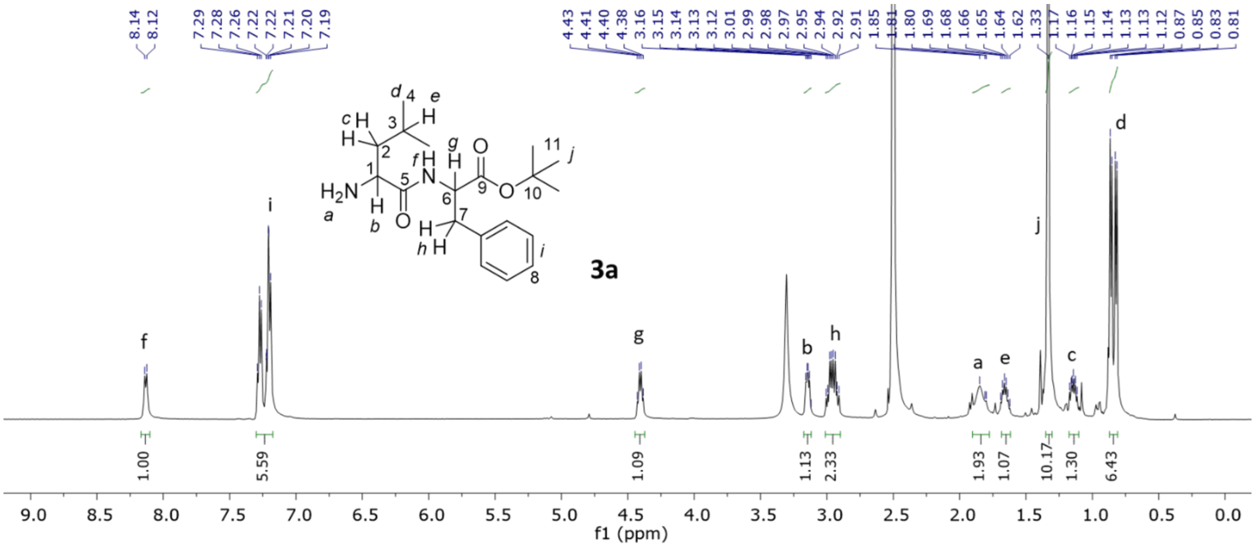

Figure S24. ^{1}H NMR (500 MHz, d_6-DMSO) spectrum of Leu-Phe-O*t*Bu **3a**.

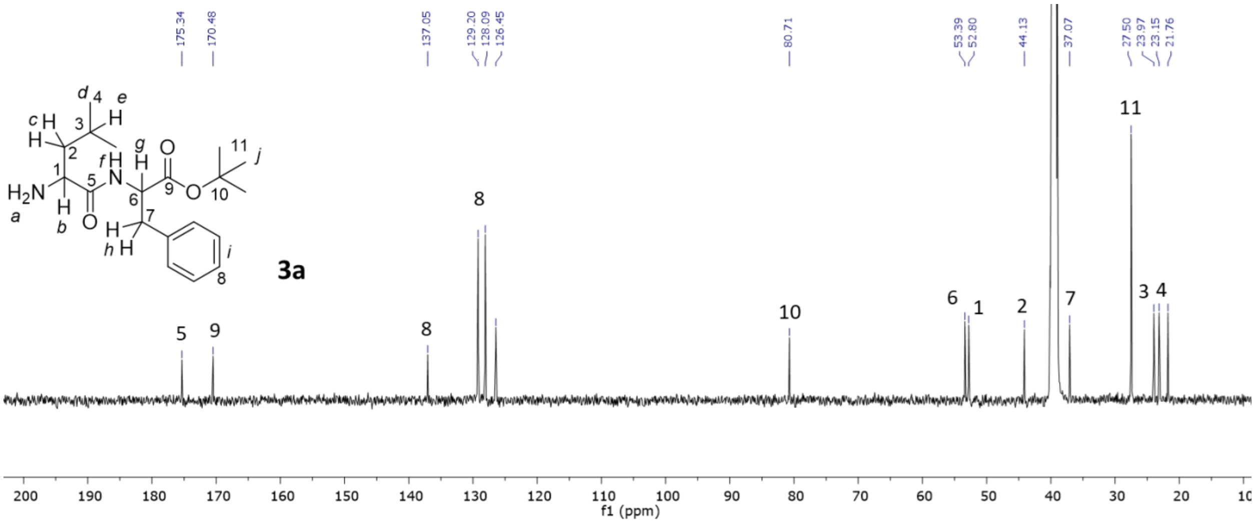

Figure S25. ^{13}C NMR (126 MHz, d_6-DMSO) spectrum of Leu-Phe-O*t*Bu **3a**.

2 Gelation and gel characterization

2.1 Gelation protocol

Gel systems **I**, **II** and **III**: The precursor gelator (**1**, **2**, and **3**, respectively) was suspended in *t*BuOAc solvent (0.05M) and sonicated until a fine suspension formed, followed by the addition of concentrated H_2SO_4 (1.0 equivalent). The mixture was gently swirled and left to rest at R.T. for at least 12 h. Gelation was verified by the vial inversion method.

Gel system **IV**: The precursor gelators **1** and **2** were suspended in *t*BuOAc (1:1 ratio, final concentration 0.05 M) and sonicated until a fine suspension formed, followed by the addition of concentrated H_2SO_4 (1.0 equivalent). The mixture was gently swirled and left to rest at R.T. for at least 12 h. Gelation was verified by the vial inversion method.

2.2 Gel characterization by NMR

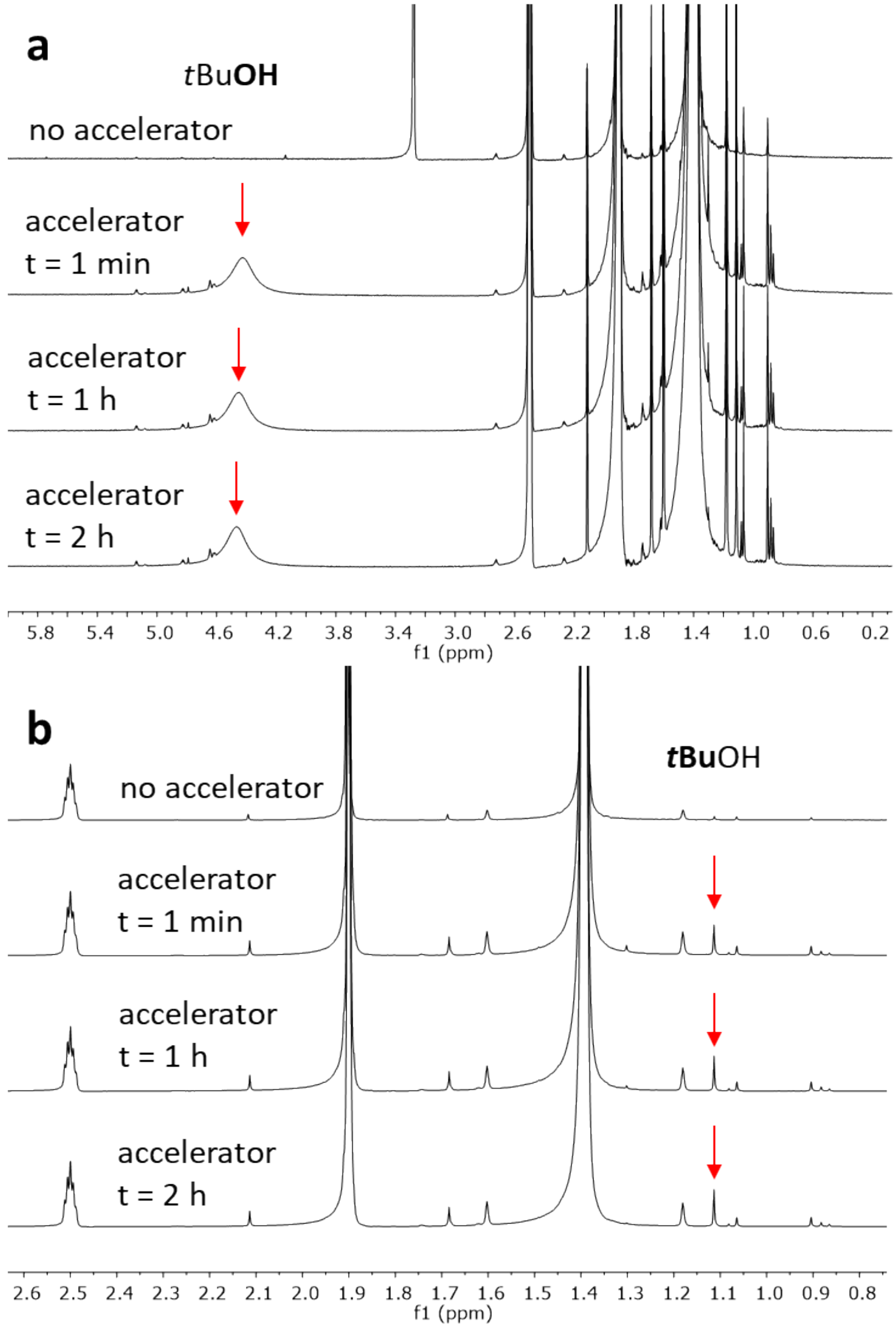

Figure S26. ^{1}H NMR (300 MHz, d_6-DMSO) spectrum of *t*BuOAc solvent in **a)** hydroxyl region and **b)** methyl region. Before addition of accelerator (top rows) and hourwise monitoring.

2.2.1 Gel system I: Precursor Boc-Phe-Phe-O*t*Bu 1

0.05 M, 1.0 eq H_2SO_4, day 1

^{1}H NMR (300 MHz, *d*$_6$-DMSO) δ 8.89 (d, *J* = 7.5 Hz, 1H), 8.83 (d, *J* = 7.8 Hz, 1H), 8.05 (d, *J* = 7.3 Hz, 6H), 7.37 – 7.21 (m, 20H), 4.58 – 4.46 (m, 1H), 4.04 (s, 2H), 3.13 (ddd, *J* = 13.8, 10.7, 5.4 Hz, 3H), 3.02 – 2.89 (m, 3H), 1.32 (s, 9H).

^{13}C NMR (75 MHz, *d*$_6$-DMSO) δ 172.7, 170.4, 168.6, 168.6, 137.6, 137.2, 135.1, 135.1, 130.1, 129.7, 129.6, 129.0, 128.8, 128.8, 127.7, 127.7, 127.2, 127.2, 81.7, 54.9, 54.3, 53.6, 37.5, 37.4, 37.2, 28.0.

HR-MS: *m/z* for $C_{22}H_{28}N_2O_3$ $[M + H]^+$ calculated 369.2100, found 369.2164. *m/z* for $C_{18}H_{20}N_2O_3$ $[M + H]^+$ calculated 313.1500, found 313.1536.

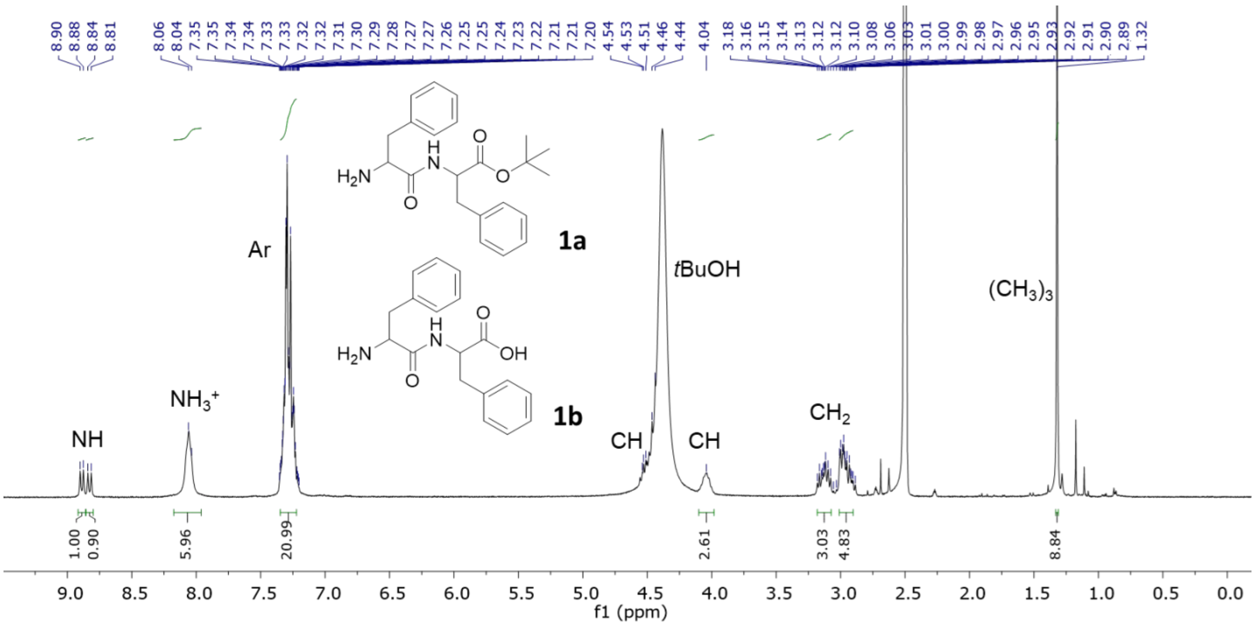

Figure S27. ^{1}H NMR (300 MHz, *d*$_6$-DMSO) spectrum of xerogel **I**, 0.05M, 1.0 equivalent of H_2SO_4, day 1.

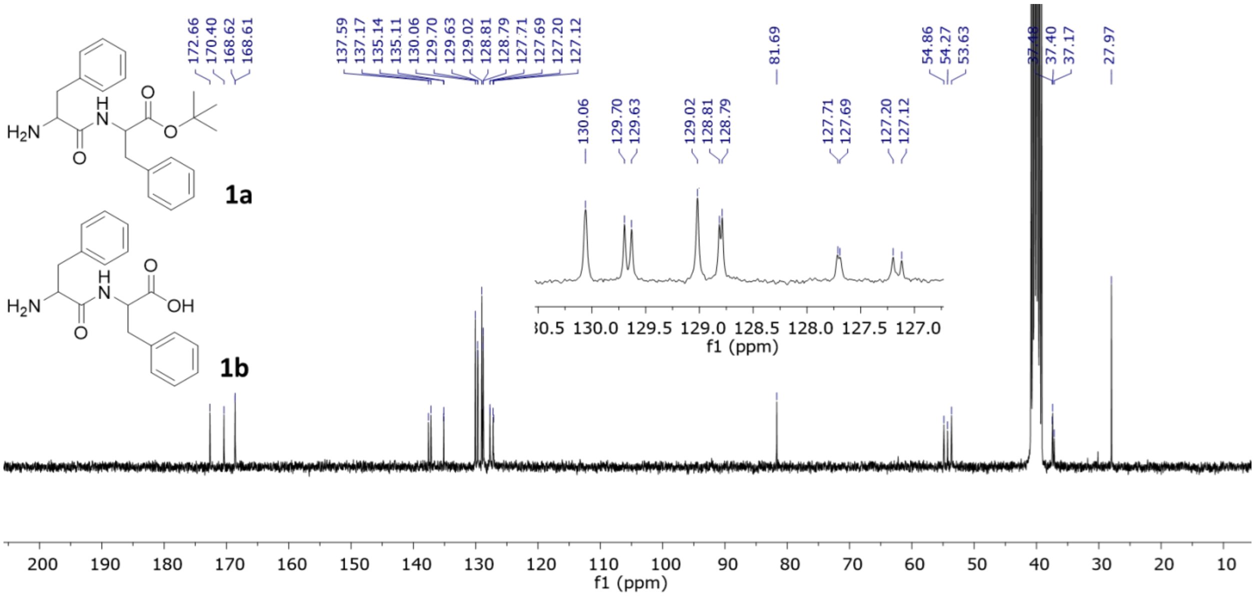

Figure S28. ^{13}C NMR (75 MHz, d_6-DMSO) spectrum of xerogel **I**, 0.05M, 1.0 equivalent of H_2SO_4, day 1.

0.05 M, 0.5 eq H_2SO_4, day 1

^{1}**H NMR** (300 MHz, d_6-DMSO) δ 8.89 (d, J = 7.5 Hz, 1H), 8.19 (d, J = 7.5 Hz, 0.6H), 8.07 (s, 3H), 7.36 – 7.17 (m, 18H), 6.82 (d, J = 8.6 Hz, 0.6H), 4.47 (t, J = 7.3 Hz, 1H), 4.19 (s, 1H), 4.05 (s, 1H), 3.02 – 2.87 (m, 6H), 2.75 – 2.63 (m, 1H), 1.32 (d, J = 2.1 Hz, 15H), 1.28 (s, 5H). Note: Presence of precursor marked by a star.

^{13}**C NMR** (126 MHz, d_6-DMSO) δ 169.9, 168.1, 136.7, 134.6, 129.6, 129.2, 128.5, 128.3, 127.2, 126.7, 81.2, 54.4, 53.1, 37.0, 36.9, 27.5.

HR-MS: *m/z* for $C_{22}H_{28}N_2O_3$ $[M + H]^+$ calculated 369.2100, found 369.2172. *m/z* for $C_{18}H_{20}N_2O_3$ $[M + H]^+$ calculated 313.1500, found 313.1564. *m/z* for $C_{27}H_{36}N_2O_5$ $[M + K]^+$ calculated 507.3500, found 507.2262.

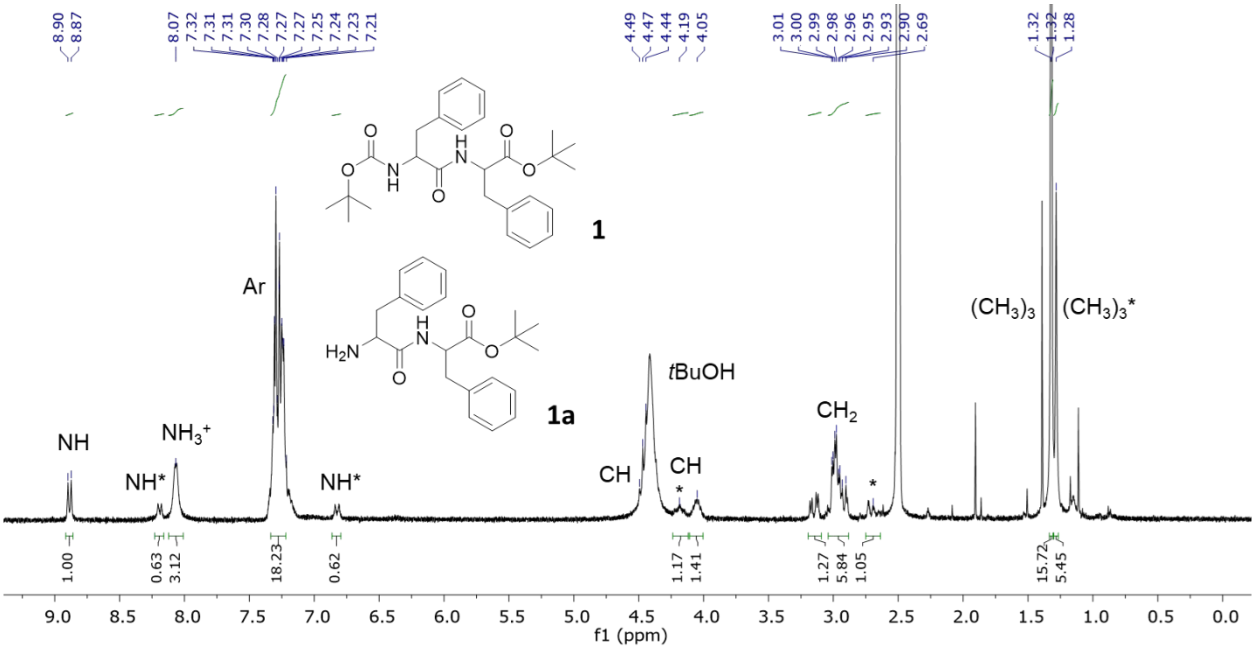

Figure S29. ^{1}H NMR (300 MHz, d_6-DMSO) spectrum of xerogel **I**, 0.05M, 0.5 equivalent of H_2SO_4, day 1.

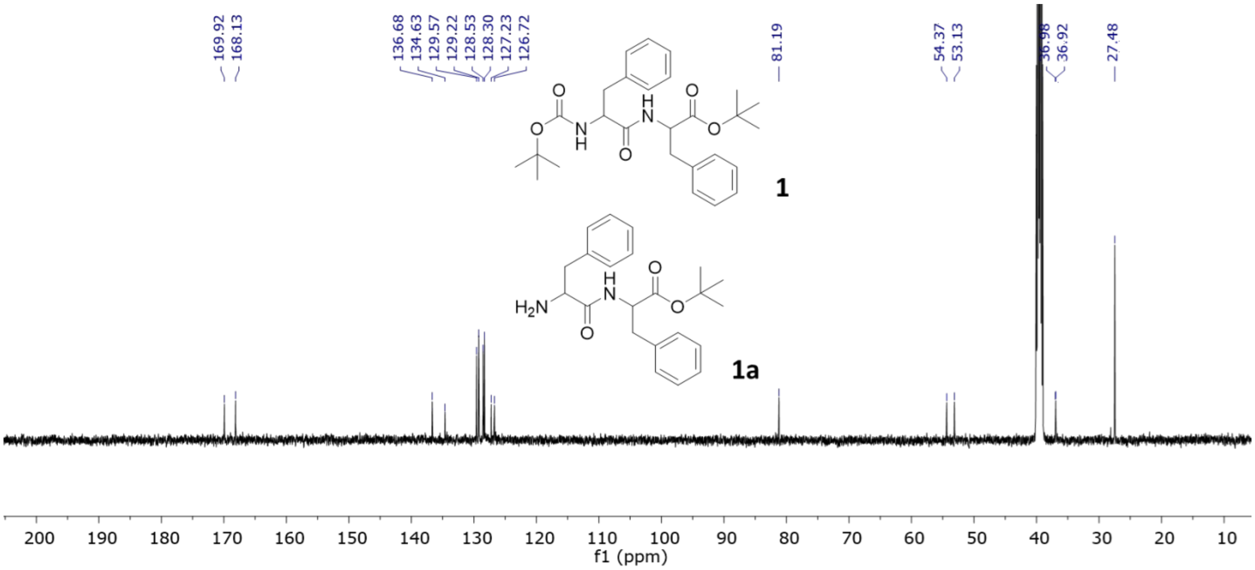

Figure S30. ^{13}C NMR (126 MHz, d_6-DMSO) spectrum of xerogel **I**, 0.05M, 0.5 equivalent of H_2SO_4, day 1.

2.2.2 Gel system II: Precursor Boc-Phe-Phe-Phe-O*t*Bu 2

0.05 M, 1.0 eq H_2SO_4, day 1

^{1}H NMR (500 MHz, d_6-DMSO) δ 8.73 (d, J = 8.3 Hz, 1H), 8.70 (d, J = 8.4 Hz, 0.2H), 8.61 (d, J = 7.5 Hz, 1H), 8.53 (d, J = 7.9 Hz, 0.2H), 8.33 (d, J = 7.7 Hz, 0.1H), 8.22 (d, J = 7.9 Hz, 0.1H), 7.98 (d, J = 5.5 Hz, 3.5H), 7.32 – 7.20 (m, 20H), 4.65 (tt, J = 9.7, 4.7 Hz, 1H), 4.50 (dt, J = 8.2, 3.9 Hz, 0.4H), 4.42 (q, J = 7.5 Hz, 1H), 4.35 (d, J = 7.4 Hz, 0.2H), 3.13 – 2.78 (m, 8H), 1.32 (s, 9H). Note: Presence of a rotamer (peaks at 8.33 ppm and 8.22 ppm), as described in section **2.1.2**.

^{13}C NMR (126 MHz, d_6-DMSO) δ 172.0, 170.6, 170.3, 167.9, 137.3, 137.0, 134.6, 129.6, 129.3, 129.2, 128.5, 128.2, 128.1, 127.1, 126.5, 126.4, 80.8, 66.9, 54.2, 53.8, 53.1, 37.8, 37.0, 36.9, 31.3, 27.8, 27.5, 21.1.

HR-MS: *m/z* for $C_{31}H_{37}N_3O_4$ $[M + H]^+$ calculated 516.2800, found 516.2865. *m/z* for $C_{27}H_{29}N_3O_4$ $[M + H]^+$ calculated 460.2200, found 460.2240.

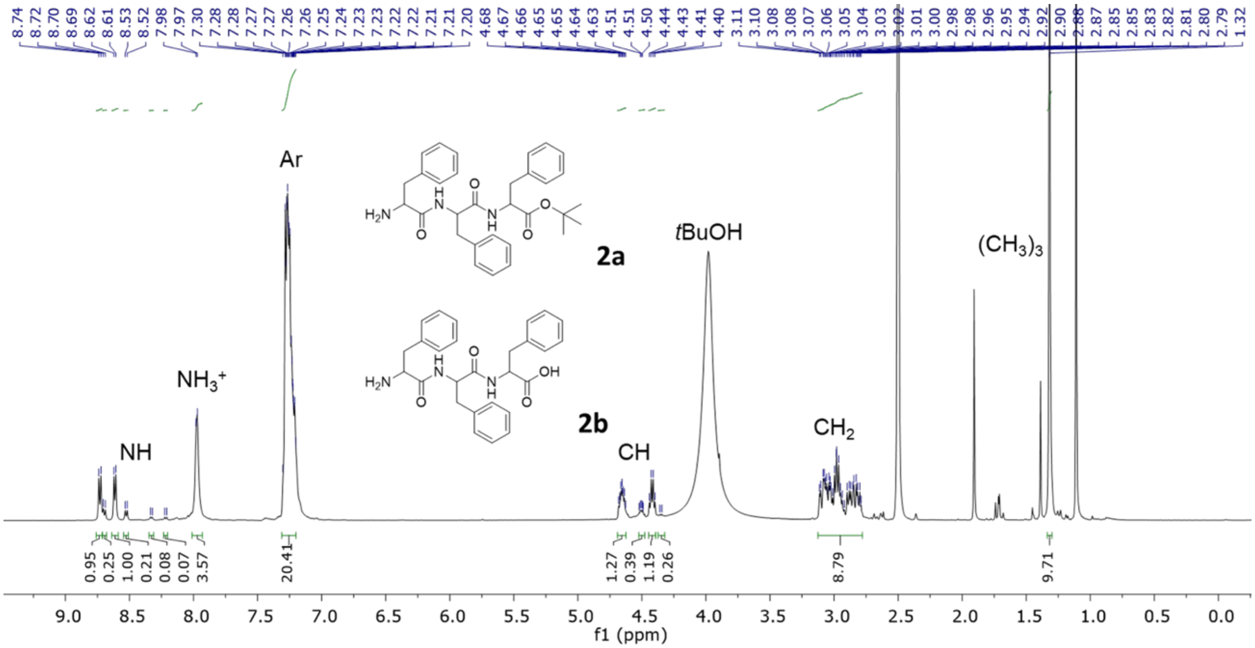

Figure S31. ^{1}H NMR (500 MHz, d_6-DMSO) spectrum of xerogel **II**, 0.05M, 1.0 equivalent of H_2SO_4, day 1. Presence of a rotamer.

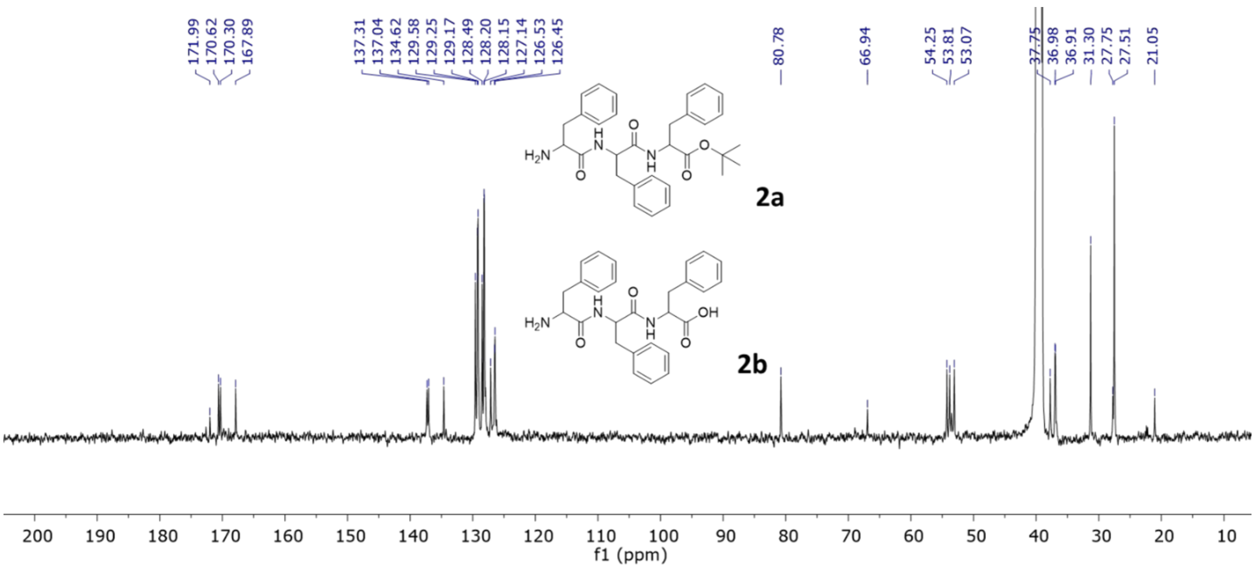

Figure S32. ^{1}H NMR (126 MHz, d_6-DMSO) spectrum of xerogel **II**, 0.05M, 1.0 equivalent of H_2SO_4, day 1.

0.05 M, 0.5 eq H_2SO_4, day 1

^{1}H NMR (500 MHz, *d*$_6$-DMSO) δ 8.7 (d, *J* = 8.3 Hz, 1H), 8.7 – 8.7 (m, 0.1H), 8.6 (d, *J* = 7.4 Hz, 1H), 8.6 – 8.5 (m, 0.1H), 8.5 (d, *J* = 7.4 Hz, 0.2H), 8.3 (d, *J* = 7.7 Hz, 0.1H), 8.1 – 8.0 (m, 0.2H), 8.0 (d, *J* = 5.3 Hz, 3H), 7.9 (d, *J* = 8.4 Hz, 0.3H), 7.3 – 7.2 (m, 21H), 6.8 (d, *J* = 8.8 Hz, 0.2H), 4.7 (td, *J* = 8.8, 4.4 Hz, 1H), 4.6 – 4.6 (m, 0.3H), 4.4 (q, *J* = 7.4 Hz, 1H), 4.4 (dd, *J* = 12.5, 5.1 Hz, 0.4H), 4.1 (d, *J* = 11.5 Hz, 0.6H), 4.0 (dt, *J* = 9.6, 4.7 Hz, 2H), 3.1 – 2.8 (m, 7H), 1.3 (s, 8H), 1.3 (s, 3.3H), 1.3 (s, 1.5H). Note: Presence of precursor marked by a star.

^{13}C NMR (126 MHz, *d*$_6$-DMSO) δ 170.6, 170.3, 167.9, 137.3, 137.0, 134.6, 129.6, 129.3, 129.2, 128.5, 128.2, 128.1, 127.9, 127.1, 126.5, 126.4, 80.8, 54.2, 53.8, 53.1, 37.7, 37.0, 36.9, 31.3, 28.1, 27.7, 27.5.

HR-MS: *m/z* for $C_{36}H_{45}N_3O_6$ $[M + H]^+$ calculated 616.3300, found 616.3286. *m/z* for $C_{31}H_{37}N_3O_4$ $[M + H]^+$ calculated 516.2800, found 516.2785. *m/z* for $C_{27}H_{29}N_3O_4$ $[M + H]^+$ calculated 460.2200, found 460.2153.

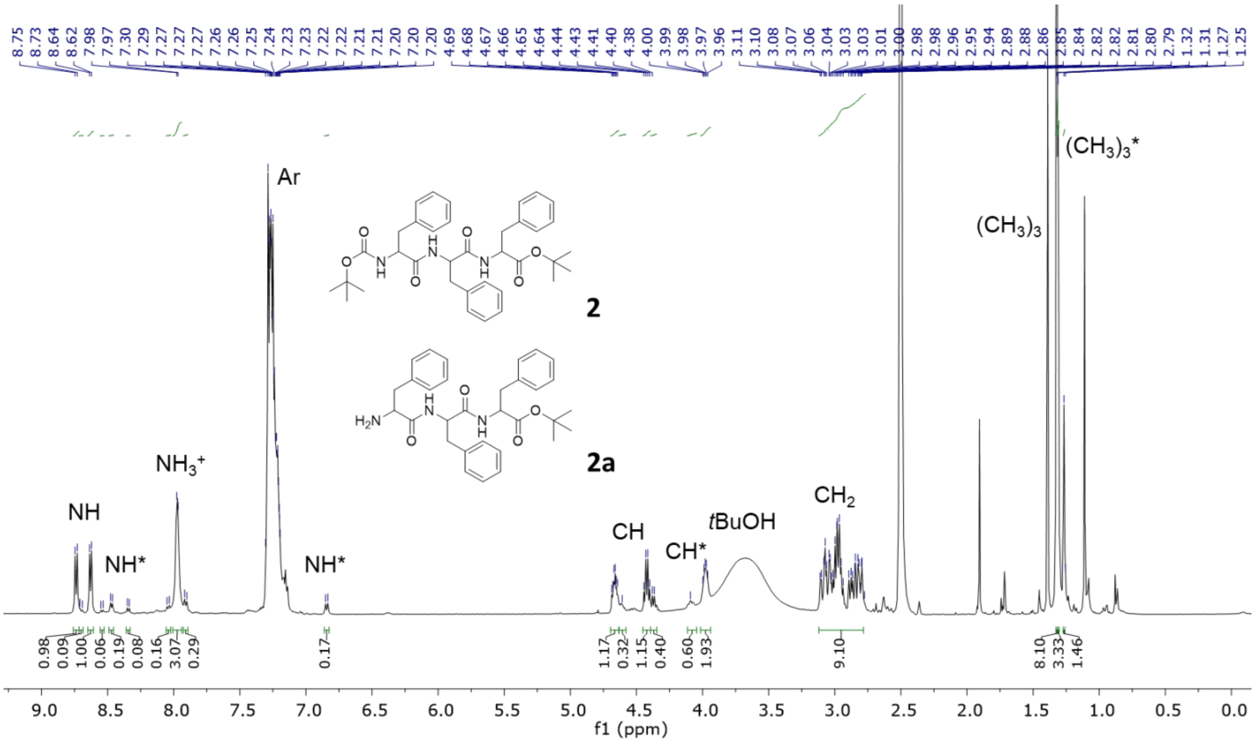

Figure S33. ^{1}H NMR (500 MHz, *d*$_6$-DMSO) spectrum of xerogel **II**, 0.05M, 0.5 equivalent of H_2SO_4, day 1.

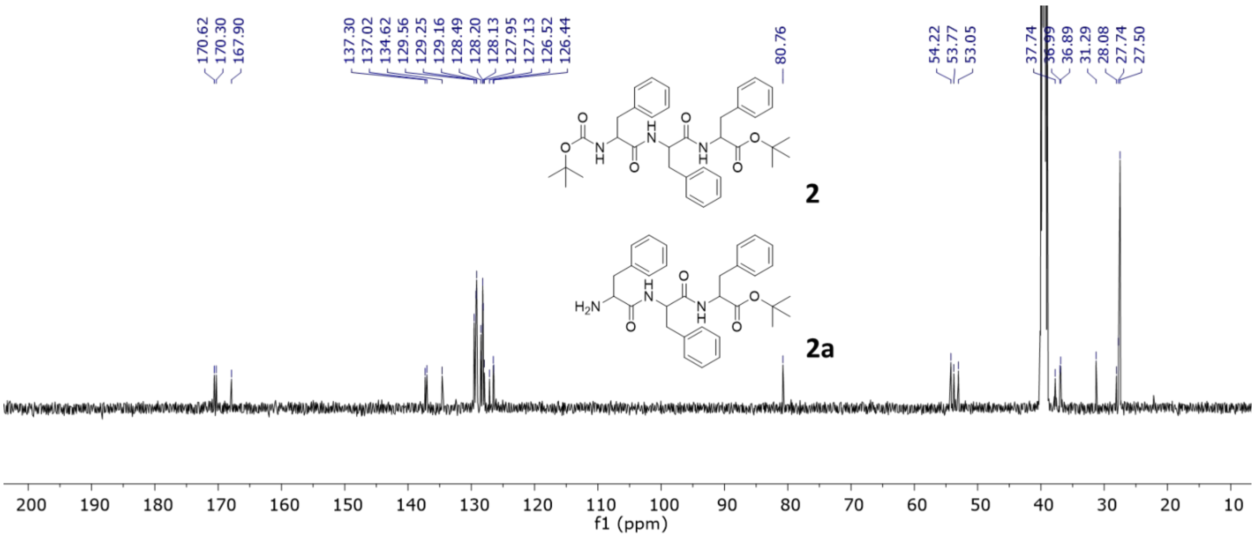

Figure S34. ^{1}H NMR (126 MHz, d_6-DMSO) spectrum of xerogel **II**, 0.05M, 0.5 equivalent of H_2SO_4, day 1.

0.05 M, 1.0 eq of H_2SO_4, day 5

^{1}H NMR (300 MHz, d_6-DMSO) δ 8.72 (d, J = 8.5 Hz, 1H), 8.67 (s, 0.2H), 8.60 (d, J = 7.5 Hz, 1H), 8.51 (d, J = 7.9 Hz, 0.2H), 8.31 (d, J = 7.4 Hz, 0.1H), 8.20 (d, J = 7.9 Hz, 0.1H), 8.02 – 7.93 (m, 4H), 7.25 (qd, J = 8.4, 7.0, 3.9 Hz, 22H), 4.66 (td, J = 8.7, 4.4 Hz, 1H), 3.98 (s, 1H), 3.15 – 2.76 (m, 5H), 1.32 (s, 9H).

^{13}C NMR (75 MHz, d_6-DMSO) δ 173.1, 171.9, 171.1, 170.8, 169.5, 168.4, 137.8, 137.5, 135.1, 130.1, 129.7, 129.6, 129.0, 128.7, 128.6, 128.4, 127.6, 127.0, 126.9, 81.3, 67.4, 54.7, 54.3, 53.6, 37.4, 31.8, 28.0.

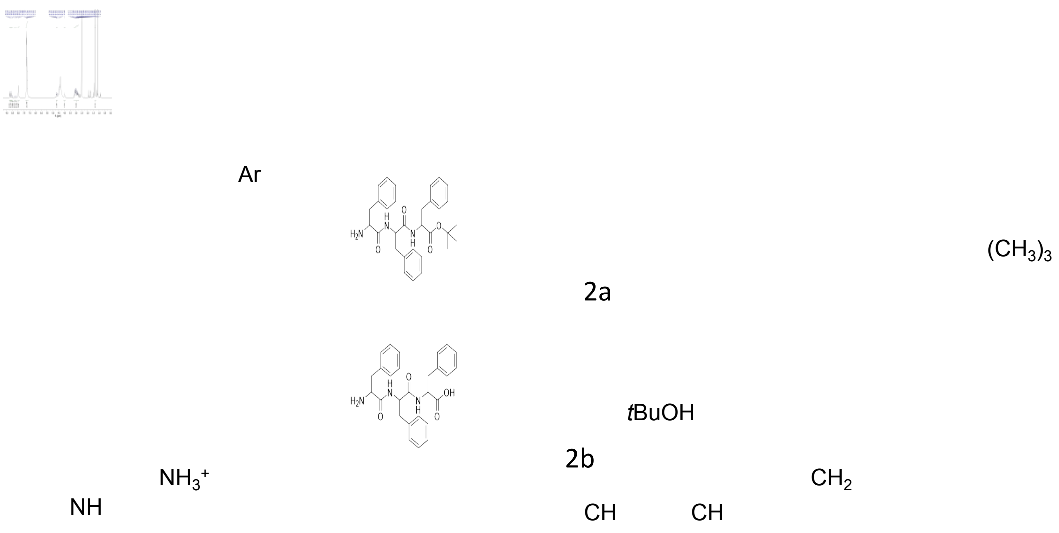

Figure S35. ^{1}H NMR (300 MHz, d_6-DMSO) spectrum of xerogel **II**, 0.05M, 1.0 equivalent of H_2SO_4, day 5.

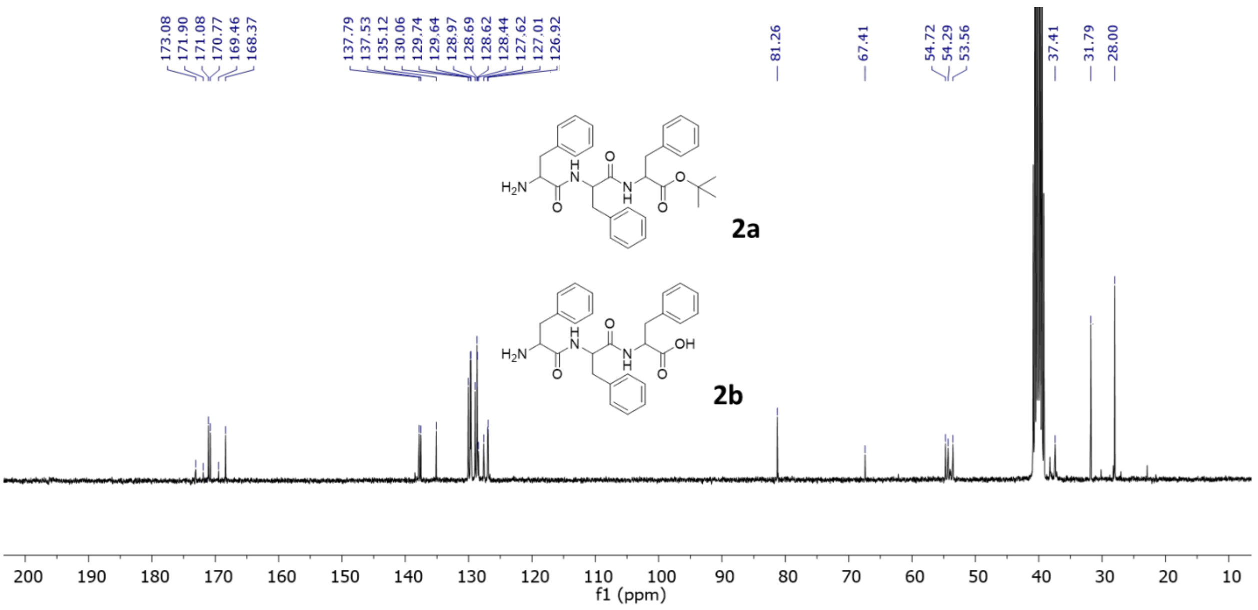

Figure S36. ^{1}H NMR (126 MHz, d_6-DMSO) spectrum of xerogel **II**, 0.05M, 1.0 equivalent of H_2SO_4, day 5.

2.2.3. Gel system III: Precursor Boc-Leu-Phe-O*t*Bu 3

0.05 M, 1.0 eq H_2SO_4, day 1

^{1}H NMR (300 MHz, d_6-DMSO) δ 8.83 (d, *J* = 7.4 Hz, 1H), 8.76 (d, *J* = 7.6 Hz, 0.7H), 8.06 (s, 5.2H), 7.35 – 7.21 (m, 8.8H), 4.54 – 4.39 (m, 1.9H), 3.76 (s, 1.7H), 3.14 – 2.92 (m, 3.8H), 1.67 (d, *J* = 6.9 Hz, 2.4H), 1.55 (d, *J* = 7.5 Hz, 3.8H), 1.31 (s, 9H), 0.95 – 0.86 (m, 10.9H).

^{13}C NMR (75 MHz, d_6-DMSO) δ 172.2, 169.2, 137.2, 129.1, 128.3, 126.6, 53.8, 50.7, 36.5, 27.5, 23.4, 22.9, 21.7.

HR-MS: *m/z* for $C_{19}H_{30}N_2O_3$ $[M + H]^+$ calculated 335.2300, found 335.2335. *m/z* for $C_{15}H_{22}N_2O_3$ $[M + H]^+$ calculated 279.1600, found 279.1716.

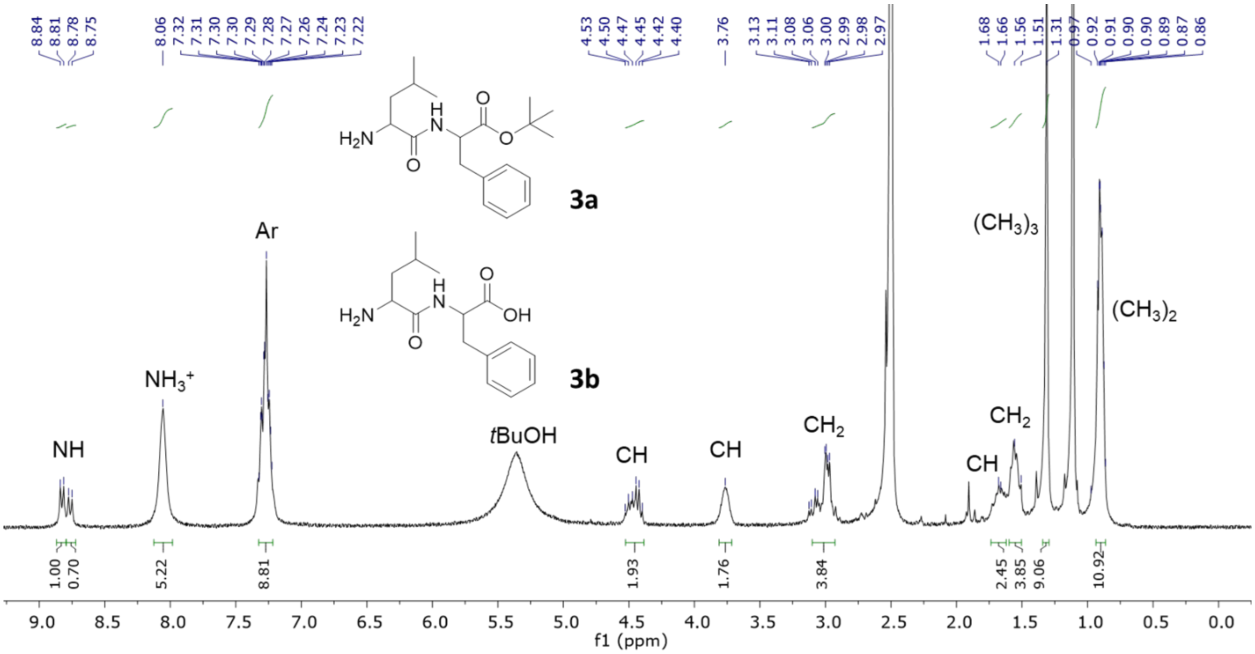

Figure S37. ^{1}H NMR (300 MHz, d_6-DMSO) spectrum of xerogel **III**, 0.05M, 1.0 equivalent of H_2SO_4, day 1.

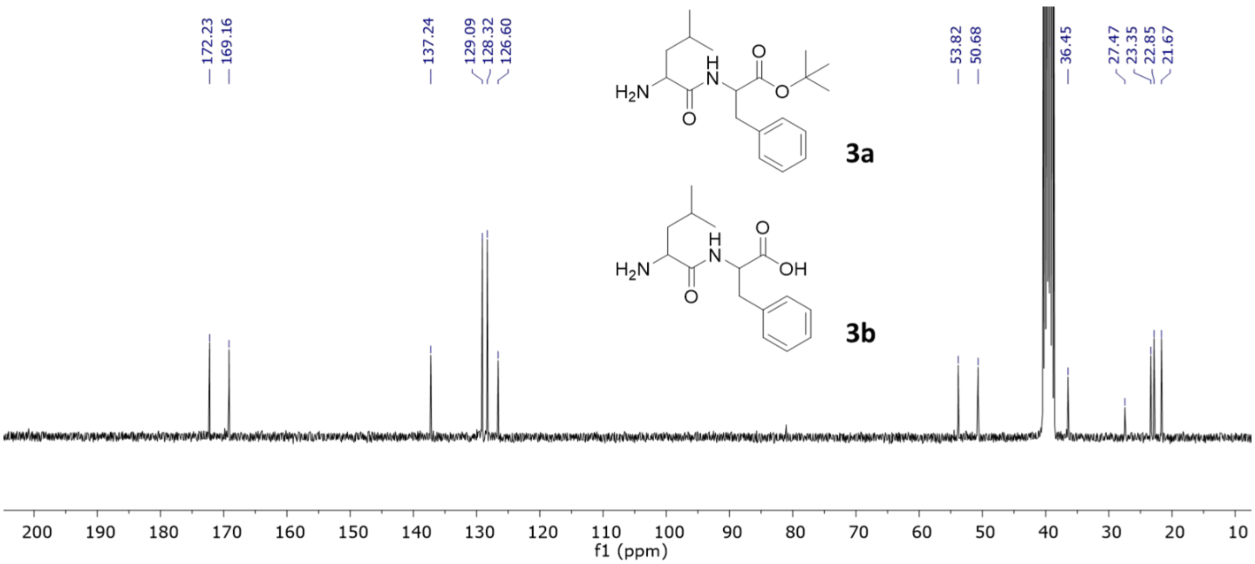

Figure S38. ^{13}C NMR (75 MHz, d_6-DMSO) spectrum of xerogel **III**, 0.05M, 1.0 equivalent of H_2SO_4, day 1.

0.05 M, 0.5 eq H_2SO_4, day 1

^{1}H NMR (300 MHz, d_6-DMSO) δ 8.83 (d, J = 7.4 Hz, 1H), 8.06 (s, 3H), 7.98 (d, J = 7.7 Hz, 1H), 7.35 – 7.18 (m, 10H), 6.79 (d, J = 8.6 Hz, 1H), 4.44 (q, J = 7.4 Hz, 1H), 4.35 (q, J = 7.4 Hz, 1H), 4.02 – 3.92 (m, 1H), 3.76 (d, J = 6.0 Hz, 1H), 3.05 – 2.89 (m, 4H), 1.67 (dq, J = 12.7, 6.5, 6.1 Hz, 1H), 1.61 – 1.52 (m, 2H), 1.37 (s, 8H), 1.31 (s, 18H), 0.91 (dd, J = 6.3, 3.7 Hz, 6H), 0.84 (dd, J = 8.4, 6.5 Hz, 6H). Note: Presence of precursor marked by a star. **^{13}C NMR** (126 MHz, d_6-DMSO) δ 172.3, 169.2, 137.3, 129.1, 128.3, 128.1, 126.6, 126.5, 81.1, 80.7, 78.0, 53.8, 50.7, 36.5, 28.2, 27.5, 23.4, 22.9, 21.7.

HR-MS: *m/z* for $C_{15}H_{22}N_2O_3$ $[M + H]^+$ calculated 279.1600, found 279.1629. *m/z* for $C_{19}H_{30}N_2O_3$ $[M + H]^+$ calculated 335.2300, found 335.2242. *m/z* for $C_{24}H_{38}N_2O_5$ $[M + H]^+$ calculated 435.2800, found 435.2864.

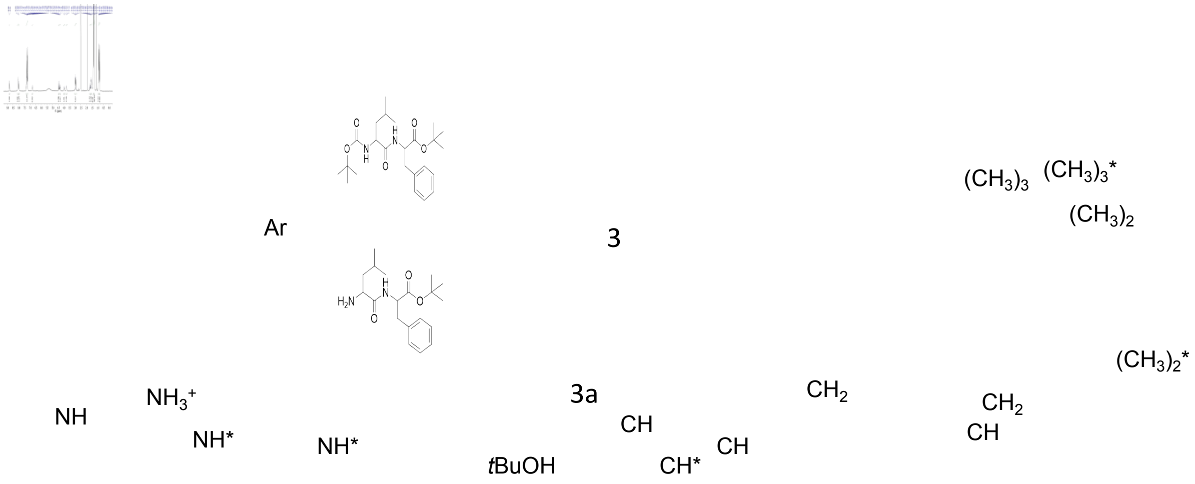

Figure S39. ^{1}H NMR (300 MHz, d_6-DMSO) spectrum of xerogel **III**, 0.05M, 0.5 equivalent of H_2SO_4, day 1.

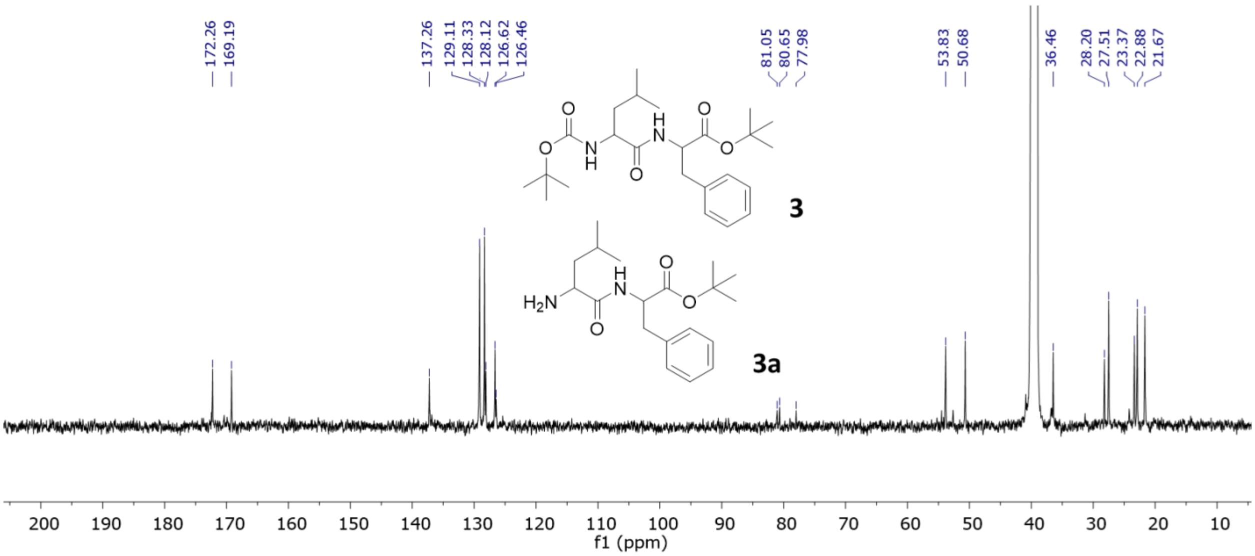

Figure S40. ^{13}C NMR (126 MHz, d_6-DMSO) spectrum of xerogel **III**, 0.05M, 0.5 equivalent of H_2SO_4, day 1.

0.05 M, 1.0 eq of H_2SO_4, day 10

^{1}H NMR (300 MHz, d_6-DMSO) δ 8.82 (d, J = 7.4 Hz, 1H), 8.76 (d, J = 7.8 Hz, 0.15H), 8.06 (s, 3.5H), 7.35 – 7.20 (m, 6H), 4.44 (q, J = 7.4 Hz, 1.3H), 3.76 (s, 1.3H), 3.07 – 2.93 (m, 2.3H), 1.66 (dt, J = 12.5, 6.3 Hz, 1.6H), 1.61 – 1.52 (m, 2.6H), 1.31 (s, 9H), 0.91 (dd, J = 6.3, 3.8 Hz, 7.7H).

^{13}C NMR (126 MHz, d_6-DMSO) δ 172.2, 169.8, 169.2, 169.1, 137.2, 136.8, 129.1, 129.1, 128.3, 128.3, 128.3, 126.7, 126.6, 81.0, 54.5, 53.8, 50.6, 50.6, 36.7, 36.4, 29.7, 27.5, 23.4, 23.3, 22.8, 22.8, 21.7, 21.6.

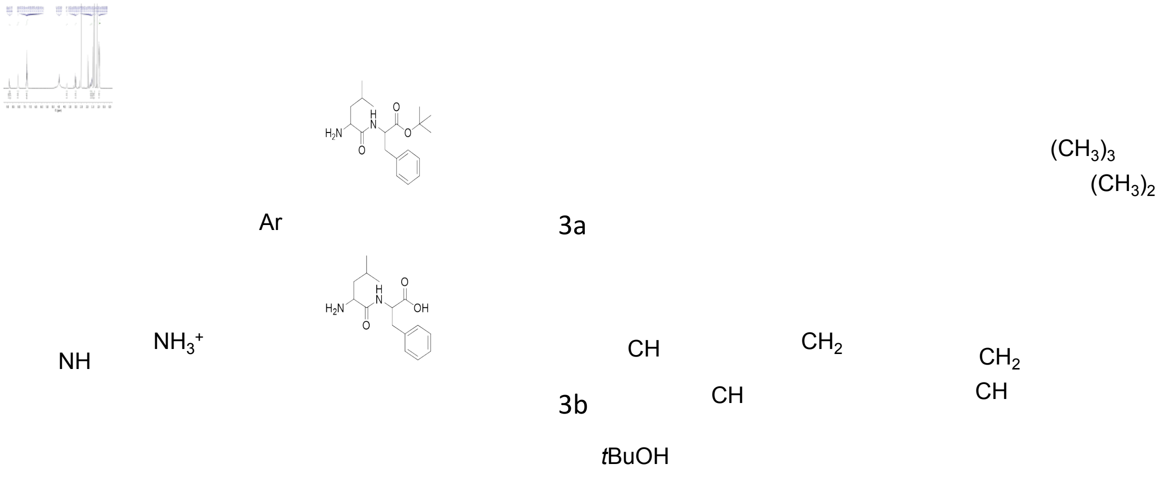

Figure S41. ^{1}H NMR (300 MHz, d_6-DMSO) spectrum of xerogel **III**, 0.05M, 1.0 equivalent of H_2SO_4, day 10.

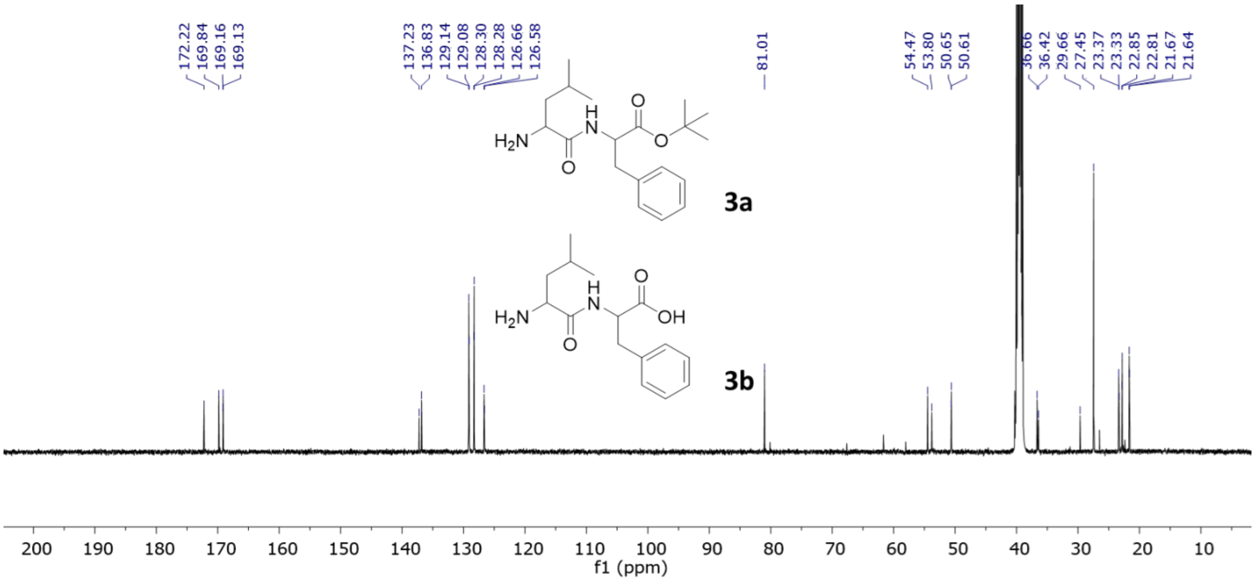

Figure S42. ^{13}C NMR (126 MHz, d_6-DMSO) spectrum of xerogel **III**, 0.05M, 1.0 equivalent of H_2SO_4, day 10.

2.2.4. Gel system IV: Precursor Boc-Phe-Phe-O*t*Bu 1 + Boc-Leu-Phe-O*t*Bu 3, 1:1 ratio

0.05 M, 1.0 eq H_2SO_4, day 1

^{1}H NMR (300 MHz, *d*$_6$-DMSO) δ 8.88 (d, *J* = 7.5 Hz, 0.6H), 8.82 (d, *J* = 7.5 Hz, 1H), 8.76 (d, *J* = 7.8 Hz, 0.3H), 8.06 (s, 5.6H), 7.36 – 7.21 (m, 15H), 4.48 (ddd, *J* = 19.1, 7.7, 5.3 Hz, 2.5H), 4.04 (s, 1H), 3.76 (s, 1H), 3.19 – 2.87 (m, 6H), 1.67 (q, *J* = 6.4 Hz, 1.2H), 1.60 – 1.49 (m, 2H), 1.32 (s, 4.6H), 1.32 (s, 5.3H), 0.96 – 0.86 (m, 7.2H). Note: this spectrum (system **IV**) is the superimpose of the individual gel spectra (systems **I** and **III**).

^{13}C NMR (75 MHz, *d*$_6$-DMSO) δ 172.7, 172.7, 169.6, 168.6, 137.7, 137.6, 135.1, 130.1, 129.7, 129.6, 129.6, 129.0, 128.8, 128.8, 127.7, 127.1, 127.1, 54.3, 53.6, 51.2, 37.4, 37.2, 36.9, 28.0, 23.8, 23.3, 22.2.

HR-MS: *m/z* for $C_{15}H_{22}N_2O_3$ $[M + H]^+$ calculated 279.1600, found 279.1703. *m/z* for $C_{18}H_{20}N_2O_3$ $[M + H]^+$ calculated 313.1500, found 313.1552. *m/z* for $C_{19}H_{30}N_2O_3$ $[M + H]^+$ calculated 335.2300, found 335.2249. *m/z* for $C_{22}H_{28}N_2O_3$ $[M + H]^+$ calculated 369.2100, found 369.2095.

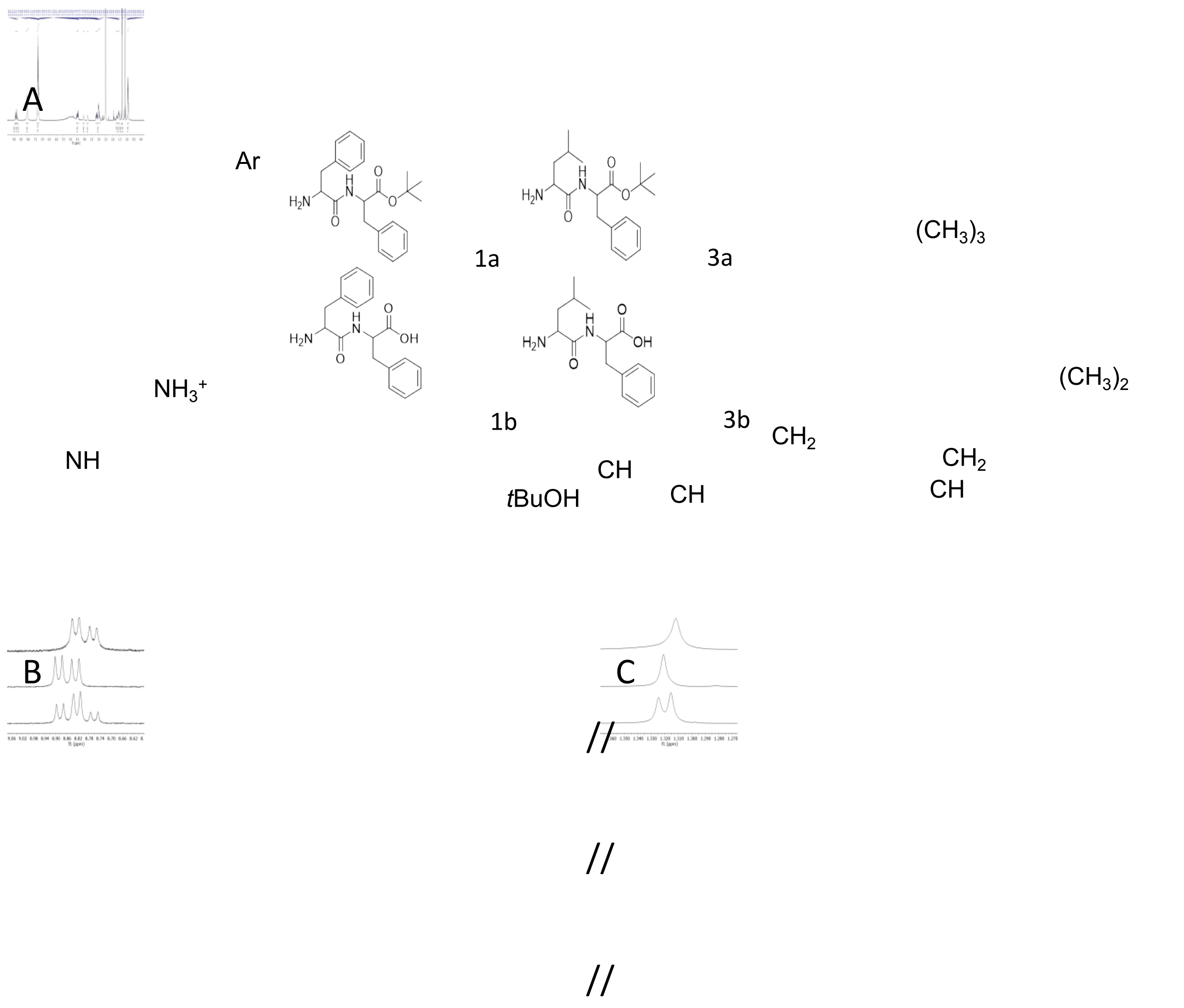

Figure S43. ^{1}H NMR (300 MHz, d_6-DMSO) spectrum of **(A)** xerogel **IV**, 0.05M, 1.0 equivalent of H_2SO_4, day 1, **(B)** N-H region expansion showing system **III** (top), system **I** (middle) and system **IV** (bottom) and **(C)** *t*Bu protective group region expansion showing system **III** (top), system **I** (middle) and system **IV** (bottom).

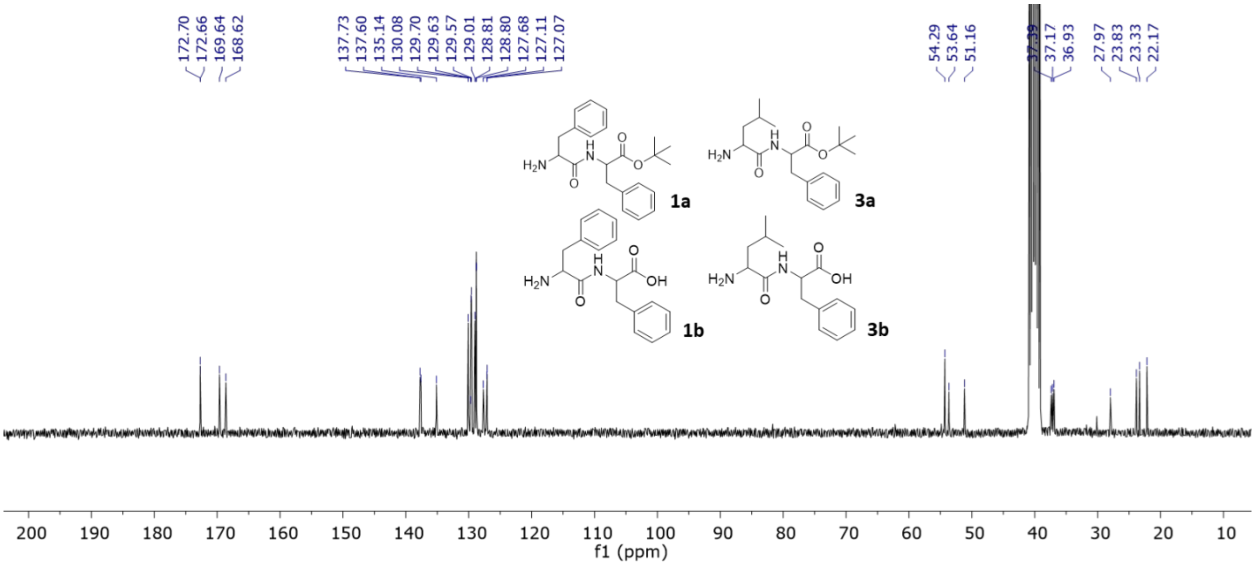

Figure S44. ^{13}C NMR (75 MHz, d_6-DMSO) spectrum of xerogel **IV**, 0.05M, 1.0 equivalent of H_2SO_4, day 1.

0.05 M, 0.5 eq H_2SO_4, day 1

^{1}H NMR (300 MHz, d_6-DMSO) δ 8.89 (d, J = 7.5 Hz, 1H), 8.83 (d, J = 7.4 Hz, 0.9H), 8.19 (d, J = 7.4 Hz, 1H), 8.07 (s, 6H), 7.98 (d, J = 7.6 Hz, 1H), 7.27 (ddt, J = 16.0, 13.3, 5.4 Hz, 30H), 6.81 (t, J = 9.3 Hz, 2H), 4.41 (ddd, J = 25.5, 11.2, 6.3 Hz, 3H), 4.19 (s, 1H), 4.06 (s, 1H), 4.01 – 3.92 (m, 1H), 3.77 (s, 1H), 3.21 – 2.87 (m, 10H), 1.69 (td, J = 13.3, 12.4, 5.5 Hz, 1H), 1.61 – 1.50 (m, 3H), 1.37 (s, 6H), 1.32 (s, 10H), 1.31 (s, 18H), 1.28 (s,59H), 0.91 (dd, J = 6.3, 3.7 Hz, 6H), 0.84 (dd, J = 8.4, 6.5 Hz, 7H). Note: Presence of precursor marked by a star.

^{13}C NMR (75 MHz, d_6-DMSO) δ 172.7, 172.7, 170.4, 170.3, 169.7, 168.6, 137.7, 137.6, 137.3, 137.2, 135.2, 135.1, 130.1, 129.7, 129.7, 129.6, 129.0, 128.8, 127.7, 127.2, 127.1, 127.07, 81.7, 81.5, 55.0, 54.9, 54.3, 53.7, 51.2, 37.5, 37.4, 37.2, 37.0, 27.98, 27.95, 23.9, 23.8, 23.3, 22.2.

HR-MS: *m/z* for $C_{15}H_{22}N_2O_3$ calculated 279.1600, found 279.1621. *m/z* for $C_{18}H_{20}N_2O_3$ [M + H]$^+$ calculated 313.1500, found 313.1472. *m/z* for $C_{19}H_{30}N_2O_3$ [M + H]$^+$ calculated 335.2300, found 335.2245. *m/z* for $C_{22}H_{28}N_2O_3$ [M + H]$^+$ calculated 369.2100, found 369.2090. *m/z* for

$C_{24}H_{38}N_2O_5$ $[M + H]^+$ calculated 435.2800, found 435.2768. *m/z* for $C_{27}H_{36}N_2O_5$ $[M + H]^+$ calculated 469.2600, found 469.2603.

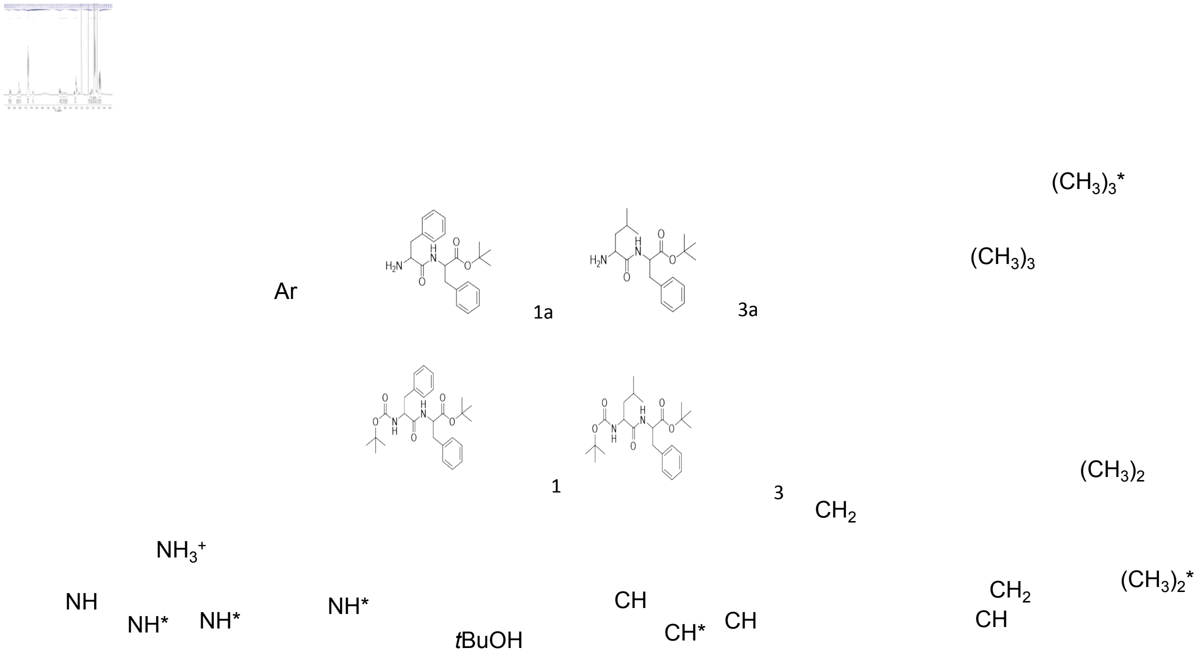

Figure S45. ^{1}H NMR (300 MHz, d_6-DMSO) spectrum of xerogel **IV**, 0.05M, 0.5 equivalent of H_2SO_4, day 1.

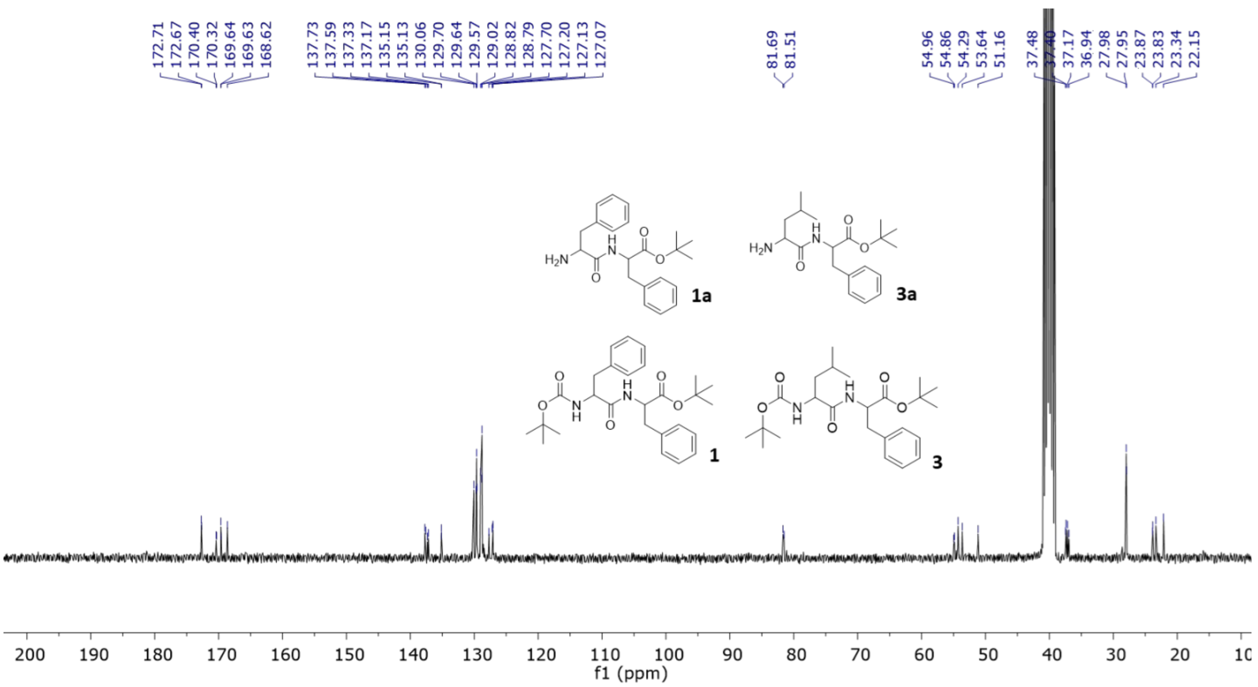

Figure S46. ^{13}C NMR (75 MHz, d_6-DMSO) spectrum of xerogel **IV**, 0.05M, 0.5 equivalent of H_2SO_4, day 1.

0.05 M, 1.0 eq H_2SO_4, day 5.

^{1}H NMR (300 MHz, d_6-DMSO) δ 8.90 (d, J = 7.5 Hz, 0.8H), 8.83 (d, J = 7.4 Hz, 1H), 8.77 (d, J = 7.8 Hz, 0.2H), 8.07 (d, J = 5.5 Hz, 6H), 7.36 – 7.20 (m, 16H), 4.53 – 4.38 (m, 2H), 4.06 (s, 1H), 3.77 (d, J = 7.7 Hz, 1H), 3.16 – 2.89 (m, 6H), 1.75 – 1.61 (m, 1H), 1.56 (td, J = 6.8, 2.8 Hz, 2H), 1.32 (s, 5H), 1.31 (s, 6H), 0.89 (dq, J = 8.4, 5.1, 4.5 Hz, 7H).

^{13}C NMR (126 MHz, d_6-DMSO) δ 172.3, 172.2, 169.2, 168.2, 137.3, 137.1, 134.7, 129.6, 129.2, 129.1, 128.6, 128.4, 128.3, 127.2, 126.7, 126.6, 61.7, 53.8, 53.8, 53.2, 50.7, 36.9, 36.7, 36.5, 29.7, 23.4, 22.9, 21.7.

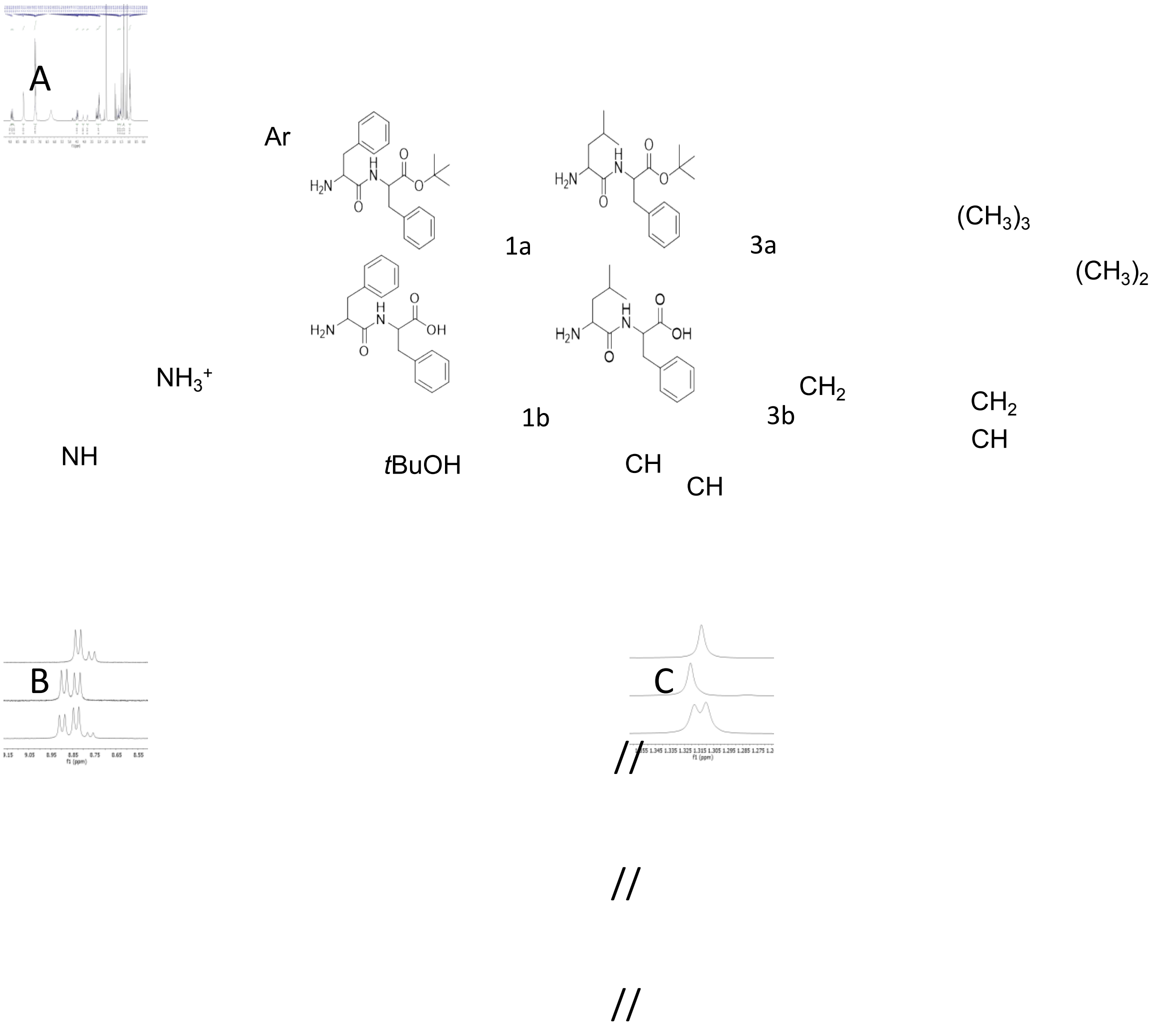

Figure S47. ^{1}H NMR (300 MHz, d_6-DMSO) spectrum of **(A)** xerogel **IV**, 0.05M, 1.0 equivalent of H_2SO_4, day 5, **(B)** N-H region expansion showing system **III** (top), system **I** (middle) and system **IV** (bottom) and **(C)** *t*Bu protective group region expansion showing system **III** (top), system **I** (middle) and system **IV** (bottom).

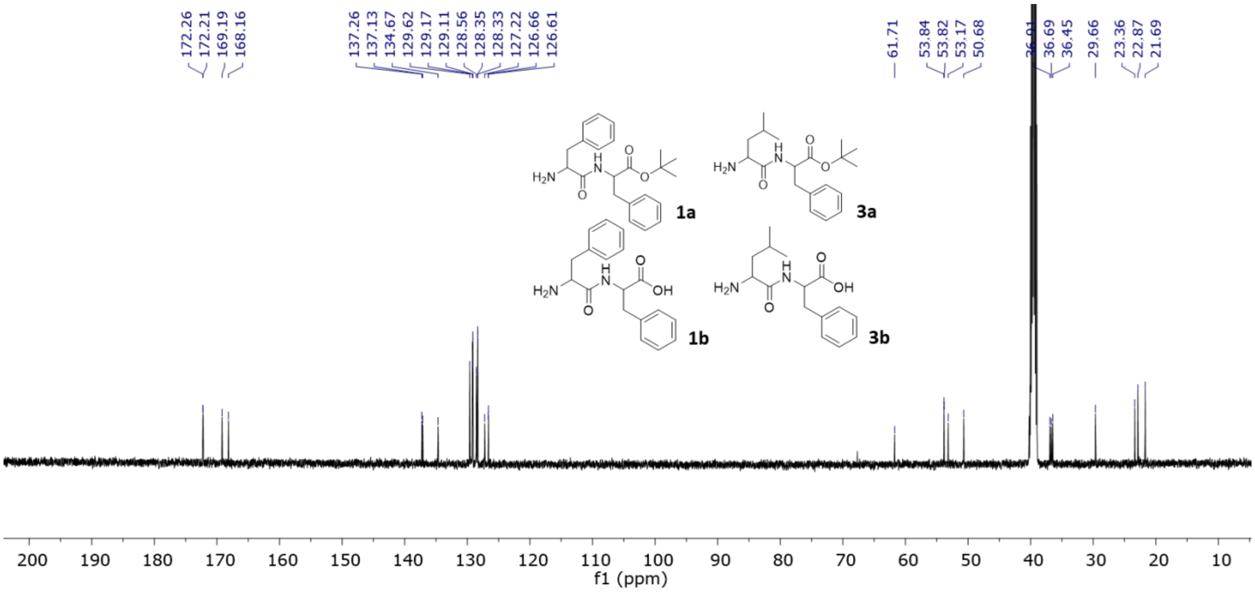

Figure S48. ^{13}C NMR (126 MHz, d_6-DMSO) spectrum of xerogel **IV**, 0.05M, 1.0 equivalent of H_2SO_4, day 5.

2.2.5. Gelation attempt in DCM

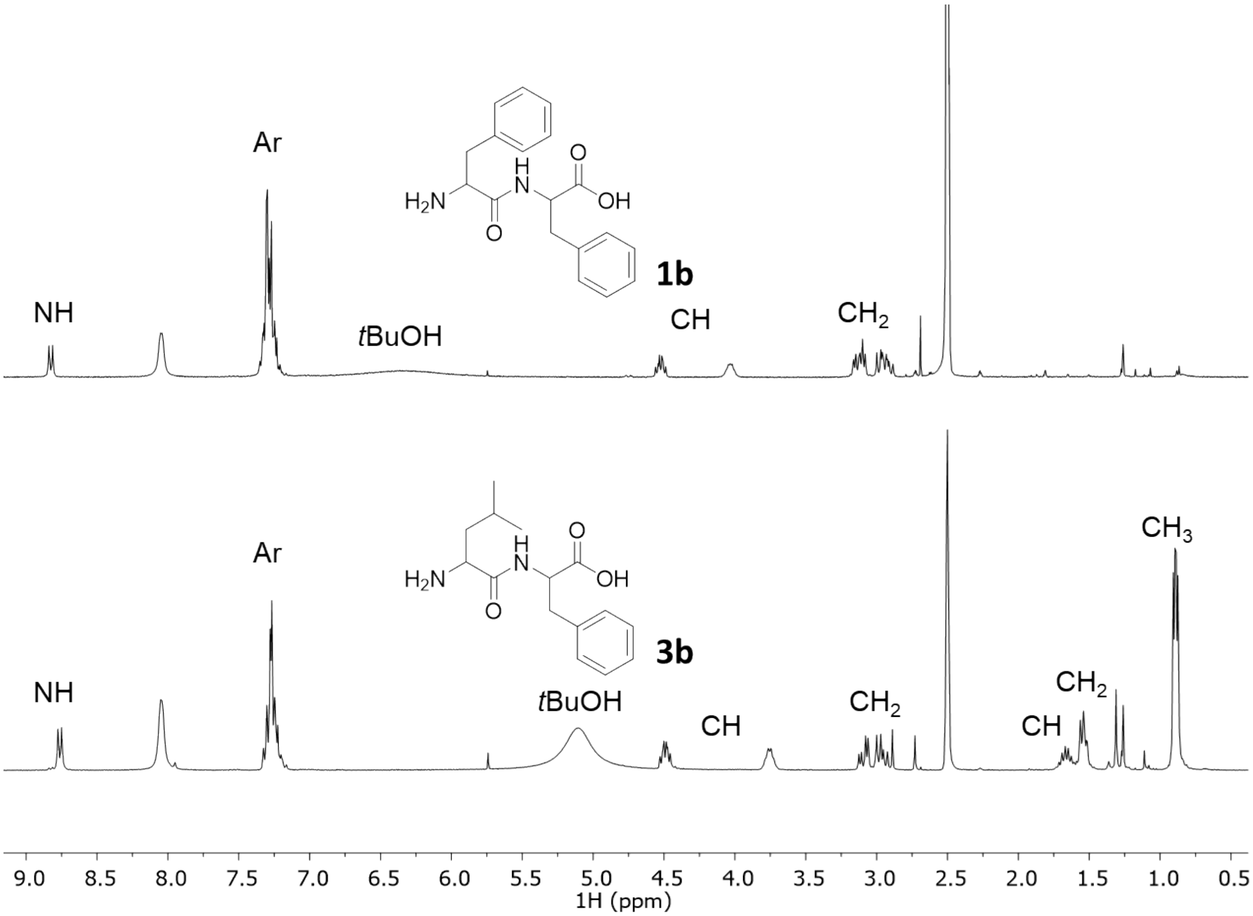

Figure S49. ^{1}H NMR (300 MHz, d_6-DMSO) spectrum of precipitates in DCM.

2.3. Solvatochromism and concentration study by UV-vis

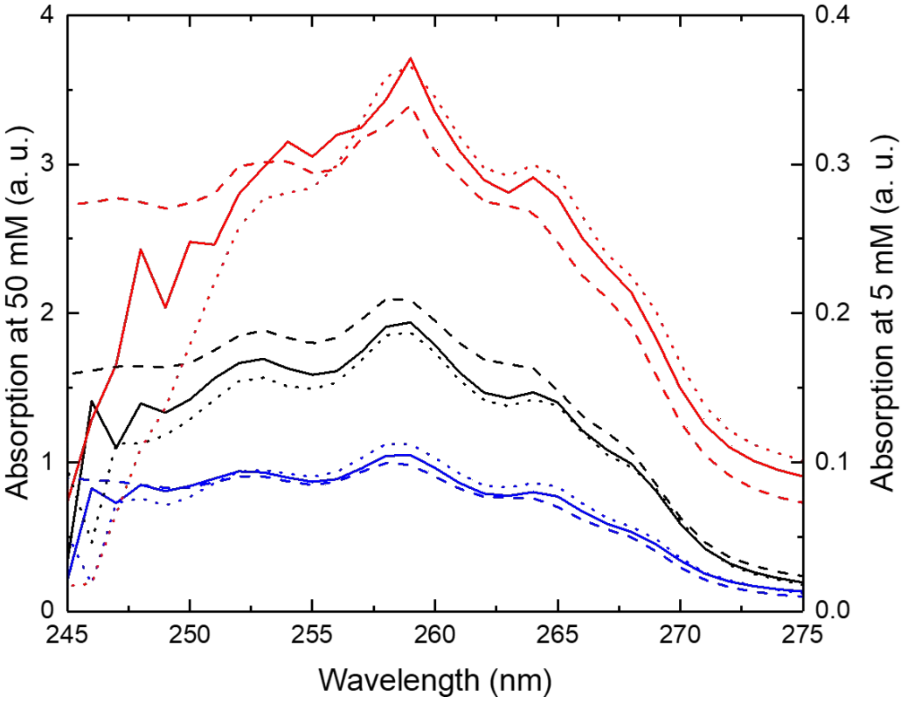

Figure S50. UV spectra of precursor gelators **1** (black), **2** (red) and **3** (blue) at 5 mM (plane) and 50 mM (dash) in *t*BuOAc and 50 mM in ACN (dots).

2.4. Phase-transition temperature

Phase-transition temperatures were measured by gradually heating the gel specimens starting from 25 ˚C, by 5 ˚C increments at 10 min intervals. The self-supporting character of the gels was assessed by the vial inversion method.

Table S1*. Phase transition temperature (˚C) of gel systems* ***I, II, III*** *and* ***IV*** *depending on the gel concentration.*

$T_{gel-sol}$ (˚C)	Gel concentration

	0.025 M	0.05 M	0.1 M
Gel system I	45	50	55
Gel system II	55	65	65
Gel system III	40	45	50
Gel system IV	35	40	45

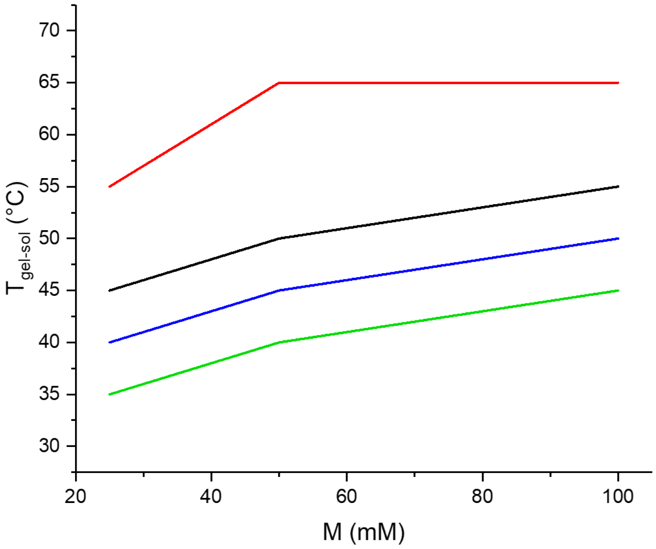

Figure S51. Phase transition temperature in function of gel concentration for gel system **I** (black), **II** (red), **III** (blue) and **IV** (green).

2.5. Gelation concentration screening

Table S2*. Concentration screening trials of gel system* ***I*** *in tBuOAc solvent (1.0 mL) at R.T.*

M	Boc-Phe-Phe-O*t*Bu **1** (mg)	H_2SO_4	H_2SO_4	Gelation outcome	Lifespan

(mol/L)		(eq)	(μL)		(day)
0.1	47	1.5	7.8	SSG[a], opaque	3
0.1	47	1.0	5.3	SSG, opaque	15-17
0.1	47	0.5	2.7	SSG, opaque	/[b]
0.05	23.5	1.5	4	SSG, opaque	3-4
0.05	23.5	1.0	2.7	SSG, opaque	4-6
0.05	23.5	0.5	1.3	SSG, opaque	/
0.025	11.8	1.5	2	SSG, opaque	n.m.[c]
0.025	11.8	1.0	1.3	SSG, opaque	n.m.
0.025	11.8	0.5	0.6	SSG, opaque	n.m.

[a] self-supporting gel [b] no transitivity [c] not measured

***Table S3**. Concentration screening trials of gel system **II** in tBuOAc solvent (1.0 mL) at R.T.*

M (mol/L)	Boc-Phe-Phe-Phe-O*t*Bu **2** (mg)	H_2SO_4 (eq)	H_2SO_4 (μL)	Gelation outcome	Lifespan (day)
0.1	61.5	1.0	5.3	SSG[a], opaque	15
0.1	61.5	0.5	2.7	SSG, opaque	/[b]
0.05	30.8	1.5	4.0	SSG, opaque	5-7
0.05	30.8	1.0	2.7	SSG, opaque	7-11
0.05	30.8	0.5	1.3	SSG, opaque	/
0.025	15.4	1.0	1.3	SSG, opaque	11

0.025	15.4	0.5	0.6	SSG, opaque	/

[a] self-supporting gel [b] no transitivity

***Table S4**. Concentration screening trials of gel system **III** in tBuOAc solvent (1.0 mL) at R.T.*

M (mol/L)	Boc-Leu-Phe-O*t*Bu **3** (mg)	H_2SO_4 (eq)	H_2SO_4 (μL)	Gelation outcome	Lifespan (day)
0.1	43.4	1.5	7.8	SSG[a], transparent	4-5
0.1	43.4	1.0	5.3	SSG, transparent	25
0.1	43.4	0.5	2.7	SSG, transparent	/[b]
0.05	21.7	1.5	4.0	SSG, transparent	1-2
0.05	21.7	1.0	2.7	SSG, transparent	22
0.05	21.7	0.5	1.3	SSG, transparent	/
0.025	10.8	1.5	2.0	SSG, transparent	18-20
0.025	10.8	1.0	1.3	SSG, transparent	28-34
0.025	10.8	0.5	0.6	SSG, transparent	/

[a] self-supporting gel [b] no transitivity

***Table S5**. Concentration screening trials of gel system **IV** in tBuOAc solvent (1.0 mL) at R.T.*

M (mol/L)	Boc-Phe-Phe-O*t*Bu **1** (mg)	Boc-Leu-Phe-O*t*Bu **3** (mg)	H_2SO_4 (eq)	H_2SO_4 (μL)	Gelation outcome	Lifespan (day)
0.1	23.5	21.7	1.5	7.8	SSG[a], transparent	1-2

0.1	23.5	21.7	1.0	5.3	SSG, transparent	10
0.1	23.5	21.7	0.5	2.7	SSG, transparent	/[b]
0.05	11.8	10.8	1.5	4.0	SSG, transparent	1-2
0.05	11.8	10.8	1.0	2.7	SSG, transparent	7-9
0.05	11.8	10.8	0.5	1.3	SSG, transparent	/
0.025	5.8	5.4	1.5	2.0	solution	n.m.[c]
0.025	5.8	5.4	1.0	1.3	SSG, transparent	5
0.025	5.8	5.4	0.5	0.6	SSG, transparent	/

[a] self-supporting gel [b] no transitivity [c] not measured

3. FTIR spectra of compounds 1-8, 1a-3a and xerogels I-IV

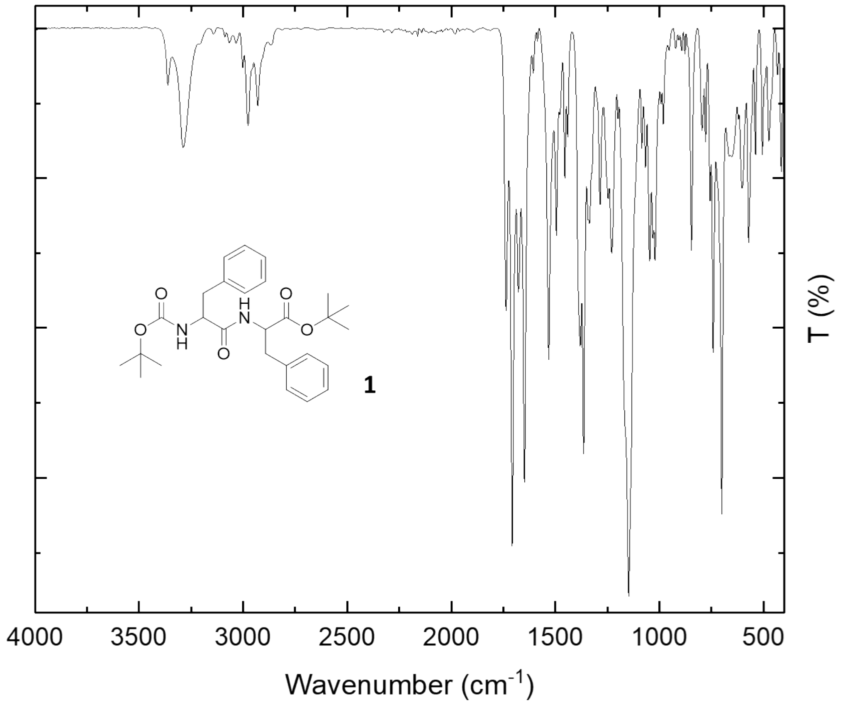

Figure S52. ATR-FTIR spectrum of Boc-Phe-Phe-O*t*Bu 1.

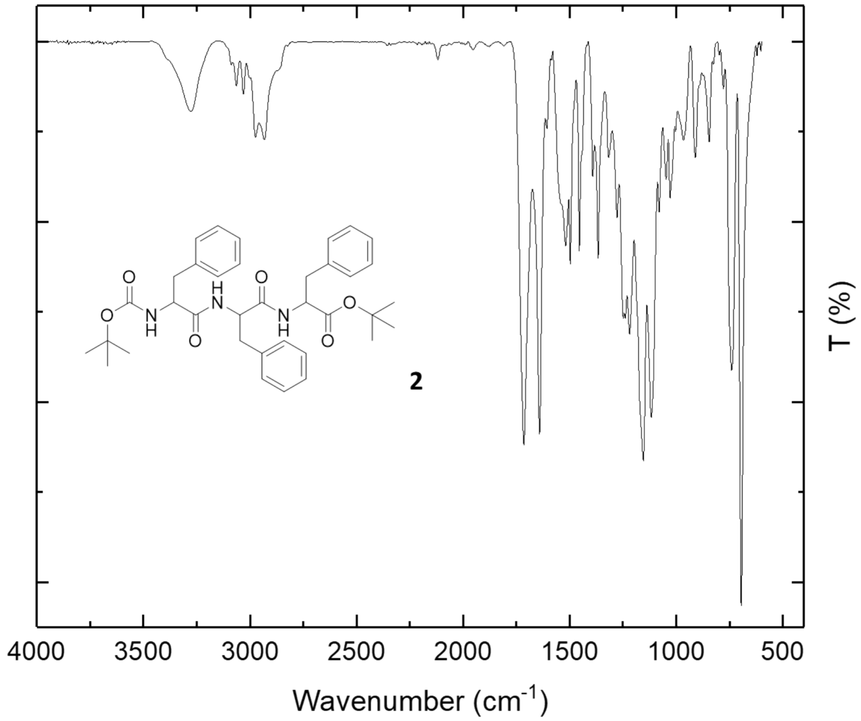

Figure S53. ATR-FTIR spectrum of Boc-Phe-Phe-Phe-O*t*Bu **2**.

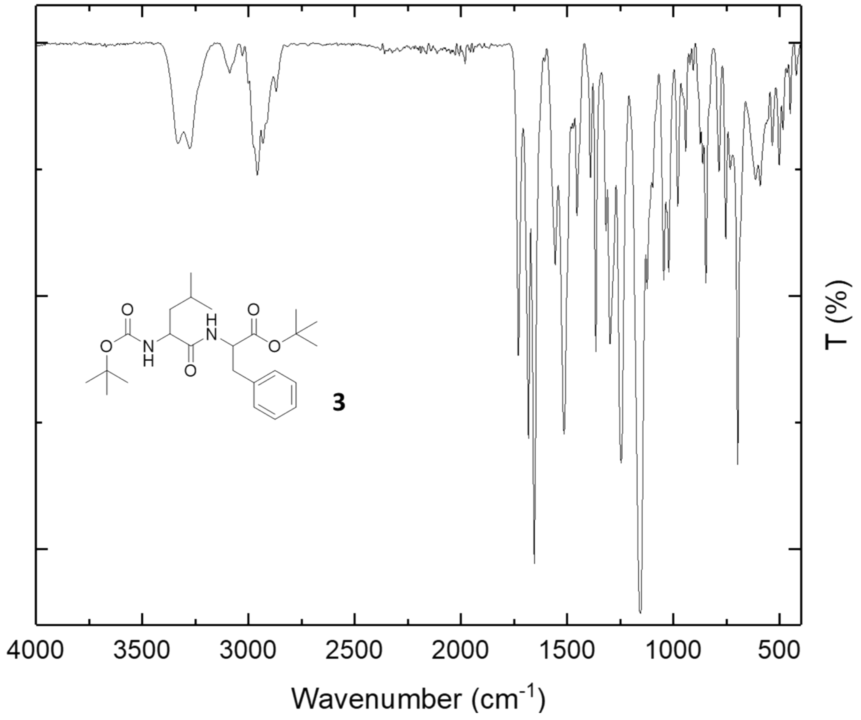

Figure S54. ATR-FTIR spectrum of Boc-Leu-Phe-O*t*Bu **3**.

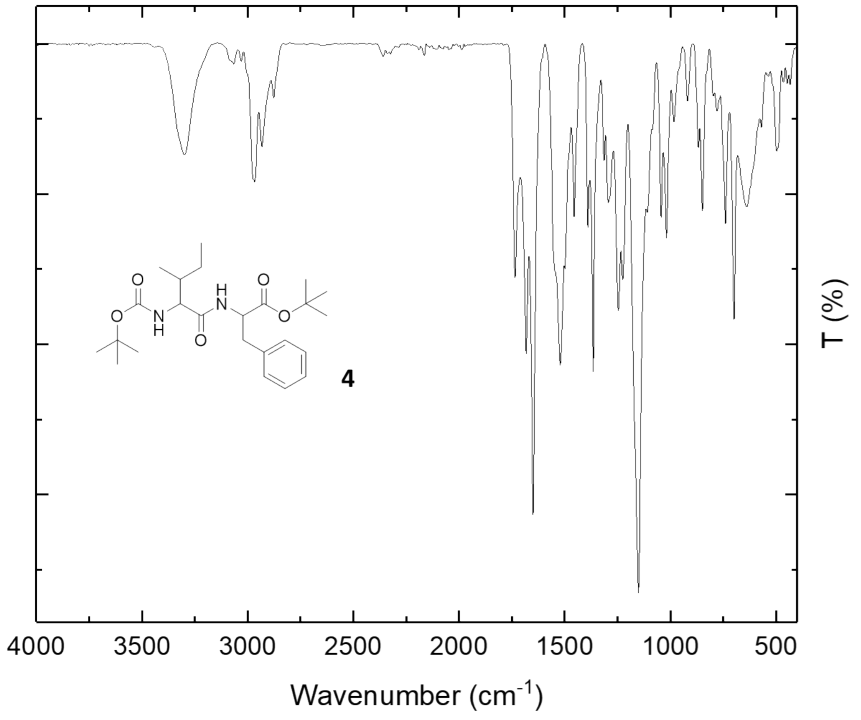

Figure S55. ATR-FTIR spectrum of Boc-Ile-Phe-O*t*Bu **4**.

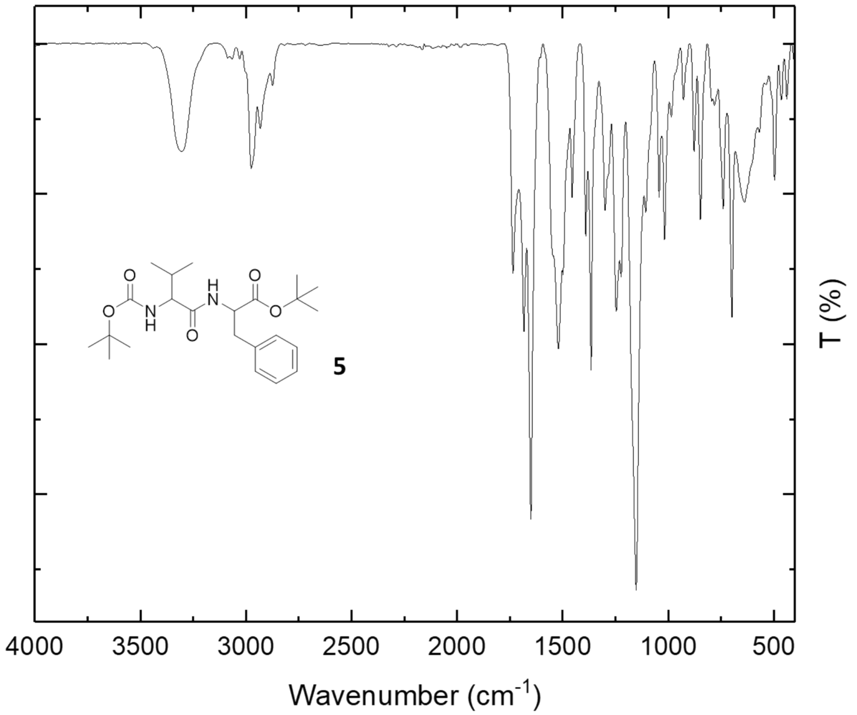

Figure S56. ATR-FTIR spectrum of Boc-Val-Phe-O*t*Bu **5**.

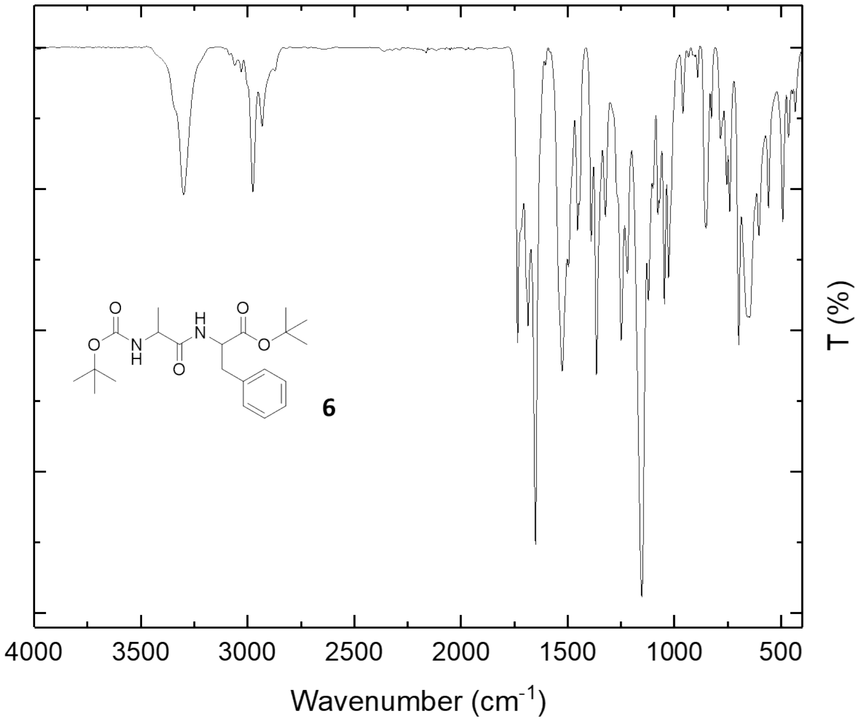

Figure S57. ATR-FTIR spectrum of Boc-Ala-Phe-O*t*Bu **6**.

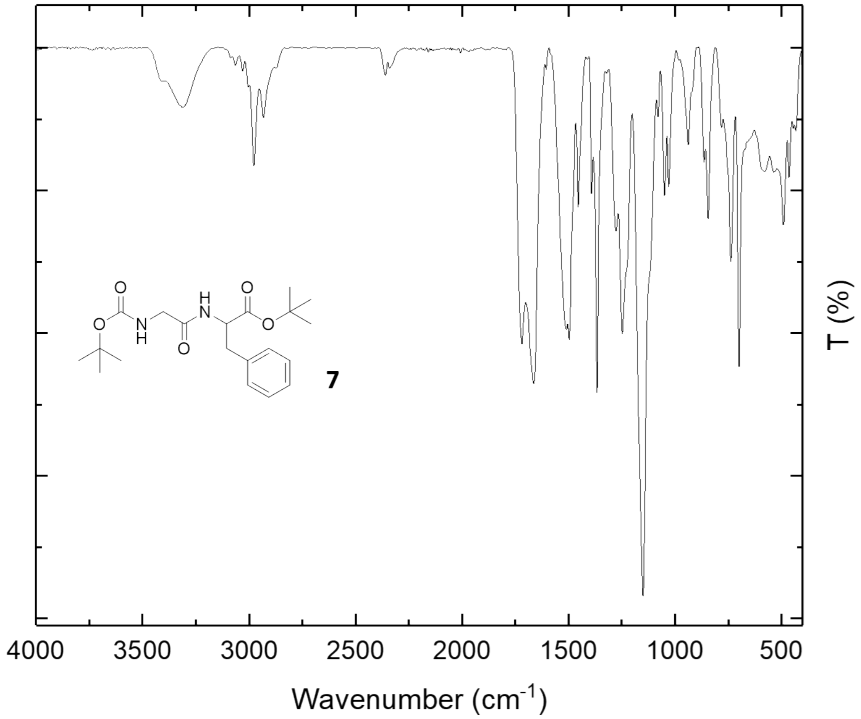

Figure S58. ATR-FTIR spectrum of Boc-Gly-Phe-O*t*Bu **7**.

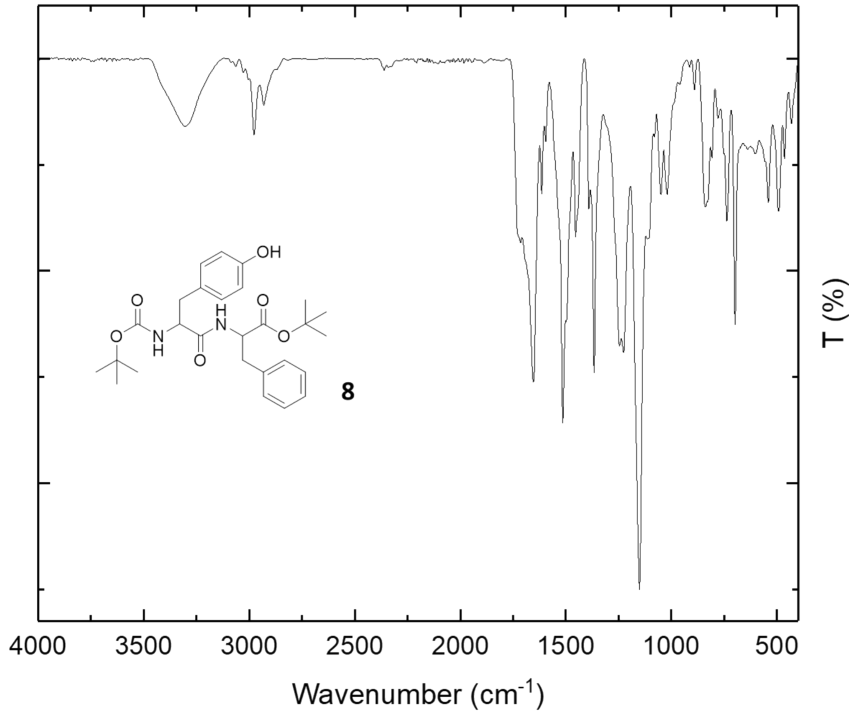

Figure S59. ATR-FTIR spectrum of Boc-Tyr-Phe-O*t*Bu **8**.

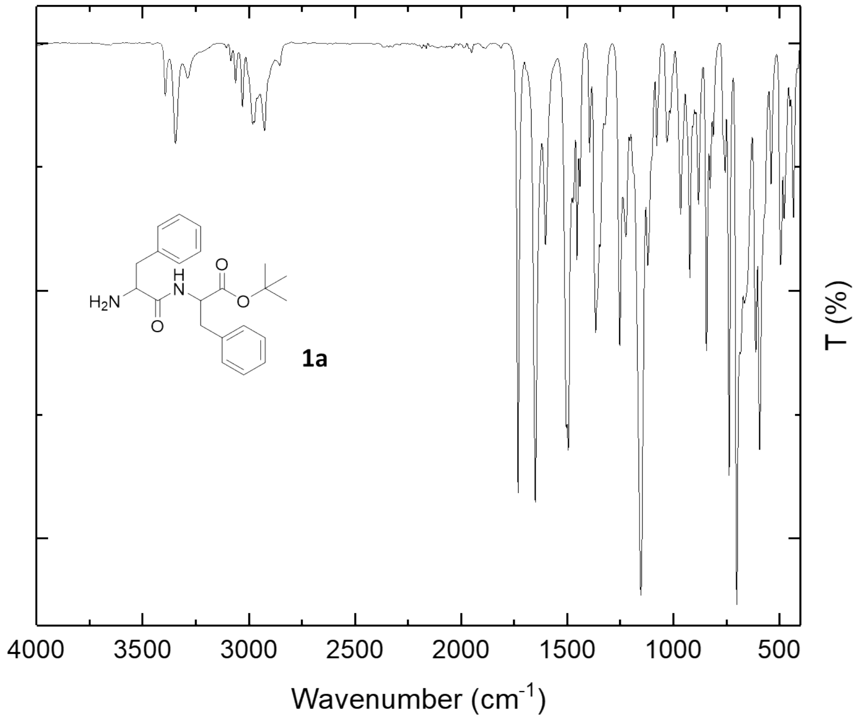

Figure S60. ATR-FTIR spectrum of Phe-Phe-O*t*Bu **1a**.

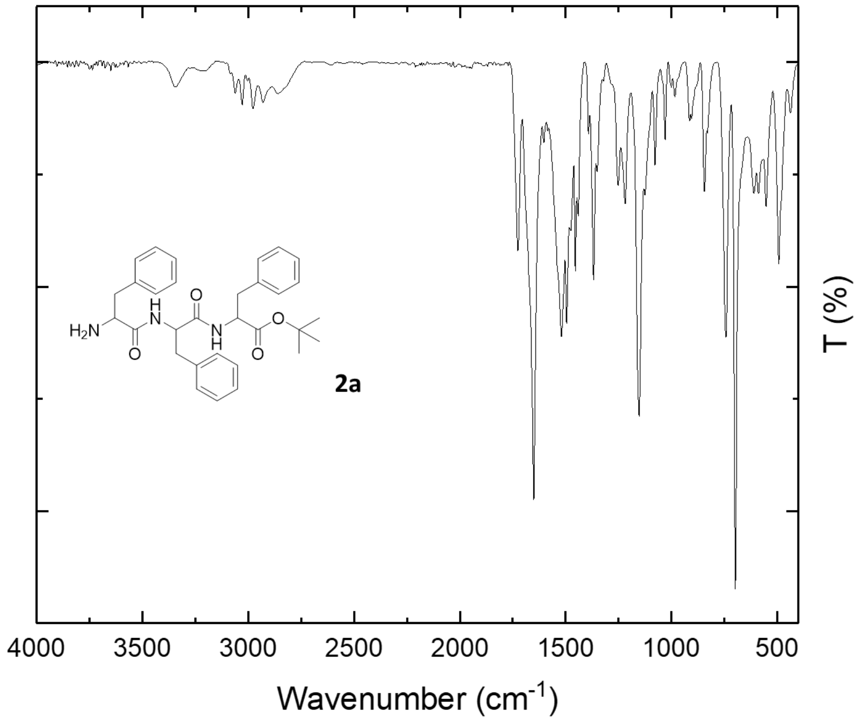

Figure S61. ATR-FTIR spectrum of Phe-Phe-Phe-O*t*Bu **2a**.

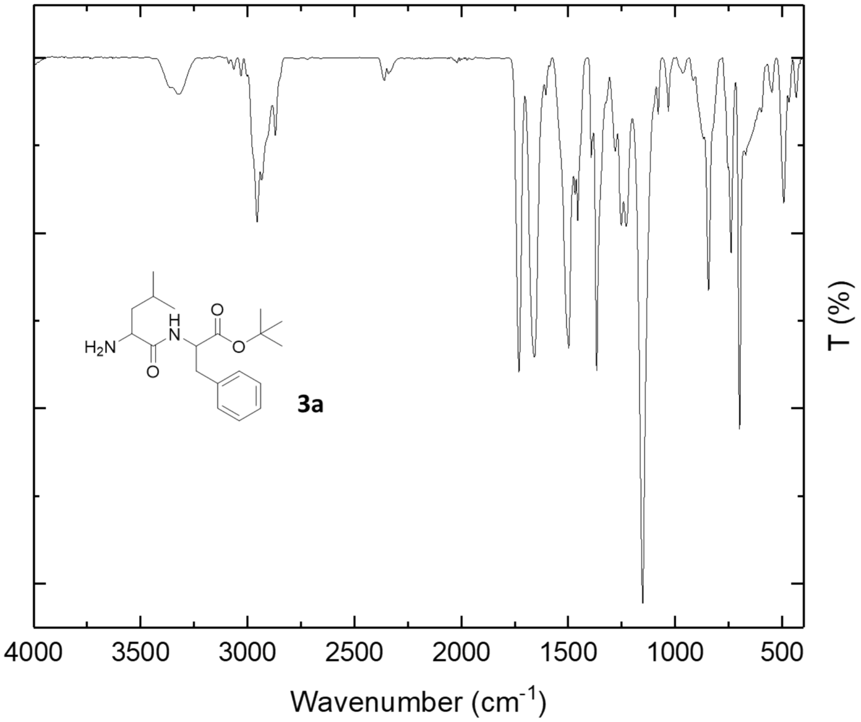

Figure S62. ATR-FTIR spectrum of Leu-Phe-O*t*Bu **3a**.

a)

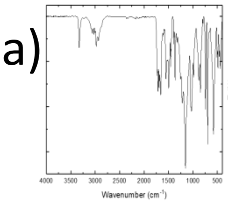

b)

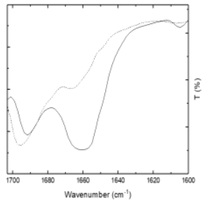

Figure S63. a) ATR-FTIR spectrum of xerogel **I** and b) ATR-FTIR spectrum magnified in the amide I region of xerogel **I** (solid line) and non-dried "wet" gel **I** (dashed line).

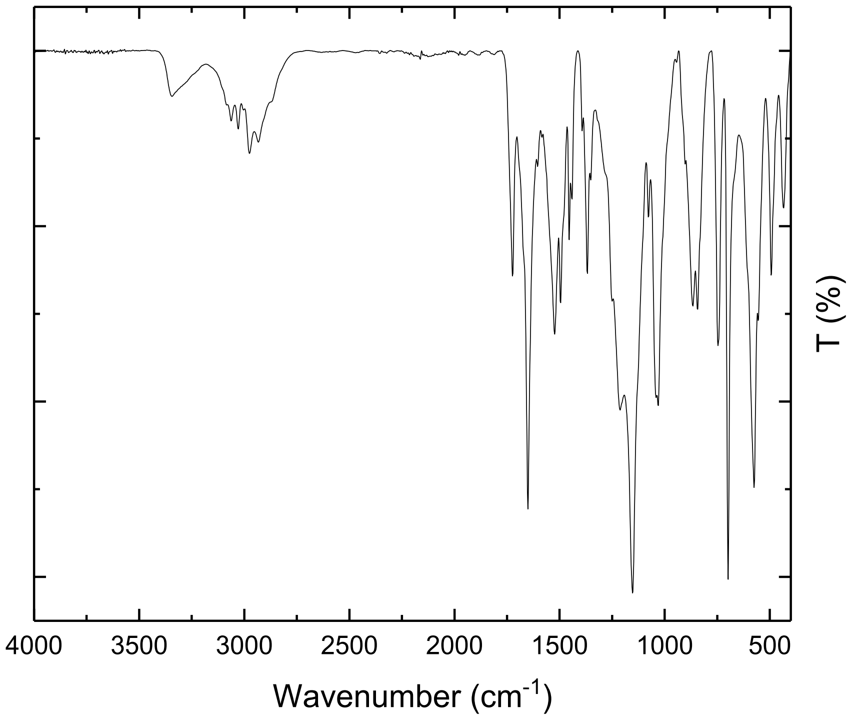

Figure S64. ATR-FTIR spectrum of xerogel **II**.

a)

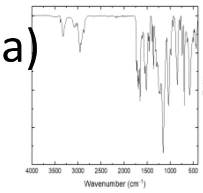

b)

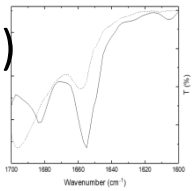

Figure S65. a) ATR-FTIR spectrum of xerogel **III** and b) ATR-FTIR spectrum magnified in the amide I region of xerogel **III** (solid line) and non-dried “wet” gel **III** (dashed line).

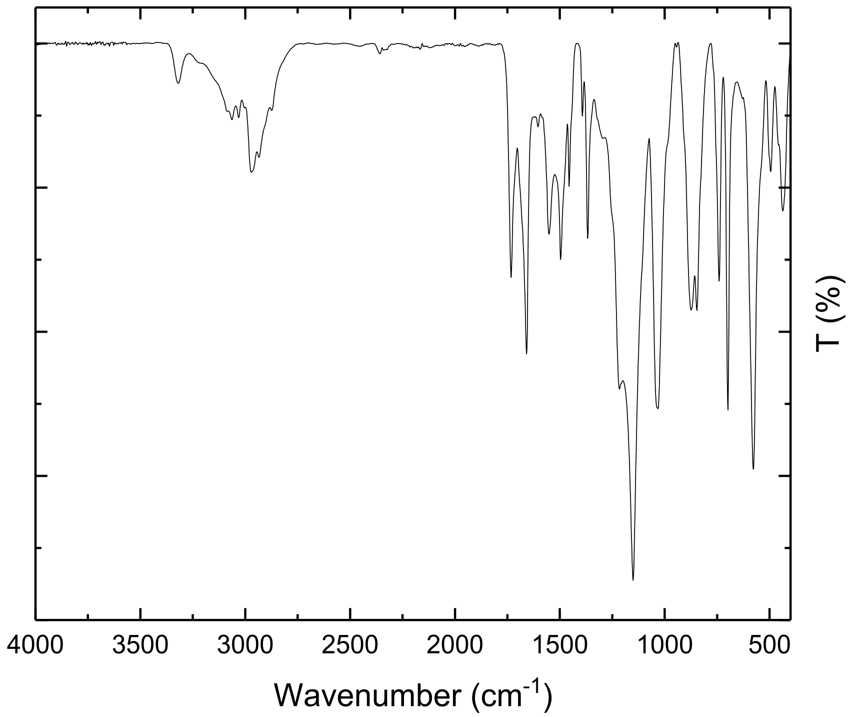

Figure S66. ATR-FTIR spectrum of xerogel **IV**.

4. UV-vis spectra of compounds 1-1a, 2-2a and 3-3a

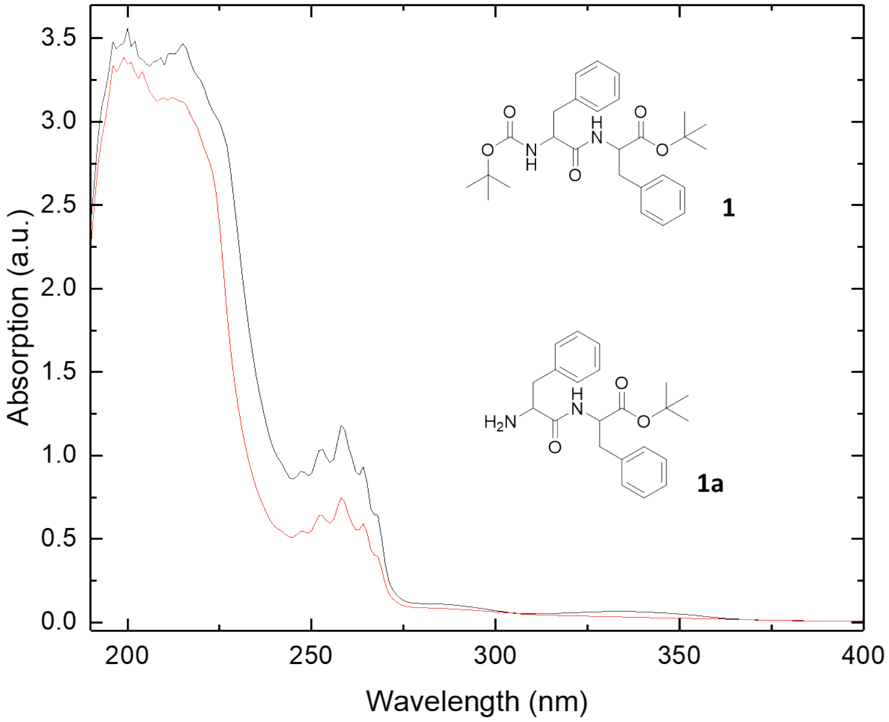

Figure S67. UV-vis spectra of Boc-Phe-Phe-O*t*Bu **1** (black) and Phe-Phe-O*t*Bu **1a** (red) at 25 mM in ACN.

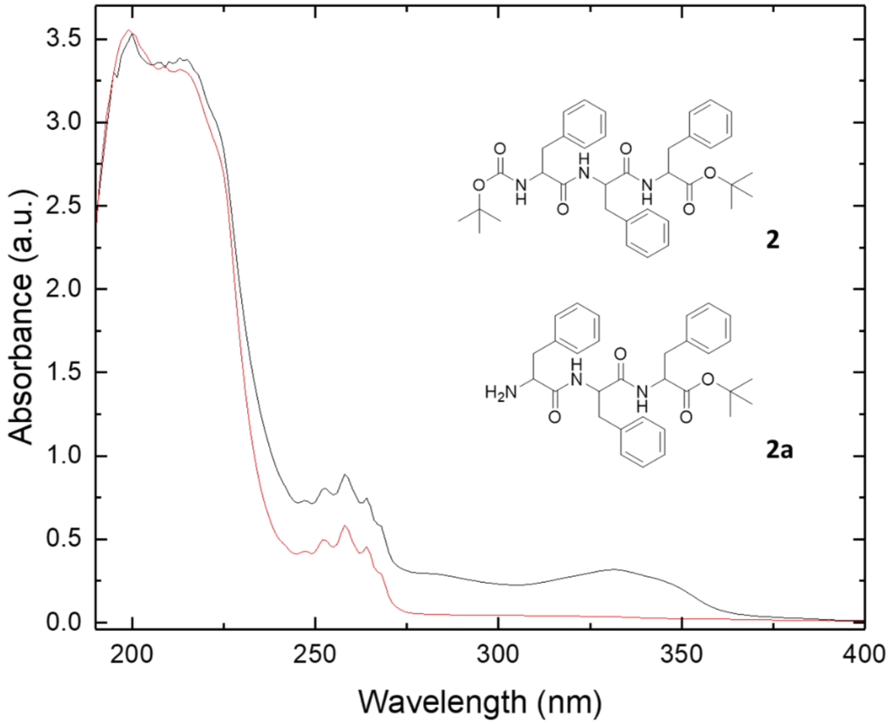

Figure S68. UV-vis spectra of Boc-Phe-Phe-Phe-O*t*Bu **2** (black) and Phe-Phe-Phe-O*t*Bu **2a** (red) at 1 mM in ACN.

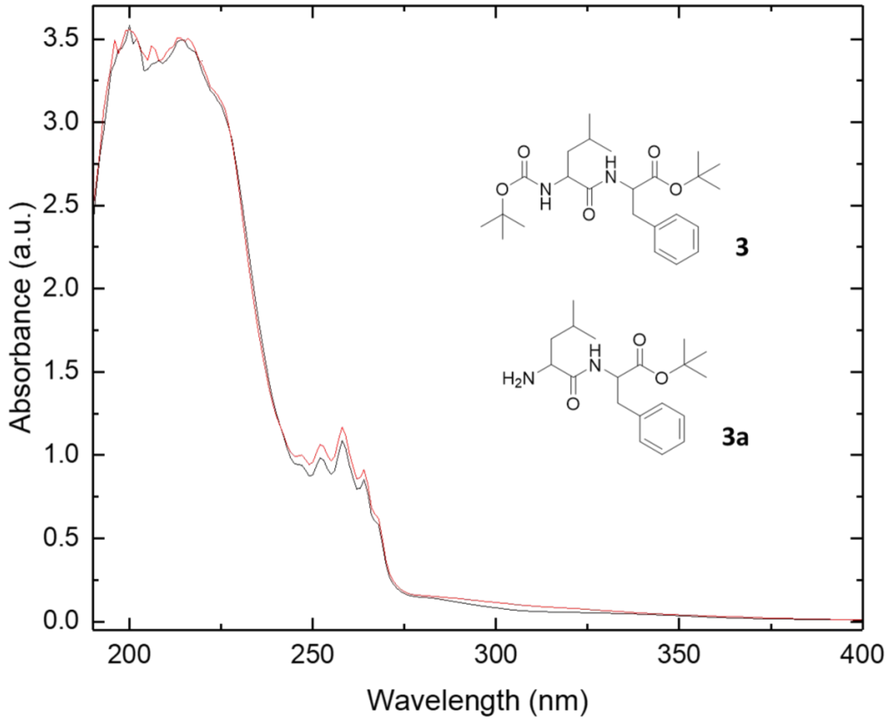

Figure S69. UV-vis spectra of Boc-Leu-Phe-O*t*Bu **3** (black) and Leu-Phe-O*t*Bu **3a** (red) at 5 mM in ACN.

5. Rheology measurement plots of gel systems I, II, III and IV

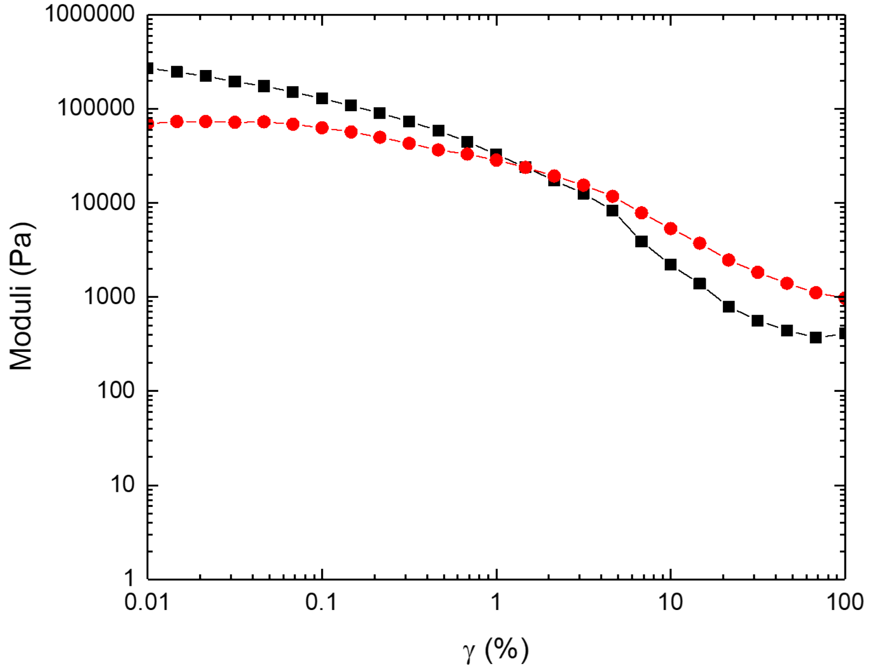

Figure S70. Amplitude sweep measurement of gel system **I** (0.05 M, 1.0 equivalent of H_2SO_4). Storage modulus G´ (black curve, square points) and loss modulus G´´ (red curve, circle points).

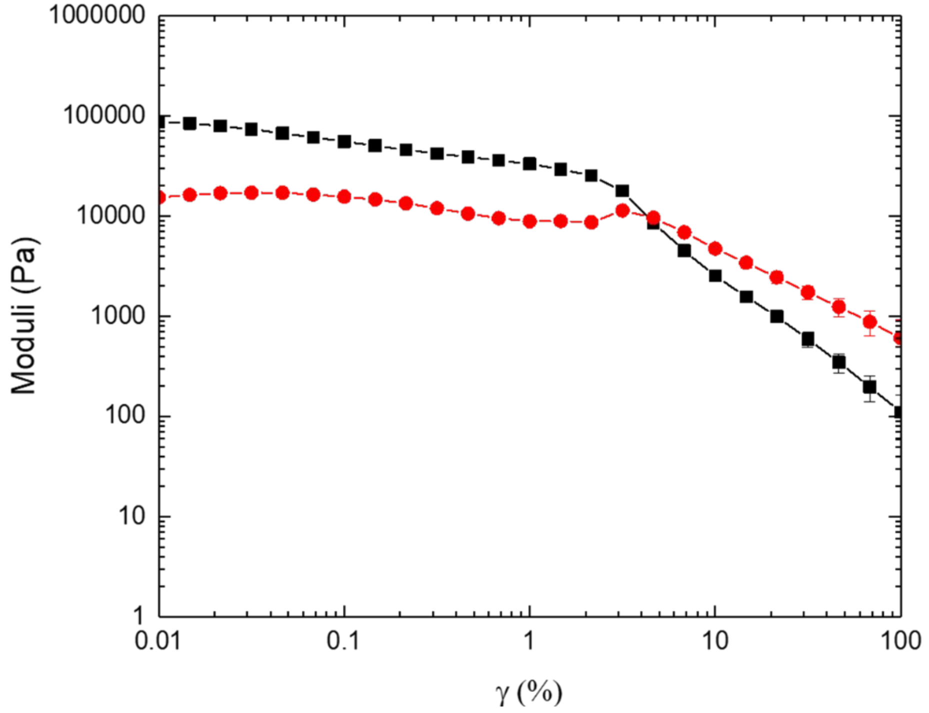

Figure S71. Amplitude sweep measurement of gel system **I** (0.05 M, 0.5 equivalent of H_2SO_4). Storage modulus G´ (black curve, square points) and loss modulus G´´ (red curve, circle points).

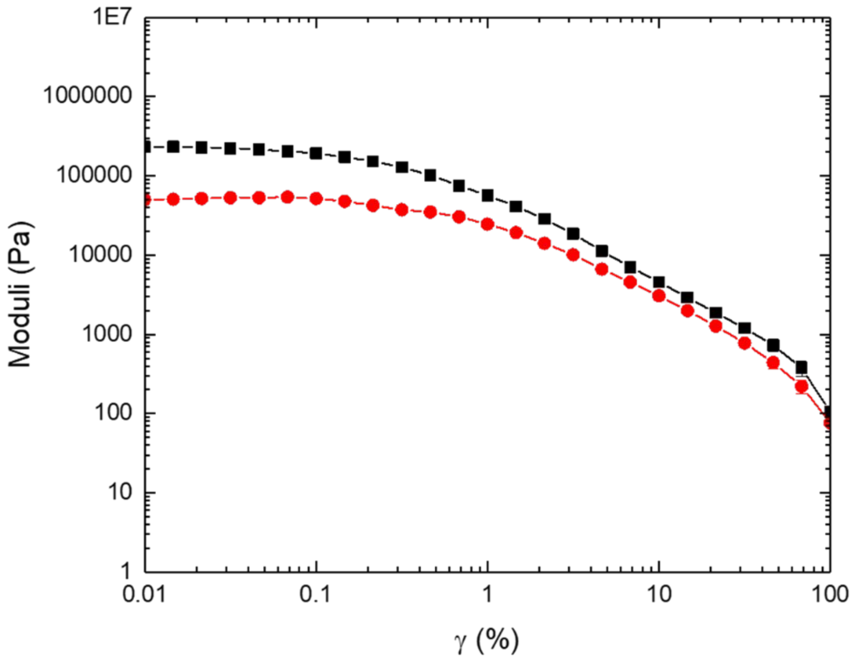

Figure S72. Amplitude sweep measurement of gel system **II** (0.05 M, 1.0 equivalent of H_2SO_4). Storage modulus G´ (black curve, square points) and loss modulus G´´ (red curve, circle points).

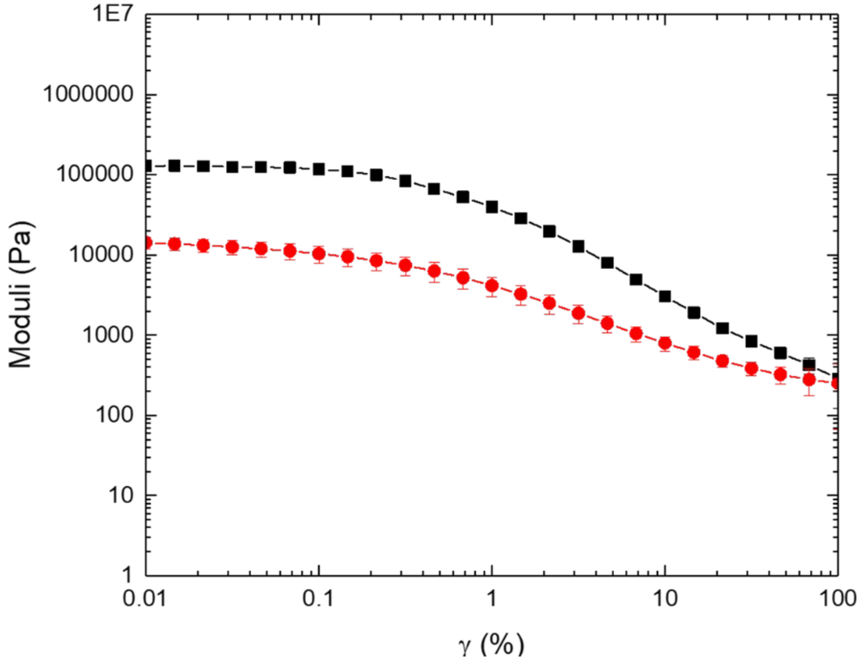

Figure S73. Amplitude sweep measurement of gel system **II** (0.05 M, 0.5 equivalent of H_2SO_4). Storage modulus G´ (black curve, square points) and loss modulus G´´ (red curve, circle points).

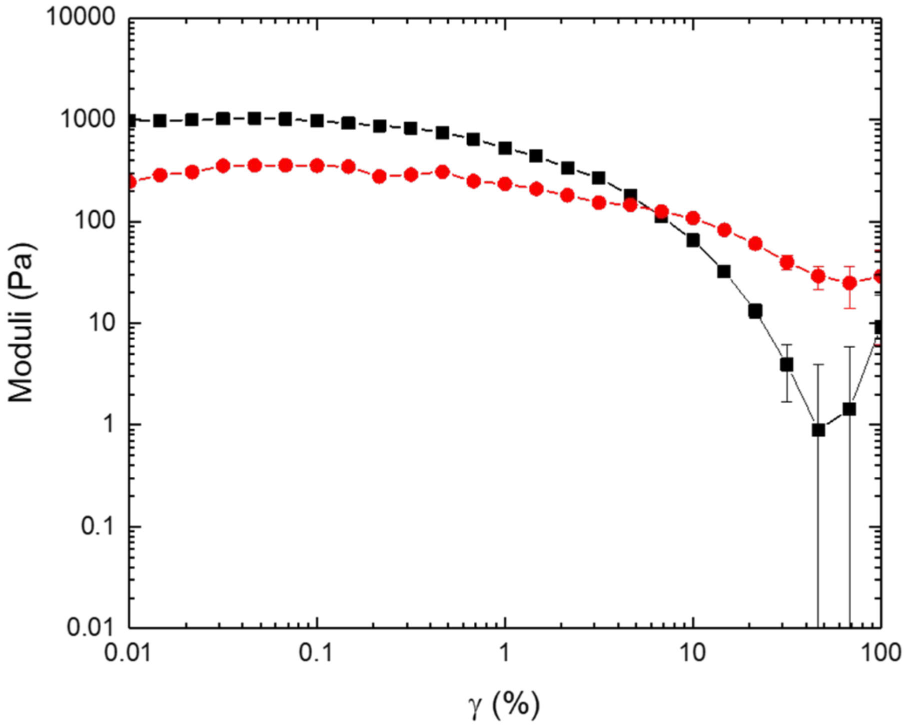

Figure S74. Amplitude sweep measurement of gel system **III** (0.025 M, 1.0 equivalent of H_2SO_4). Storage modulus G´ (black curve, square points) and loss modulus G´´ (red curve, circle points).

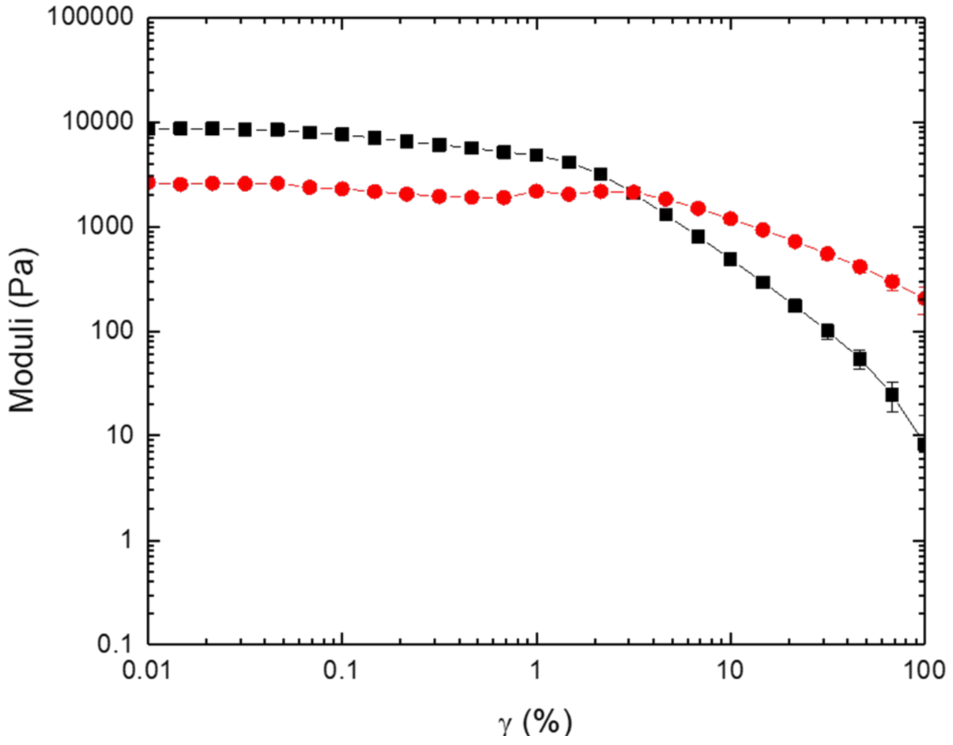

Figure S75. Amplitude sweep measurement of gel system **III** (0.05 M, 1.0 equivalent of H_2SO_4). Storage modulus G´ (black curve, square points) and loss modulus G´´ (red curve, circle points).

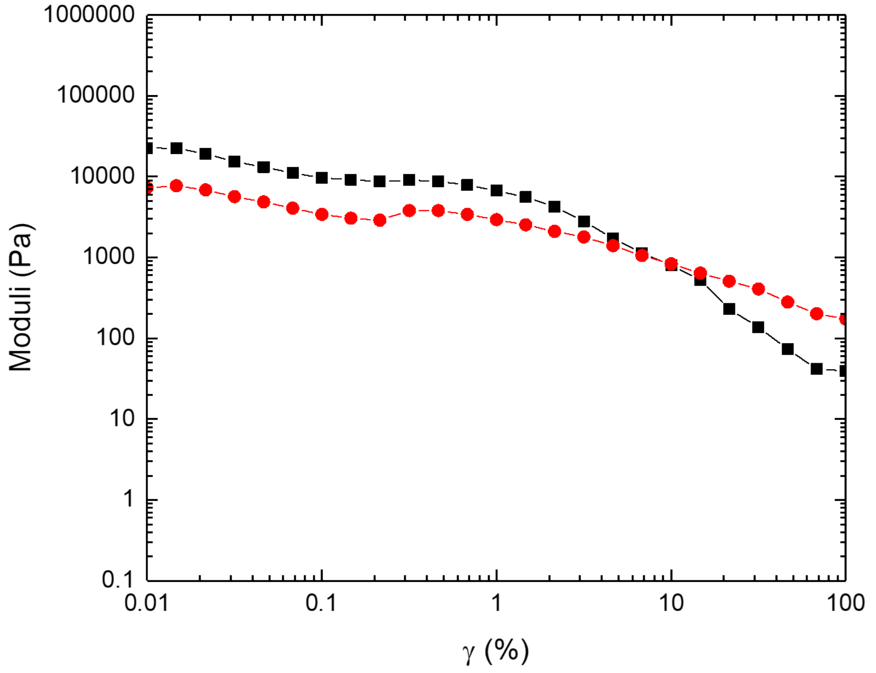

Figure S76. Amplitude sweep measurement of gel system **III** (0.05 M, 0.5 equivalent of H_2SO_4). Storage modulus G´ (black curve, square points) and loss modulus G´´ (red curve, circle points).

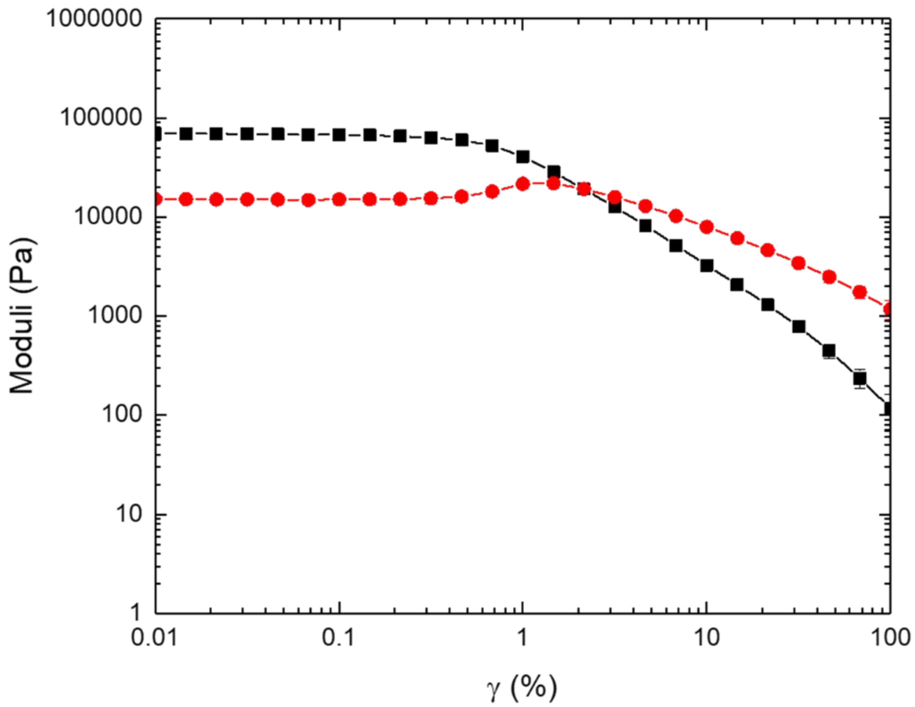

Figure S77. Amplitude sweep measurement of gel system **III** (0.1 M, 1.0 equivalent of H_2SO_4). Storage modulus G´ (black curve, square points) and loss modulus G´´ (red curve, circle points).

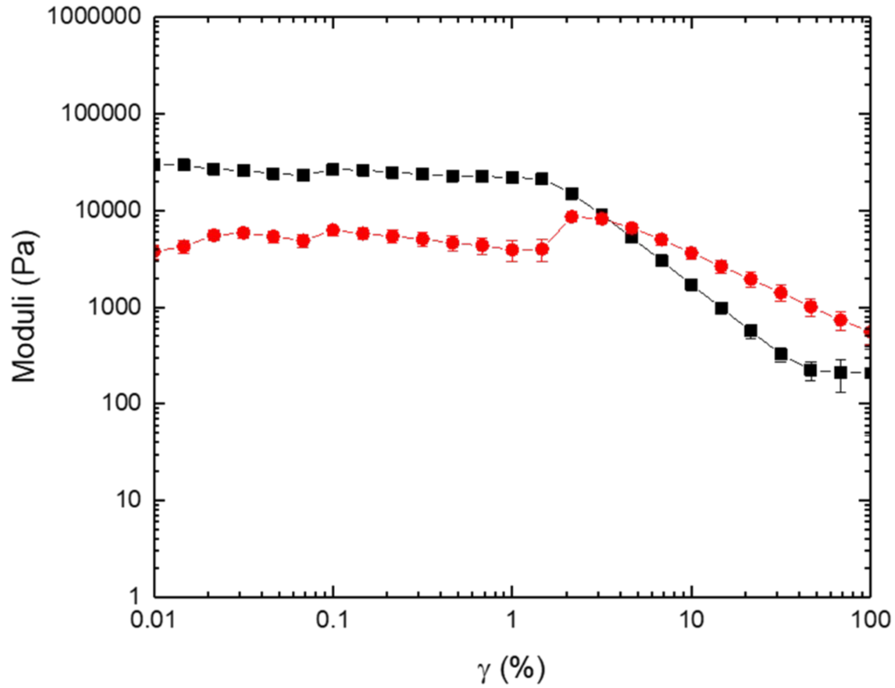

Figure S78. Amplitude sweep measurement of gel system **IV** (0.05 M, 1.0 equivalent of H_2SO_4). Storage modulus G´ (black curve, square points) and loss modulus G´´ (red curve, circle points).

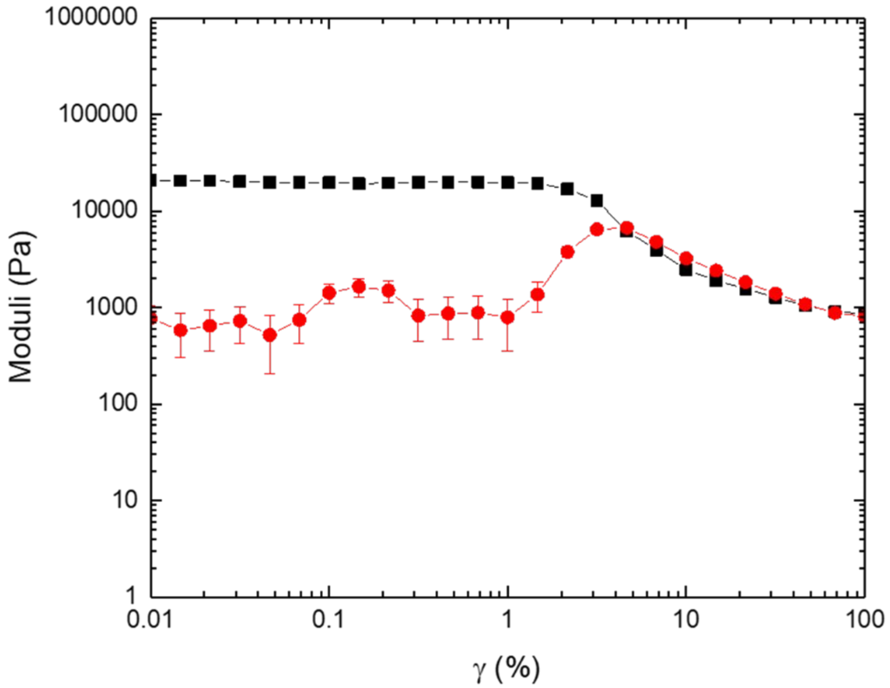

Figure S79. Amplitude sweep measurement of gel system **IV** (0.05 M, 0.5 equivalent of H_2SO_4). Storage modulus G´ (black curve, square points) and loss modulus G´´ (red curve, circle points).

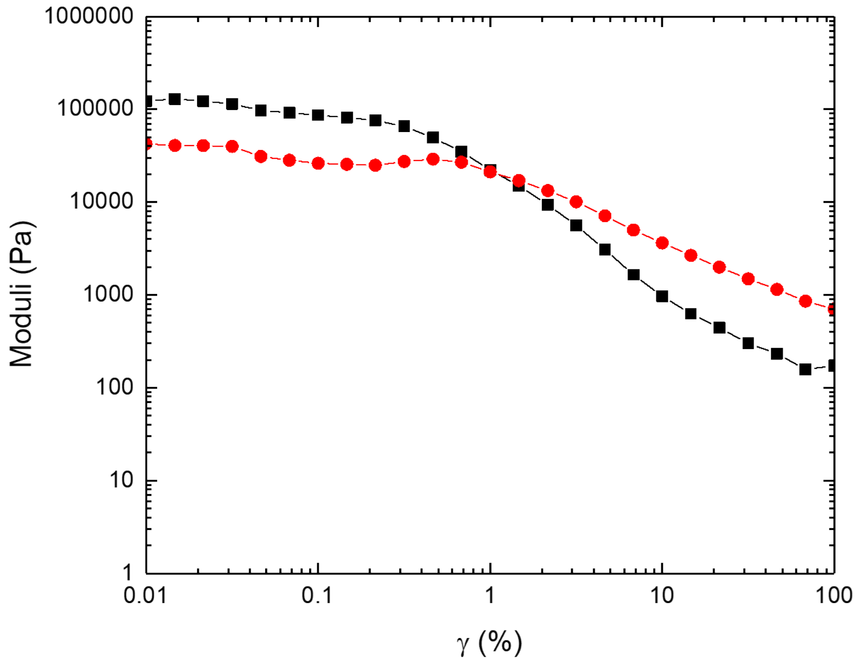

Figure S80. Amplitude sweep measurement of gel system **IV** (0.1 M, 1.0 equivalent of H_2SO_4). Storage modulus G´ (black curve, square points) and loss modulus G´´ (red curve, circle points).
